# Integrated Proteomics Unveils Nuclear PDE3A2 as a Regulator of Cardiac Myocyte Hypertrophy

**DOI:** 10.1161/CIRCRESAHA.122.321448

**Published:** 2023-03-08

**Authors:** Gunasekaran Subramaniam, Katharina Schleicher, Duangnapa Kovanich, Anna Zerio, Milda Folkmanaite, Ying-Chi Chao, Nicoletta C. Surdo, Andreas Koschinski, Jianshu Hu, Arjen Scholten, Albert J.R. Heck, Maria Ercu, Anastasiia Sholokh, Kyung Chan Park, Enno Klussmann, Viviana Meraviglia, Milena Bellin, Sara Zanivan, Svenja Hester, Shabaz Mohammed, Manuela Zaccolo

**Affiliations:** 1 Department of Physiology, Anatomy and Genetics (G.S., K.S., D.K., A.Z., M.F., Y.-C.C., N.C.S., A.K., J.H., K.C.P., M.Z.), University of Oxford, United Kingdom.; 2 Department of Biochemistry (S.H., S.M.), University of Oxford, United Kingdom.; 3 Now with Neuroscience Institute, National Research Council of Italy (CNR), Padova (N.C.S.).; 4 Biomolecular Mass Spectrometry and Proteomics, Utrecht Institute for Pharmaceutical Sciences and Bijvoet Center for Biomolecular Research, Utrecht University, the Netherlands (D.K., A.S., A.J.R.H.).; 5 Centre for Vaccine Development, Institute of Molecular Biosciences, Mahidol University, Thailand (D.K.).; 6 Max-Delbrück-Center for Molecular Medicine in the Helmholtz Association and German Centre for Cardiovascular Research, Partner Site Berlin (M.E., A.S., E.K.).; 7 Department of Anatomy and Embryology, Leiden University Medical Center, the Netherlands (V.M., M.B.).; 8 Department of Biology, University of Padua, Italy (M.B.).; 9 Veneto Institute of Molecular Medicine, Padua, Italy (M.B.).; 10 Cancer Research UK Beatson Institute, Glasgow, United Kingdom (S.Z.).; 11 Institute of Cancer Sciences, University of Glasgow, United Kingdom (S.Z.).; 12 Oxford NIHR Biomedical Research Centre (M.Z.).

**Keywords:** adrenergic agents, hypertrophy, phosphoric diester hydrolases, protein isoforms, rodentia

## Abstract

Signaling by cAMP is organized in multiple distinct subcellular nanodomains regulated by cAMP-hydrolyzing PDEs (phosphodiesterases). Cardiac β-adrenergic signaling has served as the prototypical system to elucidate cAMP compartmentalization. Although studies in cardiac myocytes have provided an understanding of the location and properties of a handful of cAMP subcellular compartments, an overall view of the cellular landscape of cAMP nanodomains is missing.

**Methods:**

Here, we combined an integrated phosphoproteomics approach that takes advantage of the unique role that individual PDEs play in the control of local cAMP, with network analysis to identify previously unrecognized cAMP nanodomains associated with β-adrenergic stimulation. We then validated the composition and function of one of these nanodomains using biochemical, pharmacological, and genetic approaches and cardiac myocytes from both rodents and humans.

**Results:**

We demonstrate the validity of the integrated phosphoproteomic strategy to pinpoint the location and provide critical cues to determine the function of previously unknown cAMP nanodomains. We characterize in detail one such compartment and demonstrate that the PDE3A2 isoform operates in a nuclear nanodomain that involves SMAD4 (SMAD family member 4) and HDAC-1 (histone deacetylase 1). Inhibition of PDE3 results in increased HDAC-1 phosphorylation, leading to inhibition of its deacetylase activity, derepression of gene transcription, and cardiac myocyte hypertrophic growth.

**Conclusions:**

We developed a strategy for detailed mapping of subcellular PDE-specific cAMP nanodomains. Our findings reveal a mechanism that explains the negative long-term clinical outcome observed in patients with heart failure treated with PDE3 inhibitors.

**Novelty and Significance**

**What Is Known?**

- cAMP signaling operates through multiple distinct nanodomains that control different functional effects.
- Phosphodiesterases play a key role in determining which cAMP nanodomain is operative.
- Disruption of cAMP compartmentalization is associated with cardiac disease.
- Only a small number of obvious cAMP nanodomains have been characterized in cardiac myocytes.

**What New Information Does This Article Contribute?**

- We have identified multiple novel, nonobvious cAMP nanodomains under the selective regulation of PDE2A2 (phosphodiesterase2A2), PDE3A1, and PDE3A2.
- We have characterized a nuclear domain that involves PDE3A2, Smad4 (SMAD family member 4), and HDAC-1 (histone deacetylase 1) that controls cardiac myocyte hypertrophy via PKA (protein kinase A)-dependent regulation of HDAC-1 activity.

The compartmentalized nature of cAMP signaling is now well established; however, the full picture of how this system is organized within the cardiac myocyte is missing and only a handful of obvious cAMP nanodomains have been characterized so far. Here, using an integrated phospho-interactomics approach, we discovered multiple novel and nonobvious cAMP nanodomains that are under the regulation of specific phosphodiesterase isoforms. When applied at scale, this method has the potential to uncover the full landscape of subcellular cAMP compartments. We validated, both in rat and human cardiac myocytes, one of the newly identified nanodomains that is located in the nucleus. We demonstrated that, by degrading cAMP at this location, PDE3A2 protects the cardiac myocyte from hypertrophic growth, whereas PDE3A2 inhibition triggers hypertrophy. This cAMP nanodomain can explain the increased mortality observed when PDE3 inhibitors are used to treat heart failure.

Defective cAMP signaling through the β-adrenergic system is a key determinant of exercise intolerance and reduced quality of life in cardiac patients. The exhaustive mapping of subcellular cAMP nanodomains and a detailed understanding of their regulation and functional role will provide an invaluable framework for the development of targeted therapeutics with reduced side effects.

**In This Issue, see p 791**

**Meet the First Author, see p 792**

The intracellular second messenger 3′, 5′-cAMP regulates multiple cellular functions in all organ systems. In the heart, cAMP mediates the cardiac flight-or-fight response to sympathetic activation of β-ARs (β-adrenergic receptors), leading to modulation of rate and strength of contraction. cAMP is synthesized by adenylyl cyclases and is degraded by cyclic nucleotide-hydrolyzing PDEs (phosphodiesterases). PDEs are a superfamily of enzymes that includes 11 gene families (PDE1–11) and >100 protein isoforms, each presenting with a unique combination of substrate specificity, kinetics, regulatory mechanisms, expression profile, and subcellular localization.^1^ Cardiac myocytes express several isoforms belonging to the PDE1, PDE2A, PDE3, PDE4, and PDE8 cAMP-hydrolyzing PDE families.^2^

PDEs play a key role in cAMP subcellular compartmentalization by spatiotemporal regulation of cAMP signals, underpinning specificity of response.^3^ The signaling pathway is organized in subcellular domains^4^ where multiple components involved in cAMP signal transduction are organized in signalosomes. In these multiprotein complexes, the cAMP effector PKA (protein kinase A) is anchored, often via interaction with an AKAP (A kinase anchoring protein), in close proximity to a specific phosphorylation target, and the local level of cAMP is regulated by ≥1 PDE isoforms.^5^ PDEs control the concentration of cAMP within a radius of a few tens of nanometers,^4,6,7^ thus generating submicroscopic subcellular compartments with distinct cAMP levels (nanodomains). By locally degrading cAMP at specific signalosomes, the PDEs contribute to trigger a specific cellular response to a specific stimulus.^4,8–10^ The subcellular distribution and activity of PDE isoforms ultimately defines the topography of active subcellular cAMP nanodomains.

cAMP signaling is disrupted in cardiac disease,^2^ and drugs that target this pathway, such as β-AR antagonists, are a mainstay of current therapeutic regimens. However, available medications remain associated with serious side effects and are ineffective in a significant number of patients,^11^ highlighting the need for a better understanding of the signaling events involved. PDE3 inhibitors are approved for the treatment of heart failure (HF), but despite immediate positive inotropic effects, chronic use is associated with increased long-term mortality,^12^ largely limiting their indication to short-term support of refractory, end-stage HF. The molecular mechanisms responsible for the long-term detrimental effects of PDE3 inhibition remain unclear.

The compartmentalized nature of cAMP signaling provides an opportunity for targeted therapeutic intervention^13–15^ with potential benefit in terms of efficacy and reduced side effects. However, developing targeted interventions requires a detailed understanding of the subcellular organization of the cAMP signaling landscape. A comprehensive map of cAMP nanodomains generated by β-adrenergic stimulation and insight into their regulation and function would provide a valuable asset to dissect physiology, identify disease mechanisms, understand interactions with current medications, and provide a rational basis for the development of targeted therapeutics.^1^

Here, we used an integrated proteomics approach that combines the analysis of PDE family-specific phosphoproteomes with the analysis of PDE isoform-specific interactomes to infer the subcellular localization and function of so far unidentified cAMP nanodomains elicited by β-AR stimulation of cardiac myocytes. Given their clinical relevance, we focused on nanodomains under the control of PDE3A1 and PDE3A2—the most prevalent PDE isoforms in human myocardium.^16^ Our results demonstrate that the integrated proteomics approach used here is a valid strategy to identify previously unknown subcellular cAMP nanodomains. In depth characterization of one of these domains demonstrates that PDE3A isoforms selectively control cAMP in the immediate vicinity of a nuclear PDE3A/SMAD4 (SMAD family member 4)/HDAC-1 (histone deacetylase 1) complex, resulting in modulation of gene transcription and control of cardiac myocytes hypertrophic growth.

## Methods

### Data Availability

All data generated or analyzed during this study are included in this published article (and its Supplemental Material). The mass spectrometry proteomics data have been deposited to the ProteomeXchange Consortium via the Proteomics Identification Database (PRIDE) partner repository with the data set identifier PXD033773.

#### Chemicals

Cilostamide, forskolin, 3-isobutyl-1-methylxanthine (IBMX), isoproterenol, and norepinephrine were from Sigma-Aldrich; myristoylated PKA inhibitor was from Calbiochem; BAY 60-7550 (BAY) was from Cayman Chemicals; collagenase A was from Roche; pancreatin was from Sigma; laminin (mouse) was from BD Biosciences; PBS, DMEM high glucose, MEM199, horse serum, newborn calf serum, penicillin/streptomycin (10 000 units of penicillin [base] and 10 000 μg of streptomycin [base]/mL), and L-glutamine were from Invitrogen.

#### Antibodies and Fluorescent Reagents

PDE3A (catalog number A302-740A, 1:1000 [Bethyl Laboratory]; catalog number Sc-293446, 1:20 [Santa Cruz Biotechnology]), Flag (catalog number F7425, 1:2000; Sigma), GAPDH (catalog number 60004-1-1g, 1:5000; ProteinTech), Anti-phospho-PKA substrate antibody (catalog number 9621S or 9624, 1:1000; Cell Signaling), SMAD4 (catalog number 46535, GTX01674; Cell Signaling) GATA4 (GATA4 binding protein 4; catalog number 36966, 1:1000; Cell Signaling), HDAC-1 (catalog number 34589, 1:1000; Cell Signaling), lamin B (catalog number 12586, 1:1000; Cell Signaling), lamin A/C (catalog number 4777S, 1:1000; Cell Signaling), actin-1 alpha (catalog number 17521-1-AP; Proteintech), RFP (catalog number ab62341, 1:2000; Abcam), GFP (catalog number ab6556, 1:1000; Abcam), horseradish peroxidase–conjugated goat anti-mouse (catalog number sc2005, 1:5000 [SCBT]; catalog number 705-035-151 [Jackson ImmunoResearch Labs]) or anti-rabbit secondary antibodies (catalog number sc2030, 1:5000 [Santa Cruz Biotechnology]; catalog number 705-035-152 [Jackson ImmunoResearch Labs]), antibodies conjugated with Alexa-Fluor 647 (catalog number A-21235, 1:250; Thermo Fisher Scientific), Alexa-Fluor 488 (catalog number A-11008, 1:250; Thermo Fisher Scientific), and wheat germ agglutinin conjugated with Alexa 488 (catalog number W11261; Invitrogen).

#### Plasmids

Flag SMAD4 (clone Id Ora42717) and Flag HDAC-1 (clone Id Ora13308) were obtained from Genscript. The SMAD4–cAMP universal tag for imaging experiments (CUTie) sensor was generated by cloning SMAD4 in a pEYFPc1-CUTie vector^4^ by using the NheI restriction site. Flag HDAC-1 alanine and aspartate mutants were generated by Genscript using the clone ID Ora13308 as a template.

#### Isolation and Culture of Cardiomyocytes

All animal procedures performed conform to the guidelines from directive 2010/63/EU of the European Parliament on the protection of animals used for scientific purposes. Adult rat ventricular myocytes (ARVMs) from 350 to 375 g both male and female Sprague-Dawley rats were isolated as described previously.^4,17^ For phosphoproteomics and immunoblot analysis, fresh cells in tyrode buffer (130 mmol/L NaCl, 5 mmol/L HEPES, 0.4 mmol/L NaH_2_PO_4_, 5.6 mmol/L KCl, 3.5 mmol/L MgCl_2_, 20 mmol/L taurine, 10 mmol/L glucose) containing 1.4 mmol/L CaCl_2_ were used. Cells were treated with 10× working dilutions that would give final concentrations of 0.5 nM isoproterenol, 1 μmol/L BAY, 10 μmol/L cilostamide, or 100 μmol/L IBMX. After a treatment period of 10 minutes at room temperature, during which the cardiomyocytes were allowed to settle by gravity, the supernatant was taken off and cells were snap frozen in liquid nitrogen and stored at −80 °C until further processing. For viral infection, isolated ARVMs were plated on dishes precoated with 1 µg/cm^2^ laminin in culture medium (MEM 199, 5% fetal bovine serum [FBS], 5 mmol/L creatine, 2 mmol/L L-carnitine, 5 mmol/L taurine, 0.1× insulin-transferrin-selenium-X, 2 mmol/L L-glutamine, 100 units/mL penicillin, and 100 µg/mL streptomycin). Cells were washed with serum-free medium 2 hours after plating and subjected to adenoviral infection with vectors PDE3A1-mCherry, PDE3A2-mCherry, or mCherry only (Vector Biolabs).

Neonatal rat ventricular myocytes (NRVMs) from 2-days-old Sprague-Dawley pups were isolated as described before.^18^ For viral transduction, 2.5 million cells were plated on laminin-coated 6 cm plates or on laminin-coated coverslips in M1 medium (4.5 g D-glucose/L DMEM [Invitrogen] with 17% M199 [Invitrogen], 10% horse serum, 5% newborn calf serum, 2 mmol/L L-glutamine, 100 units/mL penicillin and 100 µg/mL streptomycin). After 18 hours, medium was changed to M2 medium (4.5 g D-glucose/L DMEM [Invitrogen] with 17% M199 [Invitrogen], 5% horse serum, 0.5% newborn calf serum, 2 mmol/L L-glutamine and 100 units/mL penicillin, and 100 µg/mL streptomycin) and cells were allowed to adjust for 2 hours in a 37 °C humidified incubator with 5% CO_2_. The cardiomyocytes were infected with adenoviral vectors encoding PDE2A2-mCherry, PDE3A1-mCherry, PDE3A2-mCherry, or mCherry only (Vector Biolabs). After 4 hours of infection, medium was replaced with fresh M2 medium. For plasmid transfection, the transfectin lipid reagent (Bio-Rad) was used following the manufacturer’s instructions. The following day, cells were used for coimmunoprecipitations, immunofluorescence staining, label-free quantification shotgun proteomics, or cells were treated with norepinephrine (10 μmol/L) and other stimuli, as indicated.

#### Human Induced Pluripotent Stem Cell–Derived Cardiomyocytes

Human induced pluripotent stem cells (hiPSCs; LUMCi027-A-1 line,^19^ LUMC0099iCTRL04 line,^20^ M180 line,^21^ and M398 line^21^) were cultured as described previously. Briefly, the LUMC lines were seeded on vitronectin recombinant human protein and cultured in E8 medium; cells were passaged twice a week using PBS containing EDTA 0.5 mmol/L. RevitaCell supplement (1:200) was added during passaging (all from Thermo Fisher Scientific). The M lines were cultured in mTeSR Plus medium (Stemcell Technologies) on human embryonic stem cell (hESC)-qualified Matrigel (8 µg/cm^2^; Corning)-coated surfaces; cells were passaged twice a week using DPBS (Gibco) for rinsing and accutase (Sigma-Aldrich) for dissociation. The ROCK (Rho Kinase) inhibitor (StemMACS Y27632, 10 µmol/L; Miltenyi Biotech) was added for the first 24 hours after seeding. Differentiation into cardiomyocytes was induced in monolayer as described previously.^22^ Differentiated human inducible pluripotent stem cell-derived cardiac myocytes (hiPSC-CM) were maintained in RPMI 1640 medium with Glutamax and 25 mM HEPES (Gibco) plus B27 supplement (Gibco). PDE3A2 overexpression experiments were performed at day 21 of differentiation by transducing hiPSC-CMs with PDE3A2-mCherry adenovirus. The medium was refreshed 24 hours post-transduction, and the cells were harvested after an additional 24 hours (48 hours post-transduction). Untransduced hiPSC-CMs were used as control.

#### Phosphoproteomics

For quantitative phosphoproteomic analysis, NRVMs, which survive in culture better than ARVMs, were transduced with adenoviral vectors encoding PDE2A2-mCherry, PDE3A1-mCherry, PDE3A2-mCherry, or mCherry only, as described above. Stable-isotope dimethyl labeling^23^ was combined with titanium dioxide–mediated phospho-enrichment^24^ and data-driven decision tree–dependent mass spectrometry.^25^ Paired control and treatment samples from at least 3 different animals were analyzed. In brief, frozen cardiomyocyte pellets were thawed in AmBic/urea lysis buffer (8 mol/L urea, 50 mmol/L ammonium bicarbonate, PhosSTOP phosphatase inhibitor cocktail [Roche], and complete protease inhibitor cocktail [Roche]). Cell lysis was allowed to complete for 30 minutes at 4 °C under constant agitation, and cells were subsequently disrupted using a 21G needle. Tryptic digest of a total of 2 mg cardiomyocyte protein was performed using a 10-kD molecular weight cut off Filter Aided Sample Prep protocol. Disulfide bonds in aliquots of 400 mg protein per filter were reduced with 10 mmol/L tris(2-carboxyethyl)phosphine for 30 minutes and then alkylated with 50 mmol/L chloroacetamide for another 30 minutes in the dark. Proteins were washed twice with 6 mol/L urea in 50 mmol/L triethylammonium bicarbonate. Proteins were then digested; first with 1 μg MS grade Lys-C protease (Pierce) per 40 μg protein dissolved in 6 mol/L urea/50 mmol/L triethylammonium bicarbonate for 8 hours at 37 °C, followed by 1 μg MS grade trypsin per 40 μg protein dissolved in 50 mM triethylammonium bicarbonate for 4 hours at 37 °C. Tryptic peptides were eluted by spinning through the 10-kD molecular weight cut off filter. Filters were washed with 0.1% trifluoroacetic acid (TFA) and then with 50% acetonitrile in 0.1% TFA. The eluate was directly used for on-column stable-isotope dimethyl labeling using SepPak C18 cartridges (Waters) as described previously.^23^ Titanium dioxide–packed tips (Thermo Fisher Scientific) were activated with 5% ammonium hydroxide and preequilibrated with loading buffer (2 mol/L glycolic acid, 10% TFA, and 80% acetonitrile). Samples were acidified by adding loading buffer 1:1, bringing glycolic acid to a final concentration of 1M and TFA to a final concentration of 5%. Samples were loaded onto the titanium dioxide columns at a low flow rate (20 μL/min). After all samples were loaded on the columns, they were washed sequentially with loading buffer, 0.2% TFA in 80% acetonitrile and 20% acetonitrile. The phosphopeptides were eluted twice with 5% ammonium hydroxide and immediately acidified with an equal volume of 20% formic acid. Phosphopeptides were analyzed on Orbitrap Elite (Thermo Fisher Scientific) coupled to an EASY-nano-LC 1000 system (Thermo Fisher Scientific) in which phosphopeptides were initially trapped on a 75-μm internal diameter guard column packed with Reprosil-Gold 120 C18, 3 μm, 120Å pores (Dr. Maisch GmbH) in solvent A (0.1% [vol/vol] formic acid in water) using a constant pressure of 500 bar. Peptides were then separated on a 45 °C heated EASY-Spray column (50 cm×75 μm ID, PepMap RSLC C18, 2 μm; Thermo Fisher Scientific) using a 3-hour linear 8% to 30% (vol/vol) acetonitrile gradient and constant 200 nL/min flow rate. Peptides were introduced via an EASY-Spray nanoelectrospray ion source into an Orbitrap Elite mass spectrometer (Thermo Fisher Scientific). Spectra were acquired with resolution 30 000 m/z; range, 350 to 1500; AGC target, 1×10^6^; and maximum injection time, 250 ms. The 20 most abundant peaks were fragmented using collision induced dissociation (automatic gain control target, 5×10^3^; maximum injection time, 100 ms) or electron-transfer dissociation (automatic gain control cation and anion target, 5×10^3^ and 2×10^5^, respectively; maximum injection time, 100 ms; normalized collision energy, 35%) in a data-dependent decision tree method. Peptide identification and quantitation of heavy-to-light ratios was then performed using MaxQuant (v1.6.1.0).^26^ Groups were analyzed with a multiplicity of 2, accounting for the light and heavy dimethyl labels, and phospho (STY) was specified as a variable modification along with protein oxidation and N-terminal acetylation. Trypsin/P was selected as specific digestion mode with a maximum of 2 missed cleavages. Carbamidomethyl groups were accounted for as fixed modifications, and the match between runs function was enabled with a match time window of 0.7 minutes and an alignment time window of 20 minutes. Downstream data processing was performed using the Perseus software package (v1.6.1.1).^27^

#### Label-Free Quantification Shotgun Proteomics

Following affinity purifications of PDE2A2-mCherry, PDE3A1-mCherry, PDE3A2-mCherry, or mCherry from NRVM lysates using RFP-Trap beads, bound proteins were eluted with urea (8 mol/L) under denaturing conditions. Proteins were digested to peptides for interactome analysis with MS grade Lys-C protease (Pierce) for 4 hours at 37 °C, followed by reduction with 2 μmol/L dithiothreitol for 15 minutes at 65 °C and alkylation with iodoacetamide for 30 minutes at room temperature and in the dark. After dilution of the buffer to 2 mol/L urea, further digest with MS grade trypsin was performed for 16 hours at 37 °C. LC-MS/MS was performed using a Q Exactive Hybrid Quadrupole-Orbitrap Mass Spectrometer (Thermo Fisher Scientific). Mass spectra were searched against the Uniprot *Rattus norvegicus* reference proteome (downloaded on July 6, 2014) and quantified using MaxQuant (v1.5.0.0).^23^ Settings were as follows: 2 missed cleavages, carbamidomethylation on cysteine residues as fixed modification, protein oxidation, and N-terminal acetylation as variable modification. The search space was limited to a peptide mass tolerance of 20 ppm and an MS/MS mass tolerance of 0.5 Da. The match between runs function was enabled with a match time window of 0.5 minutes and an alignment time window of 20 minutes. Downstream data processing was performed using the Perseus software package (v1.5.5.3).^24^

#### Network Analysis

Area-proportional Venn diagrams were created using BioVenn.^25^ Gene ontology term analysis for phosphoproteome and interactome target lists was performed using the ClueGO Cytoscape plug-in (v2.5.8).^26^ Category enrichment analysis of PDE3A2, PDE3A1, and PDE2A2 interactors and PDE3 and PDE2A upregulated phosphoproteins was performed with STRING,^27^ using gene ontology cellular component (GOCC) and default parameters. Next, proteins annotated to the GOCC term nucleus were extracted from the list of PDE3A2, PDE3A1 interactors, and PDE3A upregulated phosphoproteins. Common nucleus-annotated PDEA1 and PDEA3 interactors and nucleus-annotated PDE3A upregulated phosphoproteins were used to query STRING for functional and physical interactions. The following settings were used: network type: full STRING network; active interaction sources: experiments and databases; minimum required interaction score: 0.900.

#### Immunofluorescence

NRVMs plated on coverslips were treated with dimethyl sulfoxide (DMSO) or leptomycin B (100 nmol/L) for 3 hours, washed 3× with PBS, and fixed with 4% PFA in PBS at room temperature for 15 minutes. After permeabilization with PBS+0.1% Triton-X-100 (PBST) for 30 minutes, the coverslips were blocked in PBST+10% FBS for 1 hour at room temperature. Primary antibody incubations were performed overnight at 4 °C, followed by 3 washes at room temperature with PBS+0.025% Tween 20 and detection with Alexa-Fluor conjugated secondary antibodies for 1 hour before staining with DAPI (1 μg/mL) for 10 minutes. Coverslips were mounted in Ibidi mounting media and sealed with nail polish. Sequential confocal images were acquired on a confocal laser scanning microscope (TCS SP5 II; Leica, Germany). Nuclear and cytoplasmic fluorescence intensity was calculated by drawing a region of interest with ImageJ V1.53K.^28^

#### Immunoprecipitation and Immunoblotting

For immunoprecipitations from ARVMs or hiPSCs-CM, cells were infected with adenovirus Ad-HDAC-1 virus (catalog number 1498; Vector Biolabs) including an mCherry reporter or PDE3A2-mCherry or mCherry alone (Vector Biolabs). When using hiPSC-CMs, 21-day-old cells were transduced with adenoviral vector. After 24 hours post-transduction, the medium was refreshed, and the cells were treated with cilostamide (10 μmol/L), cilostamide (10 μmol/L) in combination with the PKA inhibitory peptide PKA inhibitor (20 μmol/L), isoproterenol (0.5 nmol/L), a saturating stimulus (25 μmol/L forskolin and 100 μmol/L IBMX), or control medium (DMSO) for 30 minutes and immediately harvested. hiPSC-CMs were dissociated using the multitissue dissociation kit 3 (Miltenyi Biotec) following the manufacturer’s instructions. The cell pellet was washed 3× using ice-cold PBS and then stored at −80 °C after snap freezing in liquid nitrogen to preserve protein integrity. NRVM and HEK293 (Human Embryonic Kidney 293) cells were transfected with plasmid carrying FLAG-HDAC-1. Twenty-four hours post-transfection, the cells were treated with cilostamide (10 μmol/L), a saturating stimulus (25 μmol/L forskolin and 100 μmol/L IBMX), cilostamide 10 μmol/L+PKA inhibitor (10 μmol/L), isoproterenol (0.5 nM), or control medium (DMSO) for 45 minutes and immediately harvested. For immunoprecipitation of the transfected or infected cells, lysates were prepared using an IP buffer (50 mM HEPES pH7.4, 150 mM NaCl, 2 mM EDTA, 10% glycerol, 0.5% Triton-X-100 or 50 mM Tris pH7.4, 150 mM NaCl, 1 mM EDTA, and 1% nonyl phenoxypolyethoxylethanol [NP40]), supplemented with PhosSTOP phosphatase inhibitor cocktail (Roche) and complete protease inhibitor cocktail (Roche). For tissue lysis, isolated left ventricles from rats were homogenized with a tissue homogenizer (Miccra D-4; ART Prozess- & Labortechnik GmbH & Co. KG; Müllheim, Germany) in lysis buffer (10 mM K_2_HPO_4_, 150 mM NaCl, 5 mM EDTA, 5 mM EGTA, 0.5% Triton-X-100, pH7.4, and 0.2% sodium deoxycholate) supplemented with protease and phosphatase inhibitors. Affinity purifications were performed for 2.5 hours using 25 μL RFP-Trap beads (Chromotech) or Flag M2 agarose beads (Sigma) per biological replicate. For immunoprecipitation of endogenous proteins, lysates were incubated overnight at 4 °C with anti-PDE3A or anti-SMAD4 antibodies and protein A sepharose beads (Sigma-Aldrich) or protein A agarose (Thermoscientific) for 6 to 8 hours at 4 °C. For immunoblotting, ARVMs were lysed in AmBic/urea lysis buffer (8 M urea and 50 mM ammonium bicarbonate) supplemented with protease and phosphatase inhibitor cocktails (Roche). Cell debris was removed by centrifugation at 4 °C for 20 minutes at 16 000*g*. Protein concentrations were determined using the Bradford assay (Bio-Rad). Accordingly, 10 to 30 μg of protein per sample was resolved on 4% to 12% Bis-Tris polyacrylamide gels (Invitrogen) or 8% SDS-PAGE gel and transferred to nitrocellulose membranes using the XCell II Blot Module (Thermo Fisher Scientific). To prevent nonspecific binding of the primary antibody, membranes were blocked in 5% milk (Millipore) or phosphoblocker (Cell Biolabs) made in TBST for 1 hour at room temperature. Incubation with the appropriate dilution of primary antibody was performed at 4 °C overnight with gentle agitation. Horseradish peroxidase–conjugated secondary antibodies were used at a dilution of 1:5000. The amount of peroxidase activity remaining on the membrane after washes with TBST was determined using X-Ray Film (GE Healthcare), in combination with Clarity ECL developing solution (Bio-Rad) or Supersignal West Atto (Thermo Scientific). Chemiluminescent signal was also detected by using a ChemiDoc XRS+ (Bio-Rad) imaging system. Quantification of the obtained signal was performed using densitometry analysis with ImageJ V1.53K.^28^

#### RNA Isolation and Analysis

After isolation, NRVMs were washed with PBS, and RNA was isolated using the QIAGEN RNeasy kit, following the manufacturer’s protocol. One microgram of total RNA was used to synthesize cDNA using QuantiTect Reverse Transcription Kit (Qiagen) according to the manufacturer’s instructions. For the quantitative real-time polymerase chain reaction for GATA4, the TaqMan Gene Expression assay primer Rn01530459_m1 and for GAPDH, the TaqMan Gene Expression assay primer Rn01775763_g1 from Applied Biosystems were used. Quantitative real-time polymerase chain reactions were performed in a 7300 Real-Time PCR System (Applied Biosystems). The relative level of GATA4 mRNA was normalized to the DMSO control and respective GAPDH levels. Fold change was calculated using the 2(−ΔΔCt) method.^29^ Total RNA from hiPSC-CMs was extracted using the RNeasy Micro Kit (Qiagen), and 500 ng of RNA was reverse transcribed using the iScript cDNA synthesis kit, following the manufacturer’s instruction. iTaq Universal SYBR Green Supermix was used for cDNA amplification on CFX96 Real-Time PCR Detection System (all from Bio-Rad) using the following conditions: 95 °C 3 minutes/95 °C 30 s; 60 °C 1 minute for 39 cycles/95 °C 10 s; melt curve 60 °C to 95 °C with increment of 0.5 °C. All quantitative PCR (qPCR) reactions were performed in triplicate. Gene expression levels for *GATA4* (forward primer GGCCTGTCATCTCACTACGG; reverse primer ATGGCCAGACATCGCACT), *NPPA* (forward primer CCGTGAGCTTCCTCCTTTTA; reverse primer CCAAATGGTCCAGCAAATTC), and *NPPB* (forward primer CTCCAGAGACATGGATCCCC; reverse primer GTTGCGCTGCTCCTGTAAC) were normalized to DMSO and the correspondent *RPL37A* housekeeping gene (forward primer GTGGTTCCTGCATGAAGACAGTG; reverse primer TTCTGATGGCGGACTTTACCG).

#### Förster Resonance Energy Transfer Imaging

For Förster resonance energy transfer (FRET) measurements, NRVMs were plated on laminin-coated glass coverslips (0.5 µg/cm^2^) and allowed to adhere for 2 hours in a 37 °C humidified incubator with 5% CO_2_. Adenoviral vector encoding the cyclic AMP FRET reporter CUTie was added to the cardiomyocyte medium, and infection was allowed for 3 hours in a 37 °C humidified incubator with 5% CO_2_. The medium was then replaced with fresh FBS-free medium. For SMAD4-CUTie imaging, HEK293 cells or NRVMs were transfected with TransFectin lipid reagent (Bio-Rad), JetPrime (PolyPlus), or FuGENE HD (Promega), following the manufacturer’s instructions. FRET experiments were performed 24 to 48 hours after adenoviral transduction or transfection. Before the experiments, cells were treated with leptomycine B (100 nmol/L) for 3 hours in a 37 °C humidified incubator with 5% CO_2_. For imaging, cells were kept in a modified Ringer saline solution (NaCl 125 mmol/L, KCl 5 mmol/L, Na_3_PO_4_ 1 mmol/L, MgSO_4_ 1 mmol/L, HEPES 20 mmol/L, glucose 5.5 mmol/L, CaCl2 1 mmol/L, and pH7.4) and imaged on an Olympus IX71 inverted microscope using a PlanApoN, ×60, oil immersion objective (numerical aperture 1.4, 0.17/field number 26.5, Olympus, United Kingdom). The microscope was equipped with a CoolSNAP HQ^2^ monochrome camera (Photometrics) and a DV2 Dual-View beam splitter (Photometrics; MAG Biosystems). The FRET filter settings used throughout were CFP excitation filter ET436/20, dichroic mirror 455DCLP (Chroma Technology) in the microscope filter cube, a 505DCLP dichroic mirror, 535/40 YFP (yellow fluorescent protein) and 480/30 CFP (cyan fluorescent protein) emission filters (Chroma Technology) in the beam splitter. Images were acquired and analyzed using MetaFluor 7.1 (Molecular Devices). FRET changes were measured as changes in the background-subtracted 480/535 nm (Epac-S^H187^) or 535/480nm (CUTie and SMAD4 CUTie) fluorescence emission intensity on excitation at 430 nm and expressed as percent FRET ratio change of R/R0, where R is the fluorescence emission ratio at time t and R0 is the average baseline ratio (5–10 samples) short before the first stimulus is added.

#### HDAC Activity

Histone deacetylase activity of HDACs was measured using a fluorometric histone deacetylase kit (CS1010; Sigma-Aldrich) following the manufacturer’s instructions. Equal volumes of purified HDAC-1 beads obtained from immunoprecipitates from cells expressing HDAC-1-Flag as described above were used. For the HDAC assay, 30 μL of assay buffer were added to 100 μmol/L HDAC substrate and 20 μL of washed HDAC-1 beads. After 30 minutes of incubation at 30 °C, 10 μL of the developer solution was added, allowing the fluorescent reporter to be released from the deacetylated substrate. Fluorescence was detected using a Cytation image plate reader (BioTeK) with excitation at 355 nm and emission at 460 nm. Measurements were taken from 4 different biological replicates and normalized to the amount of HDAC-Flag present in each sample, as determined by quantification of a 30-μL sample subjected to Western blotting.

#### In Vitro Hypertrophy and Cell Size Measurements

For NRVM, cells were prepared as described above. Twenty-four hours after isolation, the cells were treated with cilostamide (10 μmol/L), norepinephrine (10 μmol/L), or control medium (DMSO) for 48 hours before cell size determination. For ARVM, cells were suspended in ARVM serum-free medium (MEM 199, 100 units/mL penicillin, 100 µg/mL streptomycin, 5 mmol/L creatine, 2 mmol/L L-carnitine, 5 mmol/L taurine, insulin-transferrin-selenium-X 1X, and 2 mmol/L L-glutamine) and plated on dishes precoated with 1 µg/cm^2^ laminin. Cells were washed with serum-free medium 2 hours after plating and subjected to adenoviral infection with the vectors PDE3A1-mCherry, PDE3A2-mCherry, or mCherry only (Vector Biolabs). After 24 hours, the cells were treated with norepinephrine (10 μmol/L) or other stimuli as indicated for 24 hours, followed by cell size measurement. hiPSC-CMs were dissociated and seeded at a cell density of 5×10^5^ cells per well in a Matrigel-coated 12-well plate for RNA extraction or at a cell density of 1×10^4^ cells per well on Matrigel-coated black 96-well plate for immunofluorescence. hiPSC-CMs were cultured for 48 hours to allow their recovery after dissociation; then the medium was refreshed and the cells were treated with cilostamide (10 μmol/L), norepinephrine (10 μmol/L), or control medium (DMSO) for 7 days, refreshing the medium every 48 hours. At day 4, cells were then transduced for 24 hours with adenoviral vector encoding mCherry alone or PDEA3A2 DN-mCherry. For determination of cell size, cells were imaged in bright-field mode on an Olympus IX71 inverted microscope, equipped with Olympus 40× UPlanFLN and 60× PlanApoN oil immersion objectives (NA 1.3 and 1.42, respectively) using MetaFluor 7.1 (Molecular Devices). Cell membranes were decorated using 5.0 μg/mL wheat germ agglutinin (Invitrogen) in PBS incubated for 10 minutes at room temperature. When required, epifluorescence images were taken using either a YFP illumination set (excitation filter ET500/30, dichroic mirror T515LP, emission filter ET535/30; Chroma Technology) or an RFP set (excitation filter 559/34, dichroic mirror 585LP, emission filter 630/69; Semrock). Cell surface area was calculated with ImageJ by drawing a region of interest including the cell area and by analyzing the number of pixels within the region of interest.

Hypertrophy experiments with dominant negative PDE3 isoforms were conducted at days 30 to 37 after differentiation start. Cells were seeded at a density of 15 000 cells/cm^2^ on Matrigel-coated glass coverslips and grown for 3 to 5 days before the treatment. Cells were then transduced with adenoviral vector encoding dominant negative PDE3A1 and 3A2-mCherry and mCherry alone as control. Treatment with and without norepinephrin (10 µmol/L) was started at the same time. After 48 hours, cells were imaged in differential interference contrast and area was measured using Metafluor 7.1 (Molecular Devices) for acquisition and analysis. All measured cells were also controlled for effective transduction by detecting the red fluorescence of the cells. Measurements were taken on Nikon Eclipse FN-1, equipped with an Opto-LED fluorescent light source (Cairn-Research) and a CoolSnap HQ^2^ camera (Photometrix). All images were acquired with a 40×/0.8 numerical aperture, long distance water dipping objective (Nikon).

#### In Vivo Adrenergic Challenge and Cardiac Tissue Analysis

Male rats (circa 6–8 months) were used and phenotyped as described.^10^ Osmotic minipumps (Alzet 2ml2; Charles River Wiga, Sulzfeld, Germany) were implanted for administration of saline (0.9% NaCl+0.02% ascorbic acid) or isoproterenol (0.13 mg/kg per hour). On day 14 after the implantation, the hearts were collected, cut along the transverse axis, immediately fixed in 10% formalin, and stored at least for 24 hours. The samples were embedded in the paraffin and cooled overnight at 4 °C. Sections (2 or 5 μm thick) were prepared from paraffin blocks using a microtome and mounted on microscope slides. The samples were rehydrated by deparaffinizing twice in xylene (2×5 minutes) and running through a decreasing ethanol series (100%, 96%, 80%, and 70% for 5 minutes each). For further processing, the slides were washed 3× in 1× PBS. Visualization of cardiac myocytes was performed immediately after rehydration of the samples. After blocking nonspecific binding (60 minutes with 10% nanodomains in 1× PBS at room temperature in a humidified chamber), the directly coupled WGA was applied (1:100 in 10% normal donkey serum, 4 °C, in a humidified chamber). A secondary antibody was not required. After incubation, samples could be covered with Vectashield/DAPI. For picrosirius staining (Morphisto; 13422), the sections were incubated for 60 minutes in the dark, washed 2×5 minutes with 0.005% vinegar water, dehydrated in ethanol series (3×99.8%), and immersed in xylene (2×5 minutes). Finally, the sections were covered with Eukitt.

#### Statistical Analysis

Statistical significance tests for proteomics data were performed within the Perseus proteomics software package (v1.6.1.1). Other statistical tests were performed using Sigma Plot v13.0 (Systat Software, Inc) and Prism 5 or Prism 9 (GraphPadPrism). Data are presented as mean±SEM. For n >10, normal distribution of the experimental data was tested with the Anderson-Darling normality test. For comparisons between 2 groups, Student *t* test was used for normally distributed data, For non-normally distributed data or n ≤10, a Mann-Whitney *U* test (unpaired data) or a Wilcoxon signed-rank test (paired data) was used. For comparison between >2 groups, Kruskal-Wallis test followed by a Dunn multiple comparison (non-normal distribution with nonpaired groups or n≤10) or Friedman test followed by a Dunn multiple comparison (non-normal distribution or n≤10 with paired groups) was performed. To account for dependency, a hierarchical analysis model in R using the lme4 package with clustering for individual cells followed by Bonferroni correction for multiple comparisons was applied (as previously described in the study by Sikkel et al^30^). Non-normally distributed data were log transformed before hierarchical statistical testing. *P* values of <0.05 were considered statistically significant. Number of experimental animals and cells per group were chosen based on our experience in previous experiments. Representative images were chosen to closely match the average values of the respective groups. Additional information on statistical analysis is provided in Appendix S4.

## Results

### PDE Family-Specific Phosphoproteomes

Application of a PDE family-selective inhibitor is expected to result in a cAMP increase only at the subcellular sites where isoforms belonging to that family localize, leading to confined activation of PKA and local substrate phosphorylation. From the analysis of the phosphoproteome upregulated on inhibition of a specific PDE family, it should be possible to gain information on the location of cAMP nanodomains under the control of that particular PDE family. To test the validity of this approach for uncovering novel PDE-specific cAMP nanodomains, we analyzed the phosphoproteome associated with inhibition of PDE3 and compared it to the phosphoproteome associated with inhibition of PDE2A. As our aim was to reveal previously unappreciated cAMP nanodomains elicited by sympathetic stimulation, we applied the PDE family-selective inhibitors in combination with the β-AR agonist isoproterenol. Using the cytosolic FRET biosensor EPAC-S^H187^ to measure cAMP,^31^ we optimized the concentration of isoproterenol so that, on subsequent addition of the PDE inhibitor, the following criteria would be satisfied: (1) generation of a relatively contained increase in cAMP, sufficient to engage the relevant PDE hydrolytic activity but with minimal overspill and disruption of compartmentalization; (2) generation of a comparable overall cellular amount of cAMP on application of the PDE inhibitors, to avoid that differences in target phosphorylation may result from grossly unequal global cAMP levels; (3) generation of a functionally relevant signal, as determined by detectable activation of PKA. Treatment of ARVMs with 0.5 nmol/L isoproterenol satisfies all 3 criteria (Figure S1).

**Figure 1.**
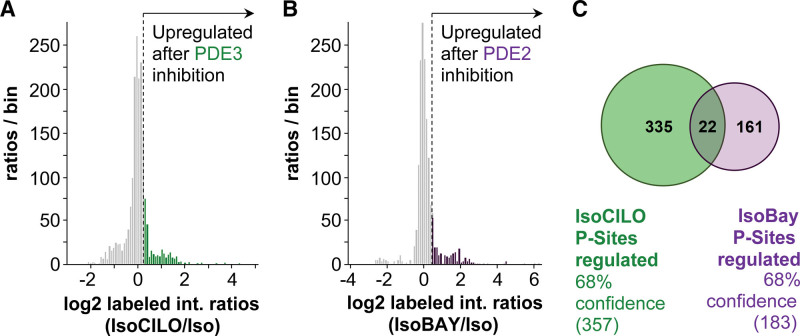
**Analysis of the β-adrenergic phosphoproteome selectively elicited by inhibition of PDE3 (phosphodiesterase 3) or PDE2A. A**, Frequency distributions of log2 intensity ratios to illustrate thresholding of PDE3-dependent and PDE2A-dependent (**B**) phosphoproteomics data for pathway analysis. The top 16% quantified phosphopeptides (highlighted in green and purple for PDE3A and PDE2A, respectively) were carried forward for further analysis. **C**, Venn diagram comparing the 16% upregulated phosphorylation sites in samples treated with the PDE3 inhibitor (green) or the PDE2A inhibitor (purple). The CI (68%) derives from the selected peptides lying outside 1 SD of the mean distribution of peptides, as shown in **A** and **B**, respectively. BAY indicates BAY 60-7550; Cilo, cilostamide; and Iso, isoproterenol.

Cell lysates from ARVMs treated with 0.5 nmol/L isoproterenol alone or in combination with the PDE3 inhibitor cilostamide (10 μmol/L) or the PDE2A inhibitor BAY (1 μmol/L) were prepared for quantitative LC-MS/MS, as shown in Figure S2. For ARVMs treated with cilostamide, proteins were identified after titanium dioxide enrichment, 264 of which were quantified (Figure S3A). For ARVMs treated with BAY, 776 proteins were identified, 270 of which were quantified (Figure S3B). This corresponds to 2231 and 1146 detected phosphorylation sites in cardiomyocytes treated with a combination of isoproterenol+cilostamide or isoproterenol+BAY, respectively (Figure S3C). As prestimulation with isoproterenol lead to considerable baseline phosphorylation, the dynamic range of the observed ratios between PDE inhibitor and control was narrow, with many substrates showing little or no change. For data analysis, we, therefore, applied an intensity-based thresholding approach,^32,33^ considering all phosphorylation sites within the top 16% intensity ratios as differentially regulated (Figure 1A and 1B). A full list of the differentially regulated phosphopeptides is shown in Appendix S1. In line with what is expected, of the quantified upregulated phosphorylation sites, 42% and 41% were annotated as containing a PKA motif in the sample treated with cilostamide and BAY, respectively (Figure S4A and S4B). This enrichment for PKA sites was conserved in the top 16% sites differentially regulated upon inhibitor treatment (35% of the 357 PDE3-regulated sites and 43% of all 183 PDE2A-dependent phosphorylation sites were annotated as PKA sites; Figure S4C and S4D). Of the differentially regulated sites, only 22 sites in 22 different proteins were found in both the PDE3- and the PDE2A-dependent phosphoproteomes (Figure 1C; Table S1), 32% of which contain a PKA motif. These 22 phosphopeptides correspond to only 6% of the differentially regulated phosphorylation sites, supporting the notion that PDE3 and PDE2A enzymes operate largely independently in distinct subcellular compartments and indicating that the analysis of PDE family-dependent phosphoproteomes is a valid approach to identify targets under the control of individual PDE families.

### PDE Isoform-Specific Interactomes

**Figure 2.**
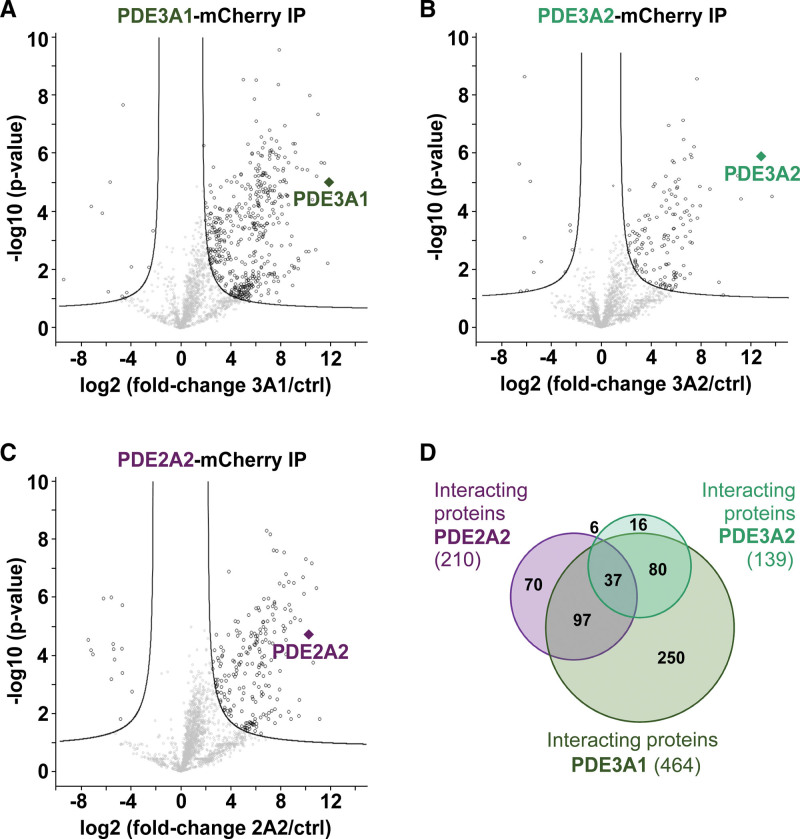
**Analysis of the PDE3A1 (phosphodiesterase 3A1), PDE3A2, and PDE2A2 interactomes. A**, Volcano plot of the PDE3A1-mCherry interactome (false discovery rate [FDR], 0.03; S-zero is the fold-change threshold [S0], 1.3). **B**, PDE3A2-mCherry interactome (FDR, 0.05; S0, 0.8). **C**, PDE2A2-mCherry interactome (FDR, 0.03; S0, 1.3). **D**, Area-proportional Venn diagram to compare significant interactors in PDE3A1, PDE3A2, and PDE2A2 affinity purifications.

Available PDE inhibitors are family-selective but do not discriminate between different PDE isoforms within a family. To increase the resolution of our investigation and gain information on where specific PDE isoforms operate, we analyzed the interactomes of the PDE3 family isoforms PDE3A1 and PDE3A2 and compared them to the interactome of the PDE2A2 isoform. Individual PDE isoforms tagged with mCherry or mCherry alone were expressed in isolated NRVMs. Confocal imaging confirmed that the 3 selected PDE isoforms show largely distinct subcellular localization, with PDE3A1 showing a predominant pattern compatible with internal membranes, PDE3A2 showing a somewhat more homogeneous distribution within the cell, and PDE2A2 showing a distribution in line with the predominantly mitochondrial localization described previously^34^ (Figure S5). After immunoprecipitation and tryptic digest of the coprecipitated proteins, the samples were separately analyzed by mass spectrometry (Figure S6). We identified 1979, 1900, and 1871 proteins for the PDE3A1, PDE3A2, and PDE2A2 interactomes, respectively (Figure S7). *t* test analysis using mCherry alone as control yielded 464 potential interaction partners for PDE3A1, 139 interaction partners for PDE3A2, and 210 interaction partners for PDE2A2 (significance thresholds are false discovery rate [FDR], 0.03 and S-zero is the fold-change threshold [S0], 1.3 for the PDE3A1 interactome; FDR, 0.5 and S0, 0.8 for the PDE3A2 interactome; and FDR, 0.05 and S0, 1.3 for the PDE2A2 interactome; Figure 2). Overall, the data show that, at least for PDE3A1 and PDE2A2, a considerable proportion of the interactors is unique for the specific PDE isoform (Figure 2E). The presence of shared interactors between isoforms is in line with the observation that multiple PDEs can impinge on the same subcellular compartment, where they may have synergistic effect.^35^ A full list of the proteins significantly enriched in the PDE3A1, PDE3A2, and PDE2A2 interactomes is shown in Appendix S2.

### Integrated Analysis of Interactomes and Phosphoproteomes Reveals Potential Novel cAMP Nanodomains

**Figure 3.**
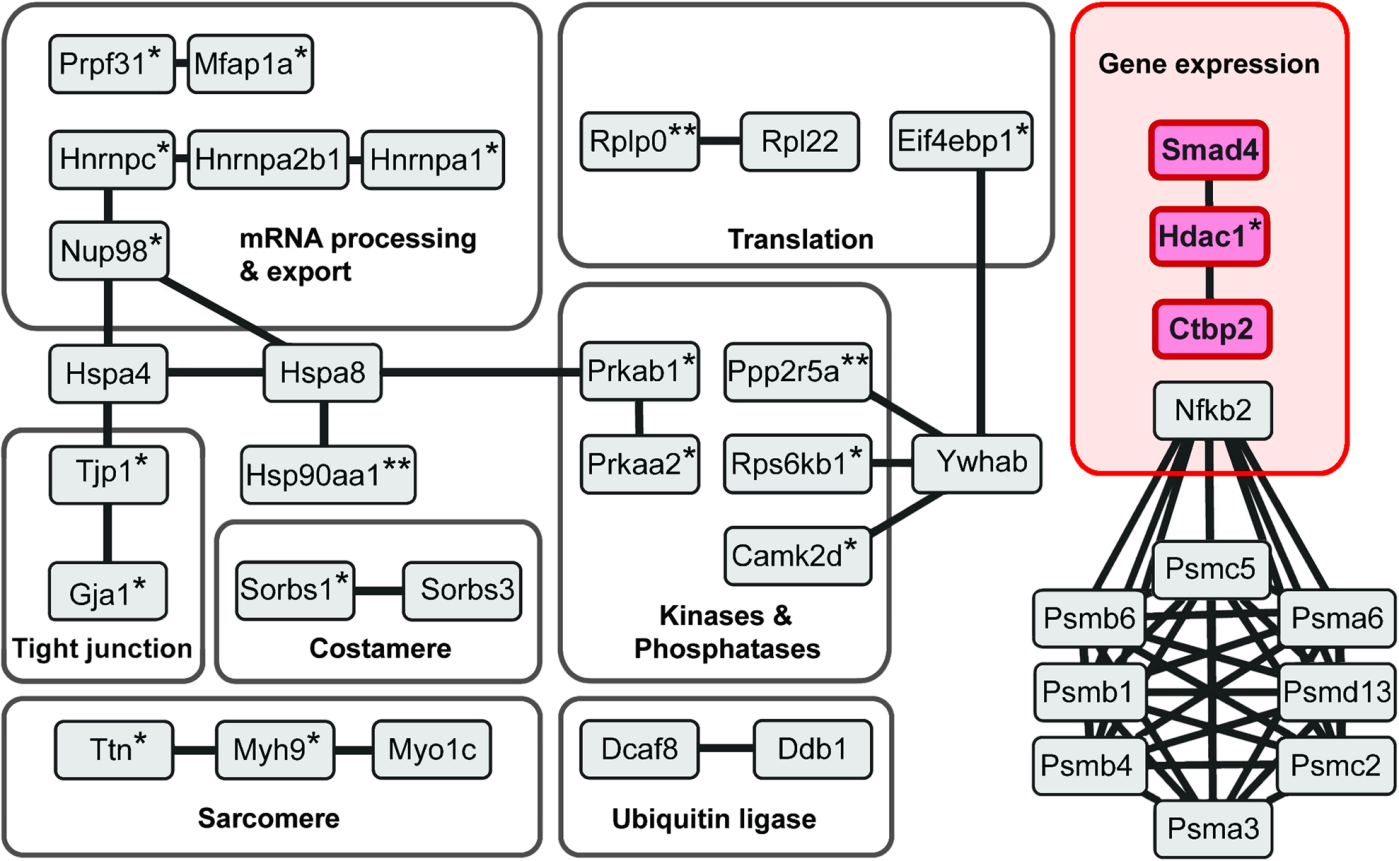
**Nuclear protein interaction network shared by PDE3A1 (phosphodiesterase 3A1) and PDE3A2.** Functional and physical interaction network of PDE3A1/A2 interactors and phosphorylated downstream proteins generated with the STRING software. All proteins shown have been identified experimentally either in the cilostamide-dependent phosphoproteome or in the interactome shared by PDE3A1 and PDE3A2. *Proteins found in the cilostamide-dependent PDE3A phosphoproteome. **Proteins that are both in the cilostamide-dependent phosphoproteome and in the PDE3A isoform interactomes.

Analysis of the interactome and phosphoproteome data for a specific PDE revealed a number of proteins significantly enriched in both data sets (Table 1). If a protein features in the interactome of a specific PDE isoform and the phosphorylation of the same protein is selectively enhanced by treatment with the relevant family-selective inhibitor, it is likely that such protein pinpoints the site of a cAMP nanodomain under the specific control of that particular PDE. In support of this notion, we found that the SERCA2 (sarco-endoplasmic reticulum calcium ATPase 2; *Atp2a2*) appears only in the PDE3A-dependent phosphoproteome and in the PDE3A1 interactome (Table 1), in agreement with previous work reporting interaction of PDE3A with SERCA^36^ and involvement of PDE3A in the regulation of SERCA activity.^37^ Similarly, the NHE1 (Na^+^/H^+^ exchanger 1; *Slc9a1*) appeared selectively in the PDE3A phosphoproteome and in the PDE3A1 interactome, in line with evidence linking cardiac PDE3 and regulation of NHE1.^38^ The PMCA (plasma membrane Ca^2+^-ATPase; *Atp2b1*) appeared in the PDE2A2 interactome and the PDE2A-associated phosphoproteome exclusively, consistent with a role of PDE2A in the regulation of PMCA in cardiac myocytes.^39^ Thus, we suggest that the presence in both the phosphoproteome and interactome data sets can be used as a criterion to prioritize proteins for further validation. Application of a less stringent selection criterion may, however, be equally valuable, as cAMP signalosomes often involve complex multiprotein assemblies and the protein phosphorylated on inhibition of a local PDE may not necessarily interact with the PDE itself. To identify these signalosomes, we performed gene ontology cellular component (GOCC) term enrichment analysis to find terms enriched simultaneously in a PDE isoform interactome and the relevant phosphoproteome to generate functional networks that may reveal nonobvious cAMP nanodomains under the control of specific PDE isoforms. GOCC analysis of our data sets showed a highly significant enrichment for the term nucleus for PDE3 (for the PDE3A1 interactome: 107/6630 genes and FDR, 9.91×10^−43^; for the PDE3A2 interactome: 68/6630 genes and FDR, 7.32×10^−6^; for the PDE3 phosphoproteome: 107/6630 genes and FDR, 4.4×10^−10^; Appendix S3). Next, we used the nucleus-annotated interactors shared by PDE3A1 and PDE3A2 and nucleus-annotated phosphoproteins upregulated on cilostamide treatment as input to generate a functional and physical interaction network using STRING software with stringent settings to pinpoint likely interactions (Figure 3). Among the interactions identified, we focused on the CtBP2 (C-terminal Binding Protein 2)/phospho-HDAC-1/SMAD4 transcription regulatory complex for further validation and tested the hypothesis that PDE3A isoforms selectively regulate a nuclear cAMP nanodomain that involves SMAD4 and HDAC-1 to control gene transcription and hypertrophy.

**Table 1.**
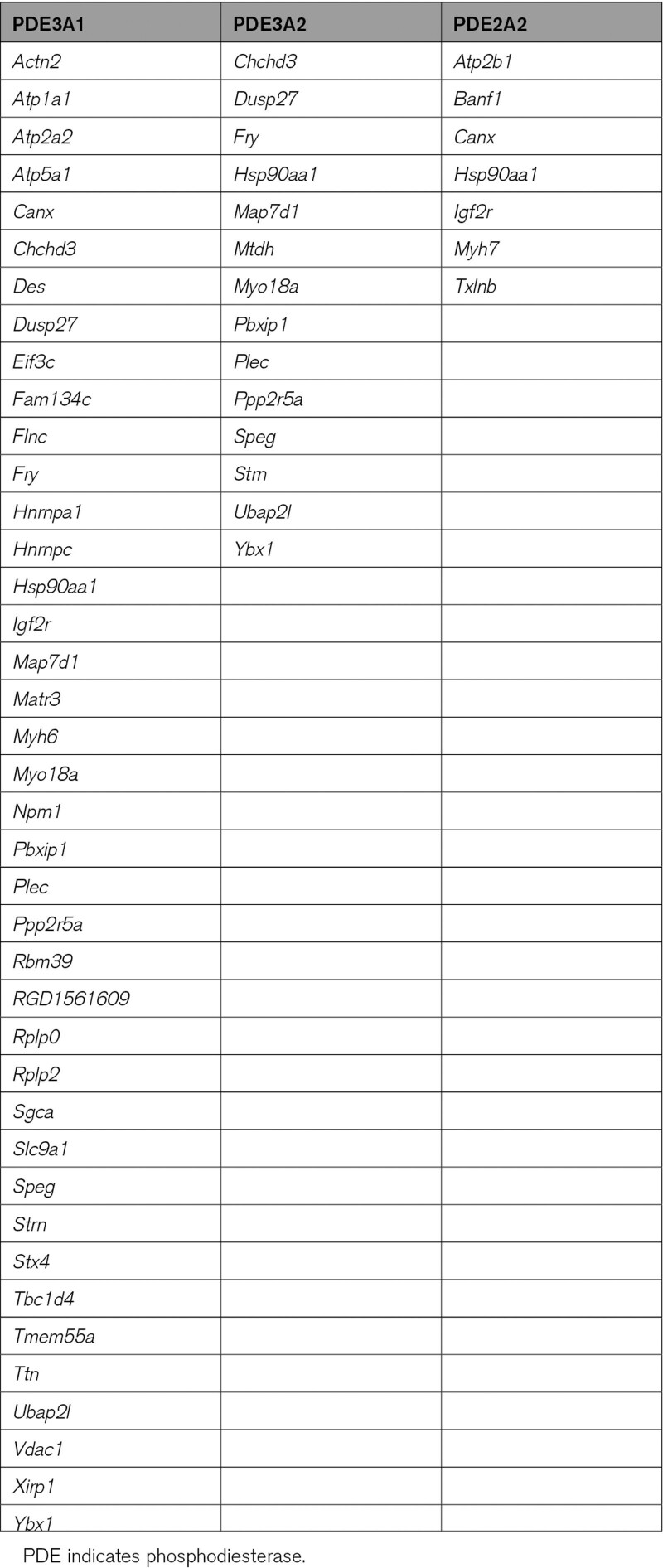
List of Gene Names Encoding for Proteins Identified as Significantly Enriched in Both the Phosphoproteome and Interactomes Data Sets

### PDE3A Isoforms Localize to the Nucleus and Form a Complex With SMAD4 and HDAC-1

**Figure 4.**
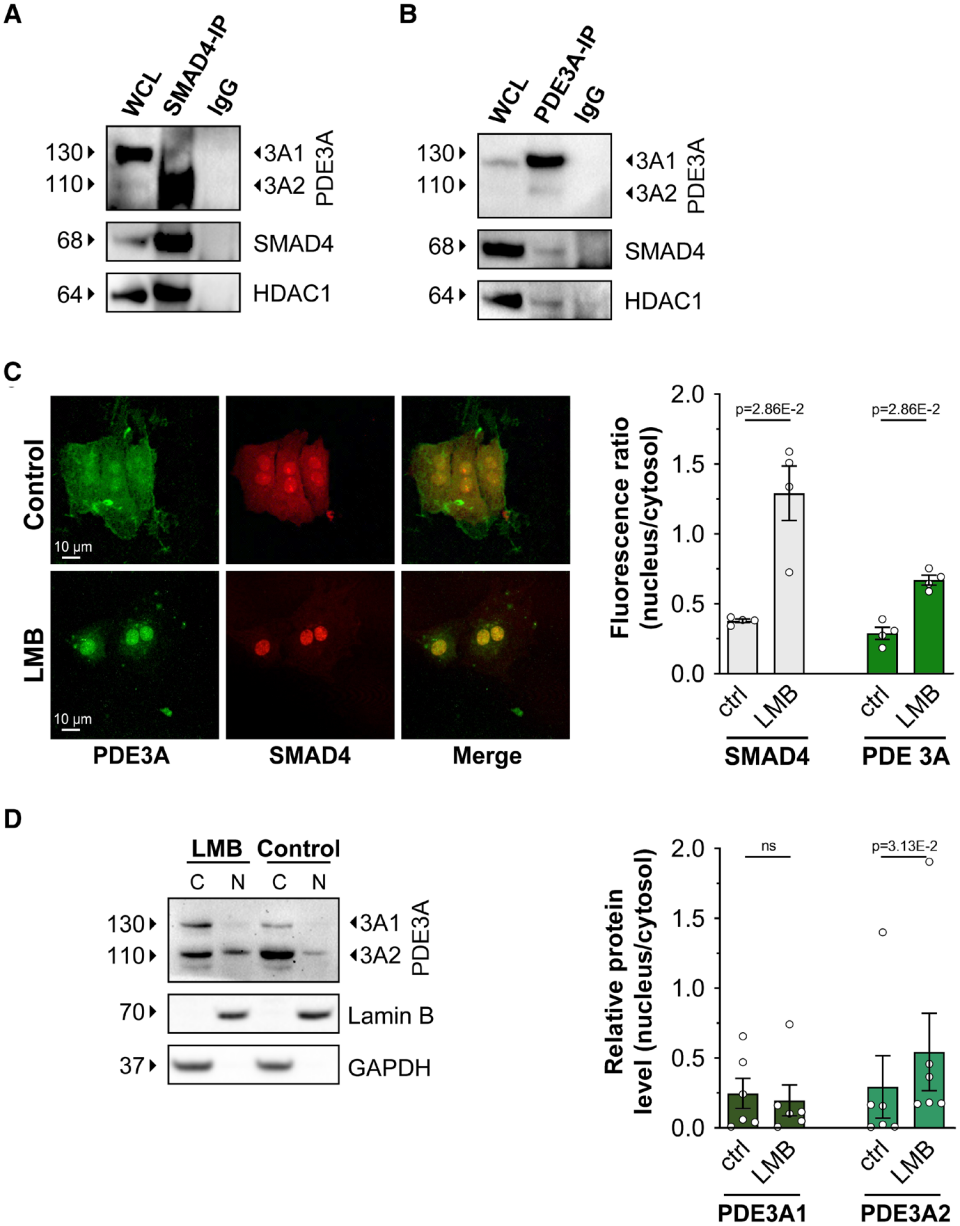
**PDE3A (phosphodiesterase 3A) isoforms are in a nuclear complex involving SMAD4 (SMAD family member 4) and HDAC-1 (histone deacetylase 1). A**, Western blot analysis showing PDE3A2 and HDAC-1 in the pull down of endogenous SMAD4 obtained from neonatal rat ventricular myocyte (NRVM) cell lysates. Representative of n=3 independent cultures. **B**, Detection of endogenous SMAD4 and HDAC-1 in the immunoprecipitate obtained by pulling down endogenous PDE3A from NRVM lysates. n=3. **C**, Immunostaining of endogenous PDE3A and SMAD4 in NRVM cells treated either with DMSO (control) or with leptomycin B (LMB; 100 nmol/L) for 3 hours. Quantification of fluorescence intensity is shown on the **right**. n=4 independent experiments (at least 15 cells per experiment). Mann-Whitney *U* test. **D**, Western blot analysis showing endogenous PDE3A1 and PDE3A2 in the nuclear (N) and cytoplasmic (C) fractions obtained from NRVM treated with 100 nmol/L LMB or dimethyl sulfoxide (DMSO) (control) for 3 hours. Densitometric quantification is shown on the **right**. Values are shown as relative to the cytosolic content and are presented as mean±SEM. n=6 biological replicates. Wilcoxon matched pairs signed-rank test. WCL indicates whole-cell lysate.

We first sought to confirm the interaction between SMAD4 and PDE3A isoforms. Western blot analysis of RFP pulldowns obtained from NRVMs expressing RFP-tagged PDE3A1 or PDE3A2 showed endogenous SMAD4 in the RFP immunoprecipitate (Figure S8A). HDAC-1 was also detected in the same immunoprecipitate (Figure S8A), indicating that PDE3A isoforms, SMAD4 and HDAC-1 can be part of the same complex. To further confirm the interaction between SMAD4 and PDE3A, Flag-tagged SMAD4 was expressed in NRVMs and the Flag immunoprecipitate probed with anti-PDE3A antibody. As shown in Figure S8B, reactivity was detected for endogenous PDE3A2 but not PDE3A1. Interaction of PDE3A isoforms with SMAD4 and HDAC-1 was further confirmed for proteins expressed at endogenous level in NRVM, as shown inFigure 4A and 4B. At the endogenous level, only PDE3A2 appears to be part of the PDE3A/SMAD4/HDAC-1 complex (Figure 4A). Interaction between endogenous PDE3A and SMAD4 is also detectable in rat tissue immunoprecipitates (Figure S8C). Further, we confirmed the interaction between PDE3A2-RFP, endogenous SMAD4, and endogenous HDAC-1 in ARVMs (Figure S8D) and the interaction between PDE3A2-RFP and endogenous SMAD4 in human cardiac myocytes differentiated from hiPSC-CMs (Figure S8E).

Studies in other cell types have shown that while SMAD4 is distributed both in the cytoplasm and in the nucleus,^40^ HDAC-1 is exclusively found in the nucleus,^41^ as we confirmed in NRVM (Figure S9A). Our finding that PDE3A isoforms coimmunoprecipitate with HDAC-1 (Figure 4A and 4B) suggests that at least a fraction of PDE3A enzymes must localize to the nucleus. Analysis of the PDE3A1 and PDE3A2 amino acid sequences using the eukaryotic linear motif prediction tool (http://elm.eu.org/) indeed identified several nuclear localization sequence motifs and a nuclear export sequence known as CRM1 (chromosomal maintenance 1) binding sequence (Figure S9B). Immunostaining of NRVM using anti-PDE3A–specific antibodies confirmed localization of PDE3A to the nucleus that was significantly enhanced upon treatment with leptomycin B—an inhibitor of CRM1-mediated nuclear export (Figure 4C; Figure S9C). Consistently, Western blot analysis of NRVM cytosolic and nuclear fractions showed that, on average, about 30% of total PDE3A2 and 15% of total PDE3A1 localize to the nucleus (Figure 4D)—a fraction that increases to 50% and 19%, respectively, upon leptomycin B treatment (Figure 4D).

### PDE3A Inhibition Affects HDAC-1 Phosphorylation and Activity in a PKA-Dependent Manner

**Figure 5.**
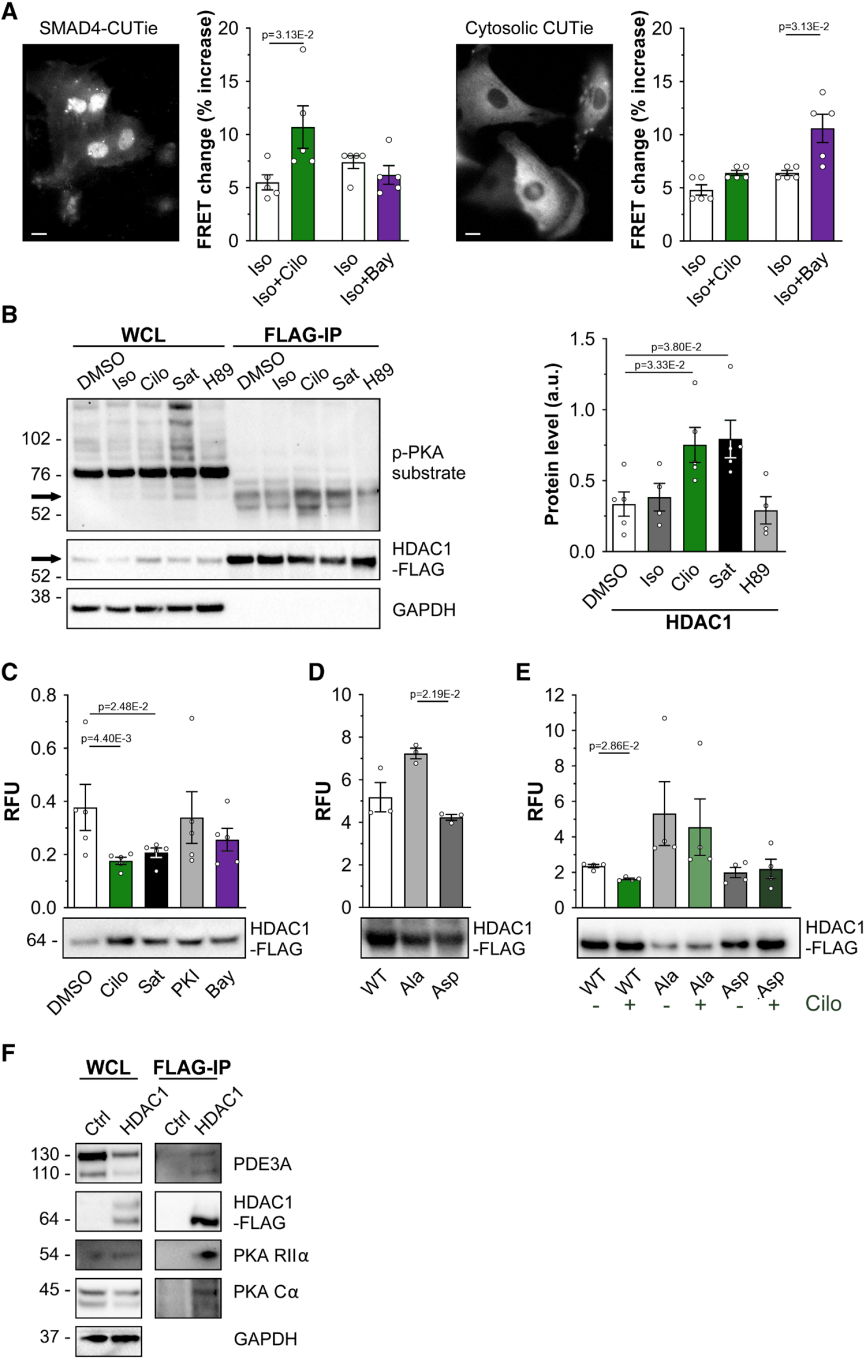
**Inhibition of PDE3A (phosphodiesterase 3A) leads to HDAC-1 (histone deacetylase 1) phosphorylation and inhibition of HDAC-1 deacetylase activity. A**, Representative images showing the distribution of SMAD4 (SMAD family member 4)–cAMP universal tag for imaging experiments (CUTie) and CUTie reporters in neonatal rat ventricular myocytes (NRVMs) treated with 100 nmol/L leptomycin B (LMB) for 3 hours. Summaries of the Förster resonance energy transfer (FRET) change detected with the 2 sensors in NRVM on application of 0.5 nmol/L isoproterenol (ISO) followed by 10 μmol/L cilostamide (Cilo) or 100 nmol/L BAY 60-7550 (BAY) are shown on the **right** and are expressed as percent increase over baseline. n=5 independent NRVM preparations. Wilcoxon matched pairs signed-rank test. **B**, Representative Western blot showing PKA (protein kinase A)-dependent phosphorylation of HDAC-1 (**left**) and densitometric analysis of 5 independent experiments (**right**). HDAC-1-Flag was immunoprecipitated from lysates obtained from NRVM treated with 0.5 nmol/L ISO, 10 μmol/L PDE3 inhibitor Cilo, a saturating cAMP stimulus (Sat, 100 μmol/L 3-isobutyl-1-methylxanthine [IBMX] and 25 μmol/L forskolin), or with 30 μmol/L PKA inhibitor H89. Phosphorylation level was detected with a phospho-PKA substrate antibody. For quantification, values were normalized to the amount of HDAC-Flag in the immunoprecipitates and are presented as mean±SEM. Kruskal-Wallis test with Dunn correction for multiple comparisons. **C**, Deacetylase activity of HDAC-1-Flag immunoprecipitated from NRVMS cells treated for 45 minutes with 10 μmol/L Cilo (Sat, 20 μmol/L cell-permeable PKA inhibitor [PKI]) or 1 μmol/L BAY. HDAC-1 activity is normalized to the relative amount of HDAC-1-Flag present in every sample (as shown in a representative Western blot in the **bottom**) and is presented as mean±SEM. n=5 biological replicates. Kruskal-Wallis test with Dunn correction for multiple comparisons. **D**, Deacetylase activity measured in HDAC-1-Flag pull downs obtained from Human Embryonic Kidney cells 293 (HEK293) expressing wild type (WT) HDAC-1-Flag and the mutants HDAC-1_S406A, S436A_-Flag (Ala) or HDAC-1_S406D, S436D_-Flag (Asp). Quantification as in **C**. n=3 independent experiments. Kruskal-Wallis test with Dunn correction for multiple comparisons. **E**, Deacetylase activity measured in HDAC-1-Flag pull downs obtained from NRVM expressing WT, Ala, or Asp mutants. Quantification as in **C**. n=4 independent experiments. Mann-Whitney *U* test. **F**, Western blot analysis showing PDE3A, PKA regulatory (RIIα) and catalytic (Cα) subunits in the FLAG pull down obtained from lysates of NRVM expressing HDAC-1-Flag. Representative of 3 independent experiments. RFU indicates relative fluorescence unit.

To test whether PDE3A isoforms control cAMP levels in a nuclear nanodomain involving SMAD4 and HDAC-1, we genetically fused the cAMP FRET reporter CUTie^4^ (Figure S10A) to the carboxyl terminus of SMAD4 to generate SMAD4-CUTie (Figure S10B). SMAD4-CUTie showed the expected nuclear localization when expressed in HEK293 cells (Figure S10B) and NRVMs (Figure 5A) and showed kinetics of FRET change comparable to cytosolic CUTie (Figure S10C). FRET imaging of NRVM expressing SMAD4-CUTie showed that, in the presence of isoproterenol, inhibition of PDE3 significantly increased cAMP in the immediate proximity of nuclear SMAD4, whereas inhibition of PDE2A had no effect (Figure 5A); this is in contrast to the bulk cytosol, where PDE2A inhibition led to a significantly larger increase in cAMP than inhibition of PDE3 (Figure 5A).

PKA is known to phosphorylate HDAC-1,^41^ but the functional effect has not been investigated. To examine whether inhibition of PDE3 results in increased phosphorylation of HDAC-1, NRVMs expressing Flag-tagged HDAC-1 were treated with isoproterenol (0.5 nmol/L), cilostamide (10 μmol/L), or a saturating stimulus (25 μmol/L forskolin and 100 μmol/L IBMX). Analysis of Flag-HDAC-1 pulldowns using a PKA phospho-substrate antibody confirmed a significant increase in HDAC-1 phosphorylation upon inhibition of PDE3, similar to the phosphorylation level achieved at saturating cAMP (Figure 5B). These findings were confirmed in ARVMs (Figure S11A) and in hiPSC-CMs (Figure S11B). The phosphorylation of HDAC-1 on inhibition of PDE3 was completely abolished by inhibition of PKA with PKA inhibitor (PKI) (Figure S11A and S11B). Consistently, overexpression of PDE3A isoforms in HEK293 cells drastically reduced PKA-dependent HDAC-1 phosphorylation (Figure S11C).

We next assessed the effect of PDE3 inhibition on HDAC-1 activity. As shown in Figure 5C, the deacetylase activity measured in HDAC-1-Flag pulldowns obtained from NRVM treated with cilostamide was significantly reduced compared with DMSO control—an effect that was not recapitulated by inhibition of PDE2A with BAY (Figure 5C). In line with these findings, an HDAC-1 phospho-mimic mutant, where the PKA phosphorylation sites are mutated to aspartate (HDAC-1_S406D, S436D_), showed significantly decreased deacetylase activity compared with a phospho-null HDAC-1 mutant, where the same residues are mutated to alanine (HDAC-1_S406A, S436A_; Figure 5D). Consistently, while inhibition of PDE3 significantly reduced WT (wild type) HDAC-1 activity, cilostamide treatment had no effect on the activity of the phospho-mimic or phospho-null HDAC-1 mutants (Figure 5E).

If the PDE3A2/SMAD4/HDAC-1 complex constitutes a nuclear nanodomain where HDAC-1 activity is regulated by a local cAMP pool, PKA is expected to be present within the same complex. Immunoprecipitation experiments using cell lysates from NRVM expressing Flag-HDAC-1 demonstrate that, in addition to PDE3A, both catalytic and regulatory subunits of PKA are present in the immunoprecipitate (Figure 5F). Notably, when we used for this experiment cell lysates from HEK293 cells, which do not express detectable levels of PDE3A endogenously,^10^ the regulatory subunit of PKA, but no catalytic subunit, was detected in the HDAC-1 immunoprecipitate (Figure S12). By contrast, both regulatory and catalytic subunits of PKA were present in the immunoprecipitate obtained from HEK293 cells overexpressing PDE3A1 or PDE3A2 (Figure S12), confirming that PDE3A is required at the SMAD4/HDAC-1 nuclear complex to degrade locally cAMP and maintain PKA within the complex in its inactive holoenzyme conformation.

### PDE3A Inhibition Regulates GATA4 Expression

**Figure 6.**
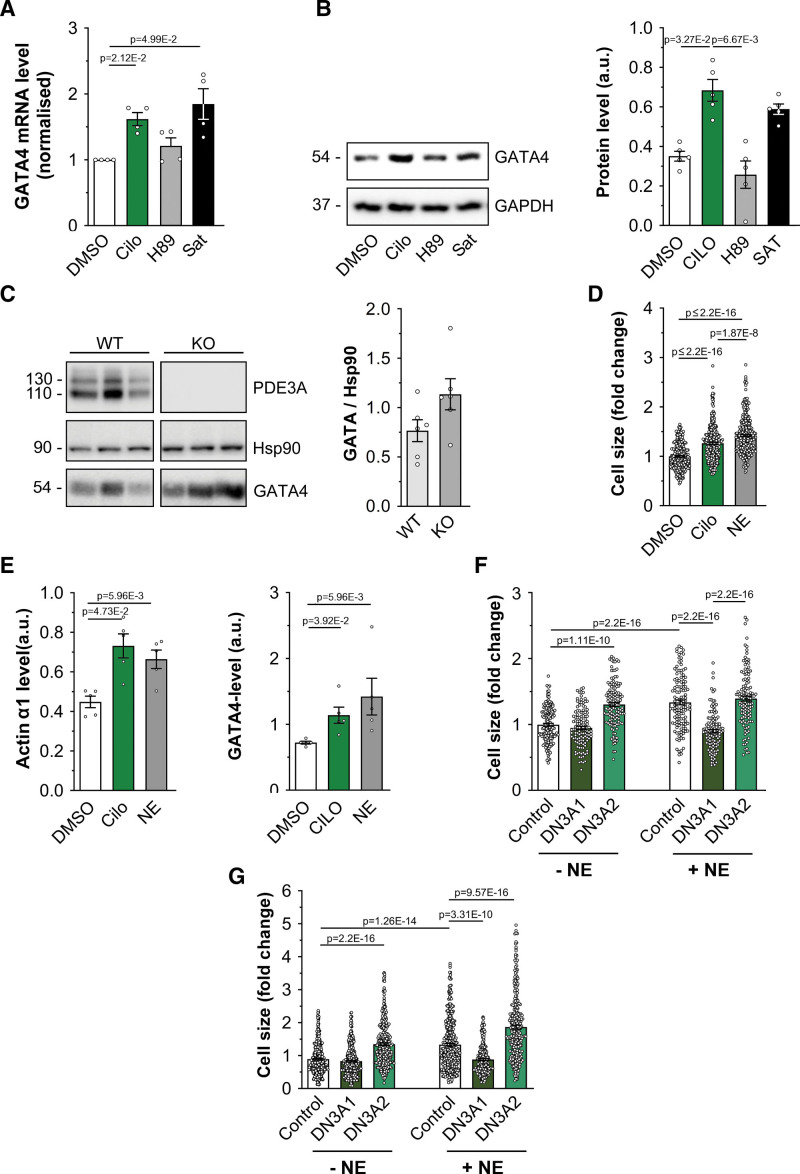
**Inhibition of PDE3A (phosphodiesterase 3A) regulates GATA4 (GATA4 binding protein 4) expression. A**, Quantitative real-time polymerase chain reaction analysis of GATA4 mRNA transcripts in neonatal rat ventricular myocyte (NRVM) cells treated with 10 μM cilostamide (Cilo), saturating treatment (Sat, 100 μmol/L 3-isobutyl-1-methylxanthine [IBMX] and 25 μmol/L forskolin) or 30 μmol/L H89 for 2.5 hours. Bars show GATA4 mRNA values normalized to GAPDH and dimethyl sulfoxide (DMSO) treatment and are presented as mean±SEM. n=4 biological replicates. Kruskal-Wallis test with Dunn correction for multiple comparisons. **B**, Western blot analysis and quantification of GATA4 protein expression in NRVM cells treated with Cilo (10 μmol/L), H89 (30 μmol/L), or saturating treatment. GAPDH served as a loading control. Values are normalized to GAPDH and are presented as mean±SEM. n=5 biological replicates. Kruskal-Wallis test with Dunn correction for multiple comparisons. **C**, Western blot analysis and quantification of endogenous GATA4 expression in cardiac tissue lysates obtained from PDE3A knockout or wild-type control rats. Hsp90 served as a loading control and for normalization in the quantification. Values are mean±SEM. n=6 biological replicates. Mann-Whitney *U* test. **D**, Cell size measured in adult rat ventricular myocytes (ARVMs) either untreated or treated with Cilo (10 mmol/L) or NE (10 μmol/L) for 24 hours. Values are normalized to DMSO control and expressed as mean±SEM. n=5 independent experiments (at least 241 cells per condition). Normality of log-transformed data was tested with Anderson-Darling test. *P* values were determined by a hierarchical significance test using log-transformed data followed by Bonferroni correction. **E**, Quantification by Western blot analysis of hypertrophy markers protein expression level in samples as shown in **D**. n=5 independent experiments. Kruskal-Wallis test with Dunn's correction. **F**, Cell size measured in ARVM expressing mCherry (control) or the catalytically inactive mutant PDE3A1-DN-RFP or PDE3A2-DN-RFP (**left**) and treated as indicated for 24 hours. n=5 independent experiments (at least 53 cells per condition). Normality was established by the Anderson-Darling test. Hierarchical significance analysis with final Bonferroni correction for multiple comparisons. **G**, Cell size measured in human induced pluripotent stem cell-derived cardiomyocytes (hiPSC-CM) expressing mCherry (control) or the catalytically inactive mutant PDE3A1-DN-RFP or PDE3A2-DN-RFP (**left**) and treated as indicated for 48 hours. n=6 independent experiments (6 different differentiations of 3 independent hiPSC lines, at least 231 cells per condition). *P* values were determined by hierarchical significance test using log-transformed data followed by Bonferroni correction.

To establish whether inhibition of PDE3A affects gene transcription, we treated NRVM with cilostamide and measured the expression level of the transcription factor GATA4, as this is known to be regulated by SMAD4^42,43^ and HDAC-1.^44^ We found that PDE3 inhibition resulted in upregulation of GATA4 expression, both at the mRNA (Figure 6A) and protein (Figure 6B) levels. Consistently, GATA4 expression was upregulated in heart tissue obtained both from mice where the *PDE3A* gene was ablated by homologous recombination^45^ (Figure S13A) and from rats carrying a homozygous frameshift mutation (Δ20 bp) in the *PDE3A* gene that results in a functional deletion phenotype^10^ (Figure 6C).

GATA4 is upregulated in response to hypertrophic stimuli, including pressure overload and β-adrenergic activation.^42^ We, therefore, assessed whether inhibition of PDE3 may regulate cardiac myocyte hypertrophic growth in an in vitro model of cardiac hypertrophy. We found that treatment with cilostamide was sufficient to significantly increase cardiac myocyte cell size in NRVM (Figure S13B). The prohypertrophic effect of PDE3 inhibition was also confirmed in ARVM (Figure 6D and 6E) and hiPSC-CM (Figure S13C and S13D). Consistent with the observation that PDE3A isoforms control gene expression via interaction with SMAD4 and regulation of a local nuclear pool of cAMP, overexpression of catalytically inactive mutants of PDE3A1 and PDE3A2 (PDE3A1-DN and PDE3A2-DN), which exert a dominant negative effect by displacing the endogenous active counterpart from interacting partners,^46^ resulted in significantly higher GATA4 levels compared with expression of the WT enzymes (Figure S13E). Accordingly, PDE3A2-DN was sufficient, per se, to induce hypertrophic growth when expressed in NRVM (Figure S14, as well as in ARVM (Figure 6F) and hiPSC-CM (Figure 6G). Unexpectedly, expression of PDE3A1-DN showed no effect on the size of otherwise untreated cardiac myocytes (Figure S14; Figure 6F and 6G) while it completely reversed the hypertrophy induced by norepinephrine—a well-established prohypertrophic stimulus (Figure S14; Figure 6F and 6G). These findings suggest that displacement of PDE3A1 from locations distinct from the SMAD4/HDAC-1 complex overrides the effect of any displacement of PDE3A from the nuclear SMAD4/HDAC-1 nanodomain and triggers instead a response that inhibits norepinephrine-induced hypertrophic growth.

### PDE3 Inhibition Leads to Hypertrophy via Regulation of HDAC-1 Phosphorylation

**Figure 7.**
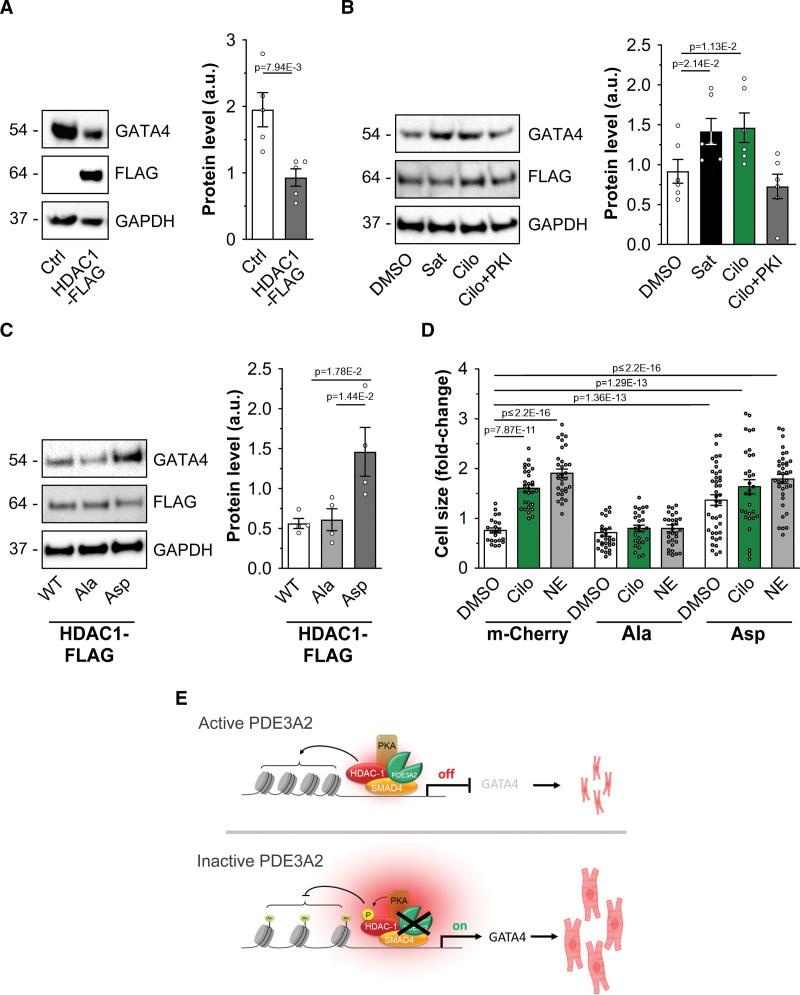
**Inhibition of PDE3A (phosphodiesterase 3A) affects cardiac myocyte hypertrophic growth. A**, Western blot analysis and densitometric quantification of GATA4 (GATA binding protein 4) expression in cells overexpressing HDAC-1 (histone deacetylase 1)-Flag. Values are normalized to GAPDH and presented as mean±SEM. n=5 biological replicates. Mann-Whitney *U* test. **B**, Western blot analysis and quantification showing expression of GATA4 in neonatal rat ventricular myocyte (NRVM) cells overexpressing HDAC-1-Flag and treated with cilostamide (Cilo; 10 μmol/L), saturating cAMP (Sat, 100 μmol/L 3-isobutyl-1-methylxanthine [IBMX] and 25 μmol/L forskolin) of PKA inhibitor (PKI; 20 μmol/L). n=6 biological replicates. Values are normalized to GAPDH and to Flag signal and are presented as mean±SEM. Friedman test and Dunn correction for multiple comparisons. **C**, Western blot analysis and quantification showing expression of GATA4 in NRVM cells overexpressing HDAC-1-Flag (wild type [WT]), the phospho-null mutant HDAC-1_S406A-S436A_-Flag (Ala), or the phospho-mimic mutant HDAC-1_S406D-S436D_-Flag (Asp). GAPDH was used as a loading control. For quantification, values were normalized to GAPDH and Flag signal and are expressed as means±SEM. n=5 independent experiments. Kruskal-Wallis test with Dunn's correction for multiple comparisons. **D**, Cell size measured in NRVM expressing mCherry, HDAC-1_S406A-S436A_-Flag (S>A), or HDAC-1_S406D-S436D_-Flag (S>D) and treated with DMSO, Cilo (10 μmol/L), or NE (10 μmol/L) for 48 hours. Values are expressed as fold change relative to untransfected and dimethyl sulfoxide (DMSO) treated within the same transfection group and are presented as mean±SEM. n=6 independent experiments (6 different differentiations from 3 independent human induced pluripotent stem cell lines, at least 27 cells per condition). Anderson-Darling log normality test and hierarchical analysis of log-transformed data followed by Bonferroni correction. **E**, Schematic illustration of the PDE3A/SMAD4 (SMAD family member 4)/HDAC-1 nuclear ND. **Top**, With active PDE3A2 at the SMAD4/HDAC-1 nuclear complex cAMP levels are locally low and PKA (protein kinase A) is inactive. HDAC-1 deacetylates histones, repressing expression of prohypertrophic genes. **Bottom**, Inhibition of PDE3A or displacement of PDE3A2 results in a local increase in cAMP, activation of local PKA, and phosphorylation of HDAC-1, leading to inhibition of its deacetylase activity. As a result, transcription of prohypertrophic genes is enhanced, leading to cardiac myocyte hypertrophy. Some elements used in **E** have been generated using BioRender.com.

Histone deacetylation by HDACs is generally associated with repression of gene expression.^47^ Consistently, HDAC-1 overexpression in NRVM resulted in decreased GATA4 expression (Figure 7A). This effect was counteracted by inhibition of PDE3A in a PKA-dependent fashion (Figure 7B). Further confirming that PKA-dependent phosphorylation inhibits HDAC-1 activity with impact on gene transcription, NRVM expressing the phospho-mimic HDAC-1_S406D, S436D_ mutant showed significantly higher GATA4 expression compared with cells expressing the phospho-null HDAC-1_S406A, S436A_ mutant (Figure 7C). Notably, overexpression of the phospho-null HDAC-1_S406A, S436A_ mutant completely blocked the effect of cilostamide or norepinephrine on hypertrophic growth, whereas overexpression of the phospho-mimic HDAC-1_S406D, S436D_ mutant was sufficient per se to induce hypertrophy, to a similar degree as cilostamide or norepinephrine (Figure 7D), confirming that the effect of PDE3A inhibition on cardiac myocyte size relies on the PKA-dependent phosphorylation of HDAC-1.

## Discussion

Cardiac myocytes express >100 different GPCRs (G-protein–coupled receptors), many of which regulate cell function primarily via the generation of cAMP.^48^ Specificity of response is achieved via subcellular compartmentalization where PDEs play a key role by differentially hydrolyzing cAMP and generating multiple nanodomains with different cAMP concentration. The functional relevance of cAMP compartmentalization is illustrated in the heart by the ability of cAMP to relay sympathetic signals with exacting precision and no interference from the myriad of other cAMP-modulated processes that are independent of, but run concurrently with, β-adrenergic regulation of cardiac myocyte contraction and relaxation.^49,50^ Spillage of information among these parallel pathways is avoided through coordinated spatial and temporal regulation of the multiple coexisting cAMP nanodomains.

Only a limited number of cardiac cAMP nanodomains, which primarily involve some of the prototypical cardiac targets of β-adrenergic stimulation, have been characterized so far, leaving the global landscape of subcellular cAMP nanodomain largely undefined. Genetically encoded fluorescent reporters have recently provided unequivocal evidence that the size of subcellular cAMP nanodomains is below the resolution of optical microscopy.^4–6^ Visualization of cAMP domains, therefore, relies on the ability to target the cAMP probe within the domain itself,^4,8,18^ making identification and characterization of novel cAMP nanodomains difficult when previous knowledge of their subcellular location is not available.

To overcome this limitation, and with the view that individual PDEs can serve as signposts to denote nanodomains with distinct regulation of cAMP, we adopted a proteomics approach that integrates the analysis of the interactome of a specific PDE isoform with the analysis of the phosphoproteome associated with inhibition of that PDE to identify previously unknown cAMP nanodomains associated with sympathetic signaling. We focused on PDE3A-regulated nanodomains, anticipating that knowledge of where these domains are located may help identify pathways involved in the long-term adverse cardiac effects observed in patients with HF receiving PDE3 inhibitors. For comparison, we investigated the nanodomains associated with PDE2A2, the inhibition of which has been associated with cardioprotective effects.^46,51^ Our analysis demonstrates that these PDEs operate largely in distinct subcellular locations and, therefore, define sites undergoing unique regulation of cAMP. In addition to providing information on the subcellular location, this strategy has the advantage of revealing molecular cues that can be useful to inform further mechanistic studies to establish the function associated with specific nanodomains. In addition, by integrating information on the identity of PDE binding partners with information on the identity of PDE phosphorylation targets, this approach reveals nonobvious interactions that could go unnoticed or be neglected. Extended to other PDE isoforms, this strategy can define the full topography of cAMP nanodomains associated with β-adrenergic stimulation in cardiac myocytes. A similar approach can be applied to dissect the cAMP nanodomain landscape associated with other GPCRs and ligands and to establish how cAMP nanodomains are affected in pathological conditions. Given the ubiquitous role of cAMP signaling in all organs and systems, the same strategy is applicable to dissect local cAMP signaling in other cell types.

Our investigation yielded a set of individual proteins that are simultaneously present in the interactome and in the phosphoproteome associated specifically with either PDE3A or PDE2A, strongly indicating that these proteins pinpoint specific cAMP nanodomains; validation of these nanodomains is currently undergoing. To capture additional PDE3A-dependent cAMP nanodomains, we exploited GOCC annotation and found that the term nucleus was highly enriched in both PDE3A1 and PDE3A2 interactomes and in the cilostamide-associated phosphoproteome. Among the nucleus-annotated proteins, we selected the CtBP2, phospho-HDAC-1, and SMAD4 interaction for further analysis as these proteins form a complex that acts downstream of TGF-β (transcription growth factor beta).^52–55^ Notably, TGF-β is associated with cardiac hypertrophy, fibrosis, and conduction abnormalities,^43,56^ all features that characterize the long-term detrimental effect that PDE3 inhibitors have in patients with HF. Collectively, our results demonstrate the presence of a cAMP nanodomain localized to the nucleus of cardiac myocytes where PDE3A2 forms a complex with SMAD4 and HDAC-1. The local increase in cAMP elicited by PDE3 inhibition enhances PKA-dependent phosphorylation of HDAC-1 and inhibition of its deacetylase activity. This results in derepression of prohypertrophic gene transcription and cardiac myocyte hypertrophy (Figure 7C). Previous studies suggest that enhanced apoptosis mediated by ICER (inducible cAMP early repressor) contributes to the adverse effects of PDE3 inhibition.^57^ Although ICER does not appear in the PDE3A interactomes identified in this study, HDAC-1 recruitment has been associated with ICER repressor activity.^58^ It is, therefore, possible that a nuclear nanodomain that includes HDAC-1 controls both the prohypertrophic and proapoptotic effects of PDE3 inhibition and that both these effects participate in the progression of adverse cardiac remodeling. Our observation that the PDE3A2/SMAD4/HDAC-1 nanodomain also operates in human cardiac myocytes differentiated from 4 independent pluripotent stem cell lines indicates that this pathway may contribute to the negative long-term clinical outcome observed in patients with HF receiving PDE3 inhibitors. In support of the relevance of the nuclear PDE3A2/SMAD4/HDAC-1 nanodomain in vivo, a recent characterization of the PDE3A functional deletion rat model^10^ shows that, upon chronic β-AR challenge, the same degree of cardiac remodeling is observed in knockout and in WT rats, despite the knockout animals displaying significantly lower blood pressure (Figure S15),^59^ indicating that, in vivo, loss of PDE3A sensitizes the animals to cardiac stress.

The findings presented here provide an explanation for the apparently conflicting data on the role of PDE3A in heart disease, where both elevation^60^ and reduction^57^ of PDE3A expression and activity have been associated with HF and both ablation^60^ and overexpression^57^ of PDE3A isoforms have been reported to provide cardioprotective effects. Most studies so far have not investigated the role of specific PDE3A isoforms in cardiac disease, and it will be interesting to explore in the future whether the changes in the level of PDE3A expression associated with HF involve individual isoforms. Cardiac overexpression of PDE3A1 has been reported to associate with cardiac remodeling but also to confer cardioprotection to ischemia-reperfusion.^61^ Our results indicate that these opposing effects could be mediated by PDE3A1 regulating cAMP at multiple nanodomains. Our findings also indicate that overexpression may force interactions that, although possible, do not occur when the protein is expressed at an endogenous level. PDE3A1 and PDE3A2 are expressed from the same gene from alternative start sites, and their amino acid sequence is identical except for a unique N-terminal domain in PDE3A1.^62^ We find that, when overexpressed, PDE3A1 can interact with SMAD4/HDAC-1, suggesting that this interaction involves the conserved C-terminal sequence shared by PDE3A1 and PDE3A2. Consistently, overexpression of PDE3A1-DN, which only differs from the WT counterpart by 2 amino acid substitutions in the catalytic site, can displace endogenous PDE3A2 from the SMAD4/HDAC-1 complex and enhance GATA4 expression (Figure S13E). However, when expressed at endogenous levels, PDE3A1 is not associated with SMAD4/HDAC-1. Our findings show that endogenous PDE3A1 controls cAMP at sites distinct from the SMAD4/HDAC-1 nuclear complex and that ≥1 of these sites are associated with antihypertrophic effects. Displacement of PDE3A1 from those locations has a dominant effect over displacement of PDE3A2 from the SMAD4/HDAC-1 domain, as demonstrated by the complete reversal of hypertrophy we observe on overexpression of PDE3A1-DN. By contrast, overexpression of PDE3A2-DN does not displace endogenous PDE3A1 from the locations that protect from hypertrophy. This suggests that anchoring of PDE3A1 at those extranuclear sites involves its unique N-terminal domain. Further studies will be necessary to identify the location associated with the protective effect of PDE3A1 displacement, and the PDE3A1-selective nanodomains identified in this study by our integrated proteomics analysis provides a list of candidates for these investigations. The identification of these sites may open the possibility to selectively modulate cAMP at those sites, with potential therapeutic applications.

Recent studies have challenged the classic paradigm that PKA-dependent regulation of gene expression relies on cytosolic activation of PKA and subsequent diffusion of active catalytic subunits to the nucleus by providing evidence of a nuclear subset of PKA holoenzyme^63,64^ that responds to a nuclear pool of cAMP,^65^ although proof that this nuclear cAMP/PKA domain regulates gene transcription has been lacking. Our work significantly expands our understanding of PKA signaling and regulation by providing direct evidence that a nuclear cAMP/PKA domain under the control of PDE3A2 controls expression of prohypertrophic genes. Notably, our data show that the PDE3A activity at the SMAD4/HDAC-1 nanodomain is sufficient to prevent phosphorylation of HDAC-1 in response to subnanomolar concentrations of isoproterenol (Figure 5B; Figure S11B). This indicates that PDE3A2 at this site provides a local shielding mechanism that protects cardiac myocytes from hypertrophic growth when β-adrenergic stimulation executes the physiological control of cardiac contraction and relaxation.

## Article Information

### Acknowledgments

PDE3A (phosphodiesterase 3A) knockout mouse heart tissue was a kind gift from Dr Joel Moss (National Heart, Lung, and Blood Institute, Bethesda) and Dr Alex KleinJan (Erasmus MC, Rotterdam). The M180 and M398 human induced pluripotent stem cell lines were a kind gift from Dr Christian Pinset (Center for the Study of Stem Cells/Institute for Stem cell Therapy and Exploration of Monogenic diseases, Corbeil Essonnes).

### Author Contributions

G. Subramaniam, K. Schleicher, D. Kovanich, and M. Zaccolo designed the experiments. G. Subramaniam, K. Schleicher, D. Kovanich, A. Zerio, M. Folkmanaite, N.C. Surdo, M. Ercu, A. Koschinski, V. Meraviglia, Y.-C. Chao, A. Sholokh, and K. Chan Park performed the experiments and analyzed data. A. Koschinski, A. Sholokh, A.J.R. Heck, E. Klussmann, M. Bellin, S. Zanivan, S. Hester, S. Mohammed, J. Hu, and M. Zaccolo analyzed data; G. Subramaniam, K. Schleicher, and M. Zaccolo wrote the manuscript. M. Zaccolo conceived the research question and oversaw the entirety of research.

### Sources of Funding

This study was supported by grants from the British Heart Foundation (BHF; PG/15/5/31110 and RG/17/6/32944) and the BHF Centre of Research Excellence, Oxford (RE/13/1/30181, RE/18/3/34214, and RE/18/3/34214) and the Oxford NIHR Biomedical Research Centre to M. Zaccolo; the Horizon 2020 project Epic-XS (823839) to A.J.R. Heck; the Deutsche Forschungsgemeinschaft (program project grant, 394046635–SFB 1365) and the German Israeli Foundation (I-1452-203/13-2018) to E. Klussmann; the Netherlands Organisation for Health Research and Development ZonMW (MKMD project No. 114022504) and the European Research Council (ERC-CoG 101001746 Mini-HEART) to M. Bellin; and the Stand Up to Cancer campaign for Cancer Research UK grant A29800 to S. Zanivan.

### Disclosures

None.

## Supplementary Material
