# Supplementary material for: Integrated Proteomics Unveils Nuclear PDE3A2 as a Regulator of Cardiac Myocyte Hypertrophy
Source: Circ Res. 2023 Mar 8;132(7):828–48. doi: 10.1161/CIRCRESAHA.122.321448 (PMC10045983; doi:10.1161/CIRCRESAHA.122.321448)
Supplement: Supplementary file 5 [file res-132-828-s005.pdf]

Supplementary Figure 1

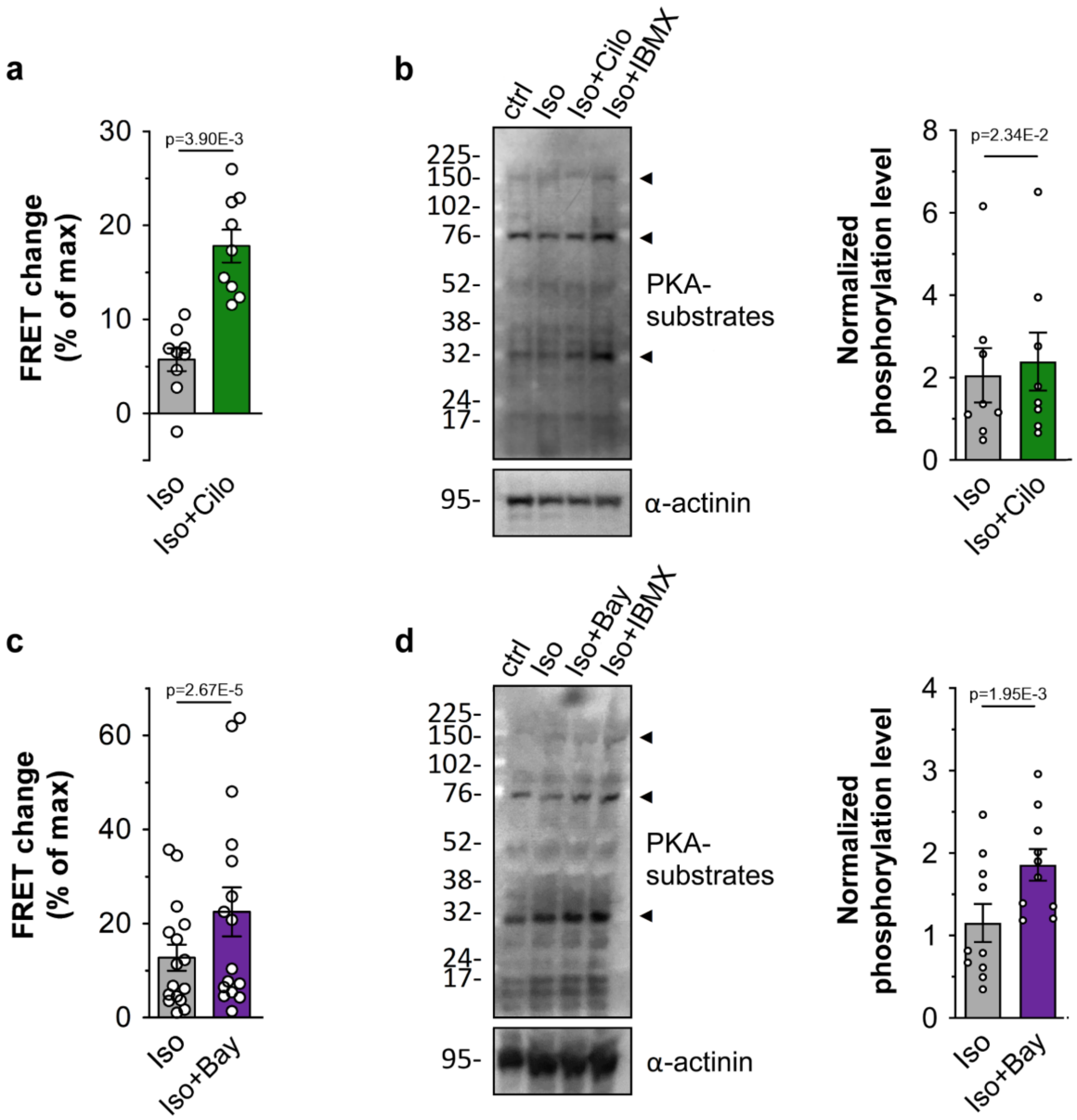

Supplementary Figure 1 | Effect of *PDE3 or PDE2A inhibition on cytosolic cAMP and PKA-dependent phosphorylation in cardiomyocytes under β-adrenergic stimulation. (a)* Quantification of cAMP increase in ARVMs expressing the cytosolic FRET reporter EPAC-S^{H187} and treated with 0.5nmol/L ISO and subsequently with the PDE3 inhibitor Cilo (10µmol/L). Responses are calculated as increase over baseline and expressed as relative to sensor saturation achieved by treatment with 25µmol/L forskolin and 100µmol/L IBMX at the end of the experiment. n=10. Wilcoxon Signed Rank Test paired test. (**b**) Representative immunoblot of lysates obtained from ARVMs treated with 0.5nmol/L ISO alone or in combination with either 10µmol/L Cilo or 100µmol/L IBMX and probed with total PKA substrate antibody. Quantification from n=7 independent experiments is shown on the right. Wilcoxon Signed Rank Test . (**c**) Quantification of cAMP increase in ARVMs expressing EPAC-S^{H187} and treated with 0.5nmol/L ISO and subsequently with the PDE2 inhibitor Bay (1µmol/L). Responses calculated as in (a). n=9. Paired Studen's t-test. (**d**) Representative immunoblot of lysates from ARVMs treated with 0.5nM ISO alone or in combination with either 1µmol/L BAY or 100µmol/L IBMX and probed with total PKA substrate antibody. Quantification from n=10 independent experiments is shown on the right. Wilcoxon Signed Rank Test paired test. For (b) and (d) Ctrl indicates untreated cells. α-actinin was used as a loading control. Arrowheads indicate bands selected for quantification. For all experiments bars indicate mean values ± s.e.m.

Supplementary Figure 2

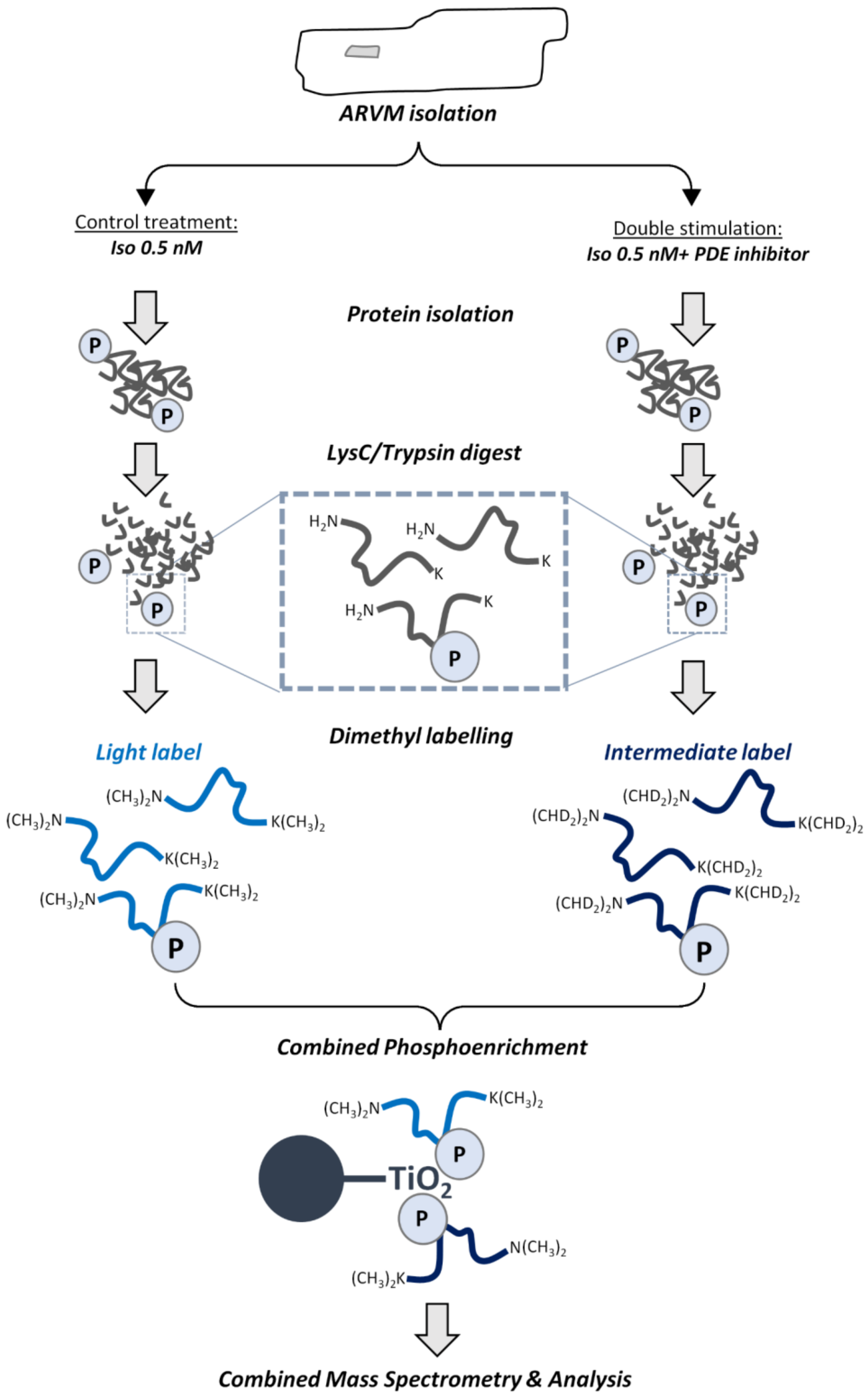

Supplementary Figure 2 | Experimental workflow for the acquisition of PDE3- and PDE2A-dependent phosphoproteomes in ARVM using quantitative phosphoproteomics with stable isotope dimethyl labelling.

Supplementary Figure 3

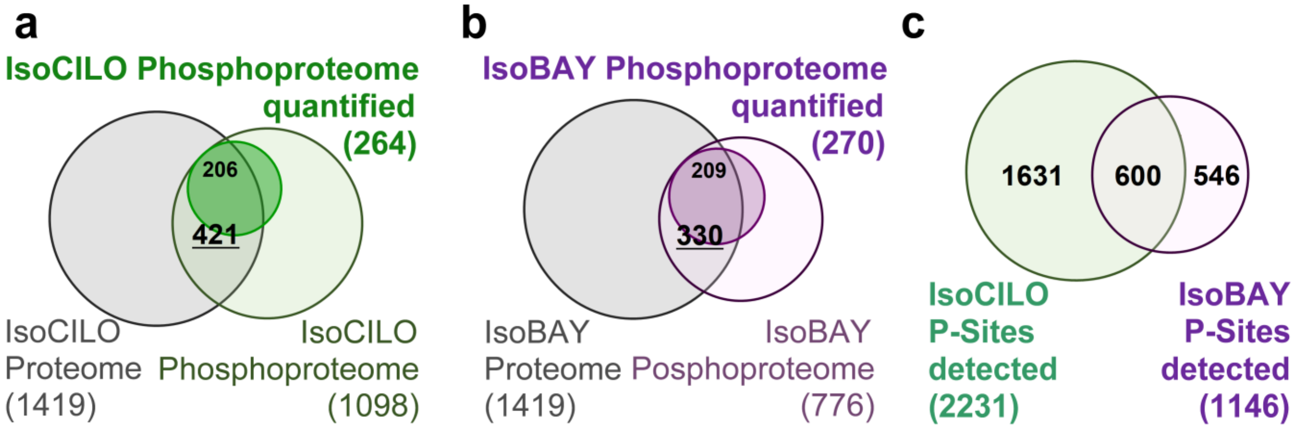

Supplementary Figure 3 | Area-proportional Venn diagram to compare protein identification before and after phospho-enrichment in samples treated with the PDE3 (**a**) or the PDE2A (**b**) inhibitor. (**c**) Venn diagram to compare overall phospho-site identification in the sample treated with PDE3 inhibitor (green) or PDE2A inhibitor (purple).

Supplementary Figure 4

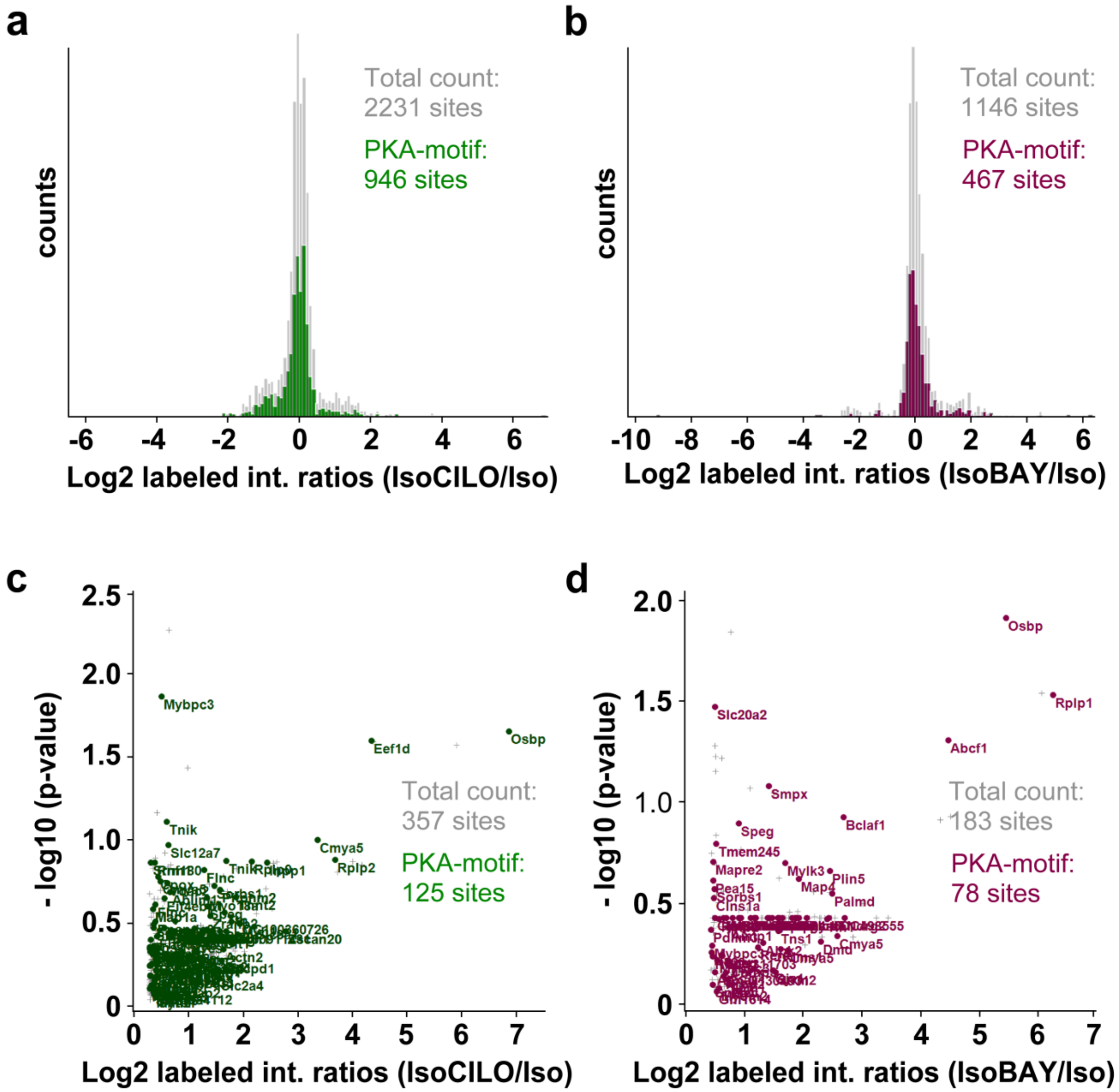

Supplementary Figure 4 | *PKA motif in the PDE3 and PDE2A-dependent phospho-proteomes.* (**a**) Abundance of the PKA motif in the cardiac phospho-proteome after treatment with 0.5nM ISO alone or in combination with 10µM CILO. 946 phospho-peptides with a bioinformatically annotated PKA motif are highlighted in green. (**b**) Abundance of the PKA motif in the cardiac phospho-proteome after treatment with 0.5nM ISO alone or in combination with 1µM BAY. 467 phospho-peptides with a bioinformatically annotated PKA motif are highlighted in purple. Scatter plot of phosphopeptides within the top 16% intensity ratios values for the sample treated with CILO (**c**) or BAY (**d**) versus control. Peptides with an annotated PKA motif are highlighted in green and purple, respectively, with the corresponding protein name. All other peptides are indicated with a grey cross.

Supplementary Figure 5

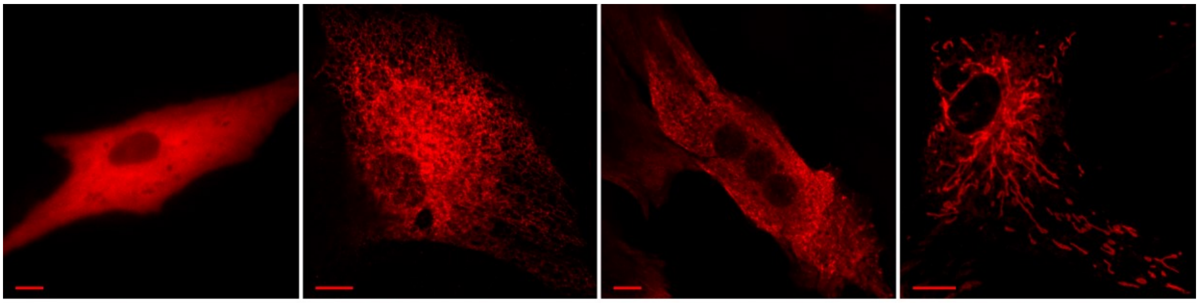

Supplementary Figure 5 | Subcellular localisation of PDE3A1, PDE3A2 and PDE2A2. Representative images showing the localisation pattern of mCherry (control), PDE3A1-mCherry, PDE3A2-mCherry and PDE2A2-mCherry in neonatal rat ventricular myocytes (NRVM). Bar size = 10 μm

Supplementary Figure 6

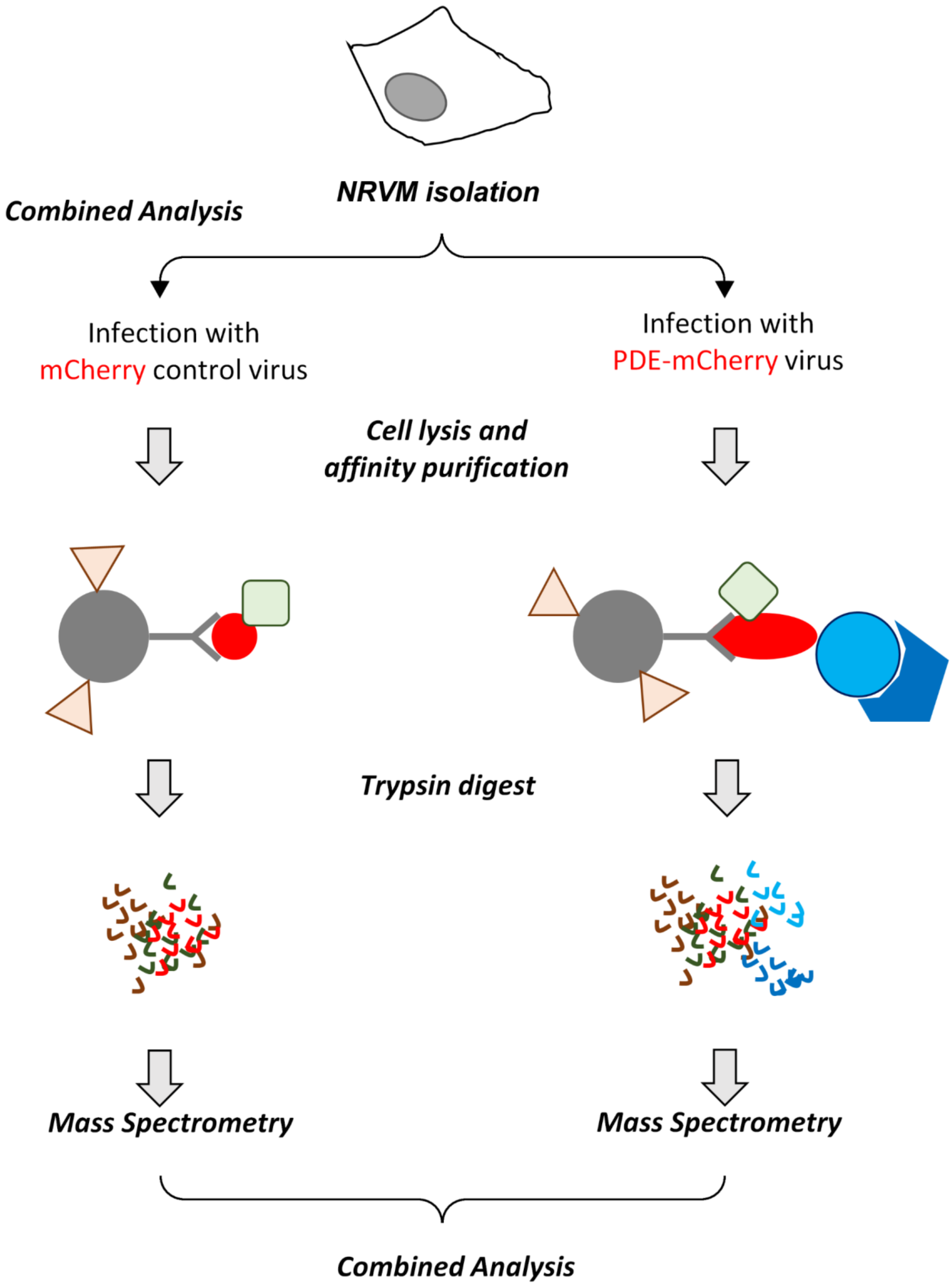

Supplementary Figure 6 | Experimental workflow for the analysis of the PDE3A1, PDE3A2 and PDE2A2 interactomes in neonatal rat ventricular myocytes (NRVM) using label-free proteomics.

Supplementary Figure 7

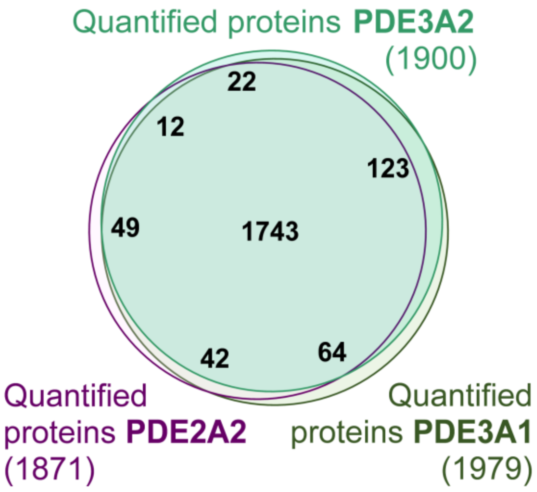

Supplementary Figure 7 | Area-proportional Venn diagram to compare overall protein quantification in PDE3A1, PDE3A2 and PDE2A2 affinity purifications.

Supplementary Figure 8

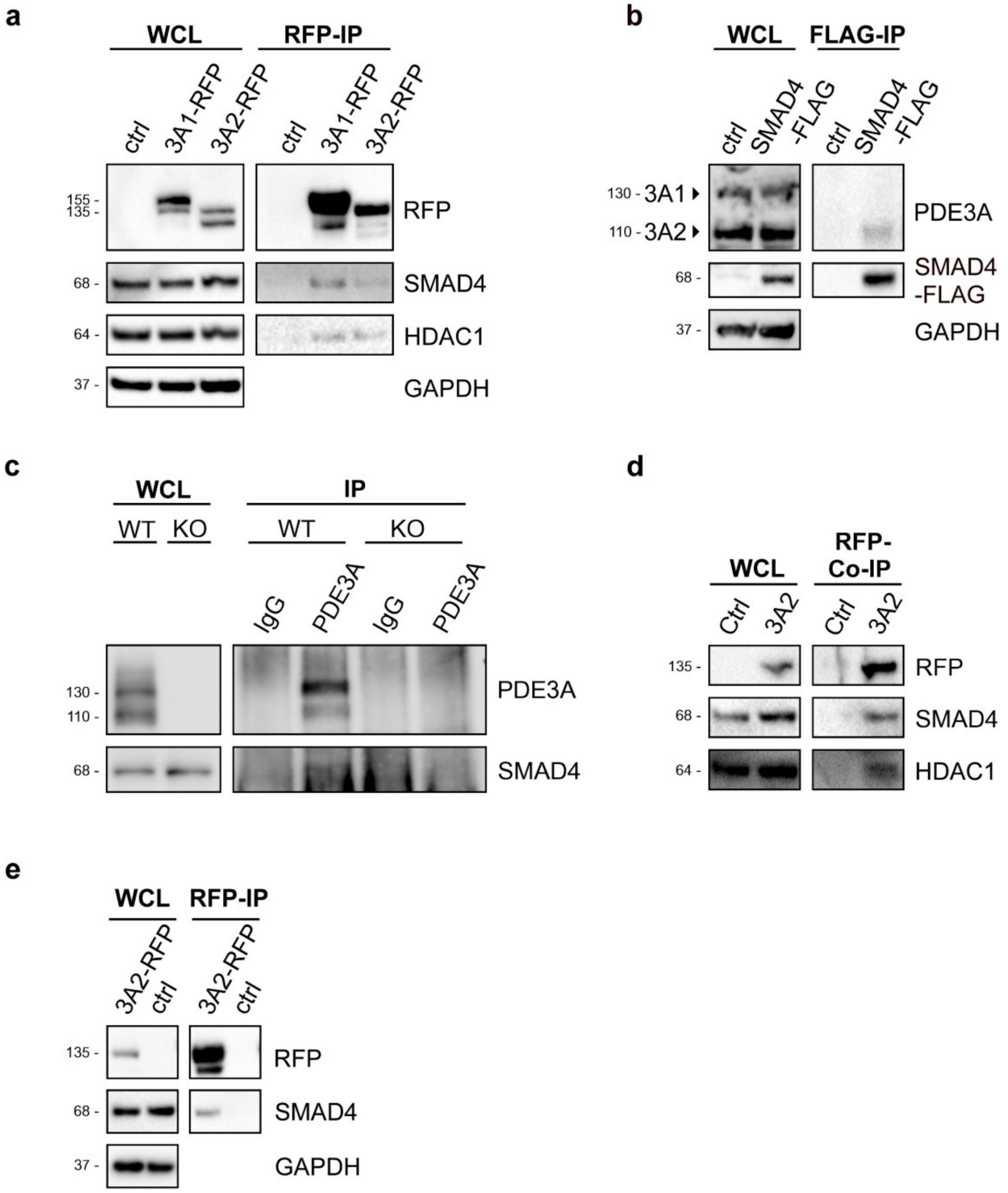

Supplementary Figure 8 | (a) Western blot analysis of RFP pull down obtained from NRVM lysates of cells expressing PDE3A1-RFP or PDE3A2-RFP shows SMAD4 and HDAC-1 in the immunoprecipitate. WCL indicates whole cell lysate. Representative of n=3 independent cultures. **(b)** Detection of endogenous PDE3A after immunoprecipitation from lysates of NRVM expressing Flag-SMAD4-flag. A band corresponding to the size of endogenous PDE3A2 but not PDE3A1 is observed. n=3 **(c)** Representative western blot showing co-immunoprecipitation of endogenous SMAD4 in pull-downs of endogenous PDE3A from tissue lysates obtained from wild type (WT) and PDE3A knockout (KO) rat heart tissues. The rat KO model carries a homozygous frame-shift mutation (Δ20 bp) in the PDE3A gene that results in a functional deletion phenotype[10] . IgG pulldown was used as negative control. n=2. **(d)** Representative western blot showing that SMAD4 and HDAC-1 are detected in the RFP immunoprecipitate (RFP-Co-IP) obtained from ARVM overexpressing PDE3A2-RFP. Representative of three independent experiments **(e)** Representative western blot of RFP immunoprecipitates (RFP-IP) obtained from lysates generated from human iPSC derived cardiomyocytes (hiPSC-CM) overexpressing PDE3A2-RFP and probed with RFP and SMAD4 specific antibodies. Representative of n = 2 independent differentiations.

Supplementary Figure 9

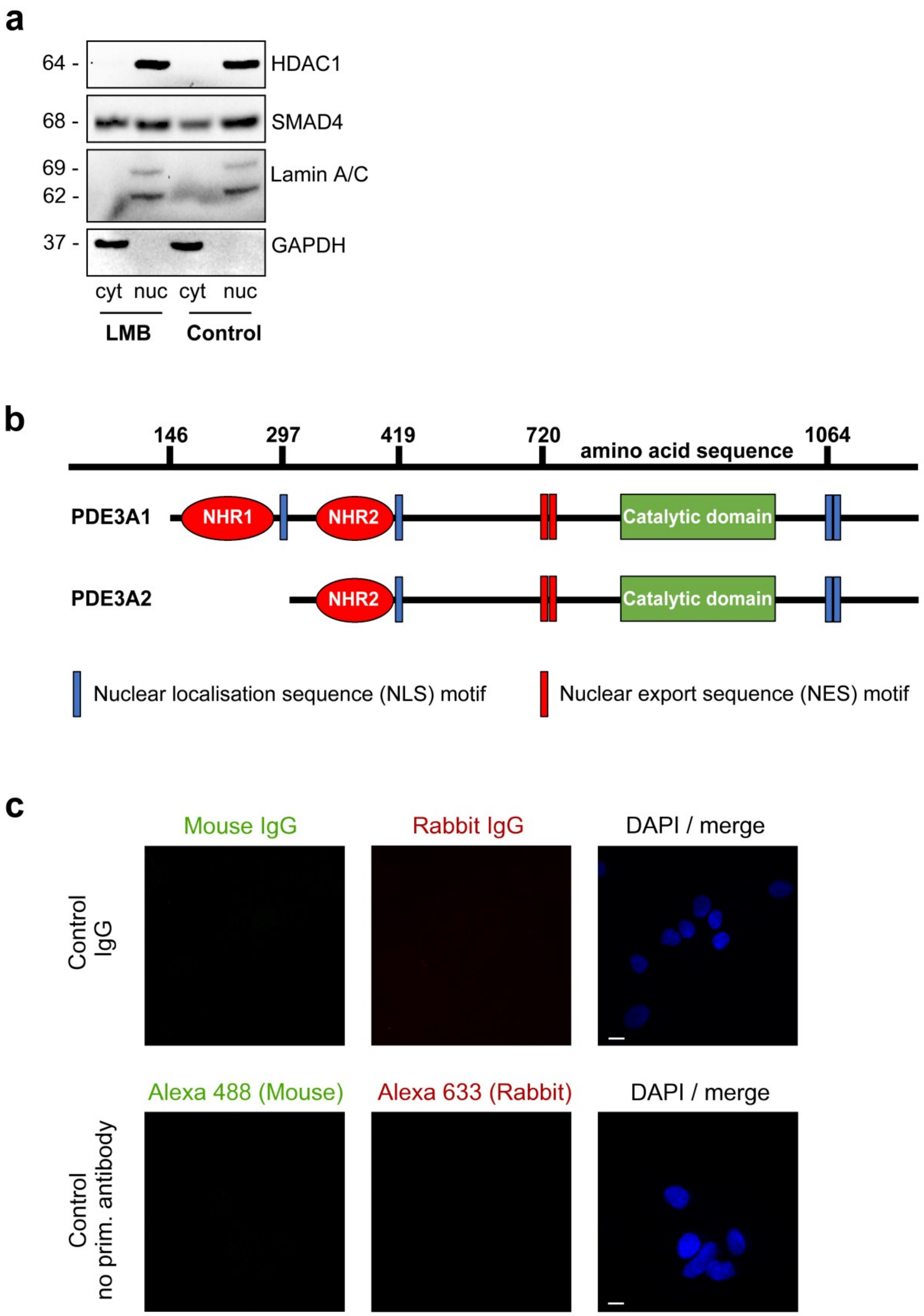

Supplementary Figure 9 | (a) Western blot analysis of cytoplasmic (cyt) and nuclear (nuc) fractions from NRVM cells treated with 100nM leptomycin B (LMB) or DMSO (control) for 3hrs and probed with antibodies against HDAC1 and SMAD4. GAPDH and Lamin A/C proteins were used as markers for cytoplasmic and nuclear content. **(b)** Schematic representation of PDE3A1 and PDE3A2 depicting the nuclear localisation sequence (NLS) and nuclear export sequence (NES) motifs. Amino acids (aa) length is given at the top. Catalytic domain and membrane-associated N-terminal hydrophobic regions 1 and 2 (NHR1 and 2) are indicated. (c) Antibody specificity assessment. Top: Representative images of NRVM treated with mouse IgG and rabbit IgG followed by goat anti-mouse Alexa 488 and goat anti-rabbit Alexa 633 antibody, as indicated. DAPi nuclei staining is also shown. Bottom: immunostaining of NRVM with goat anti-mouse Alexa 488 and goat anti-rabbit Alexa 633 antibody in the absence of primary antibody. Nuclei are stained with DAPI. Bar size = 10 μm

Supplementary Figure 10

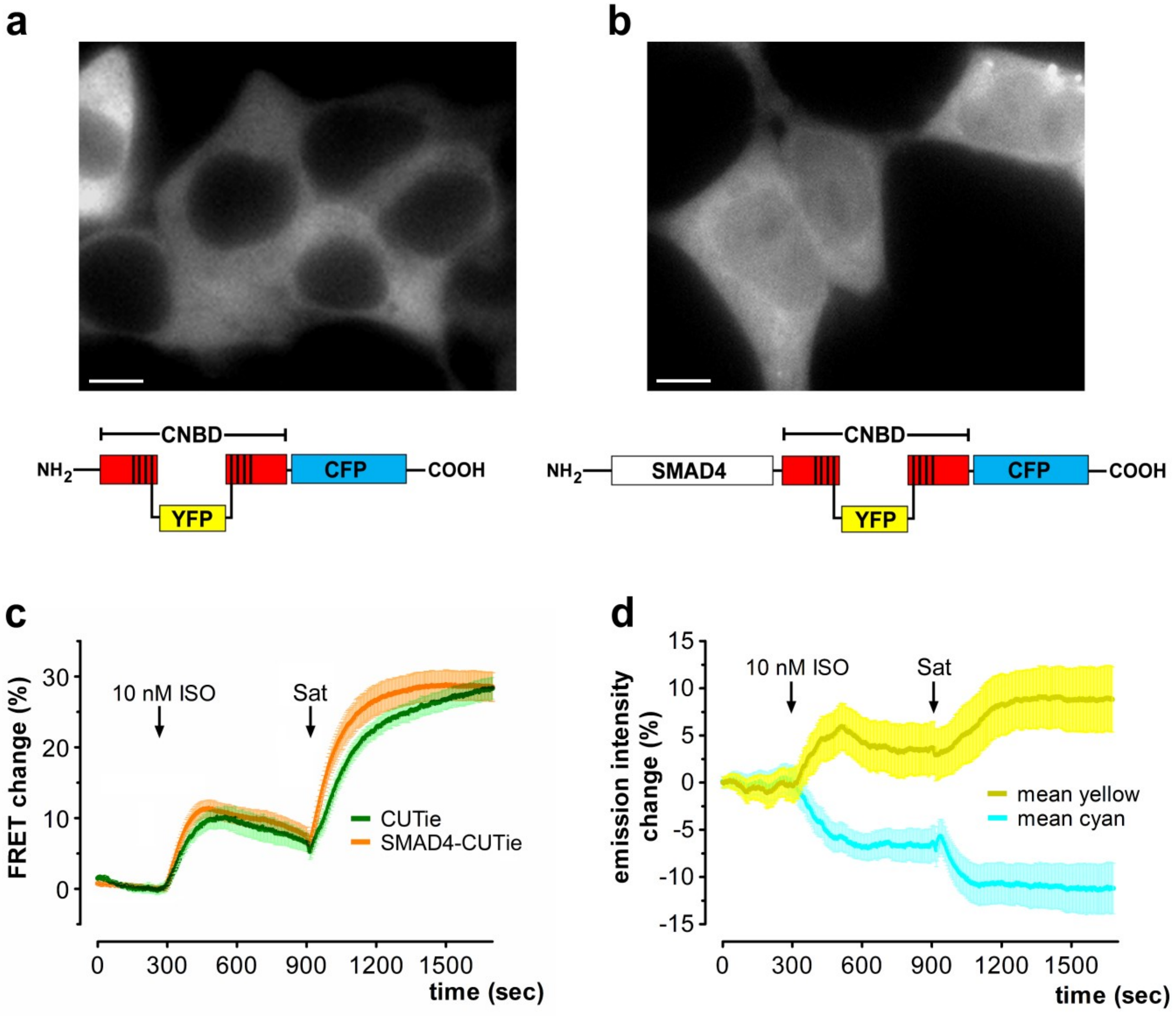

Supplementary Figure 10 | Generation of a FRET reporter for detection of cAMP in proximity of SMAD4. Schematic representation and subcellular distribution in HEK293T cells of the cytosolic cAMP reporter CUTie **(a)** and of the SMAD4-targeted version, SMAD4-CUTie **(b).** CNBD, cyclic nucleotide binding domain. **(c)** Kinetics of FRET change recorded in HEK293T cells expressing CUTie or SMAD4-CUTie on application of ISO or saturating stimulus (SAT, 25μM forskolin + 100μM IBMX). In all experiments cells were pre-treated 100nM leptomycin B (LMB) for 3 hr. Curves are means +/- SEM, with an n of 8 cells (SMAD4-CUTie) and 10 cells (CUTie), measured in at least 4 independent experiments. (**d**) Representative changes of fluorescence intensity generated by the SMAD4-CUTie sensor under the same condition as in (c). Bar size = 5 μm

Supplementary Figure 11

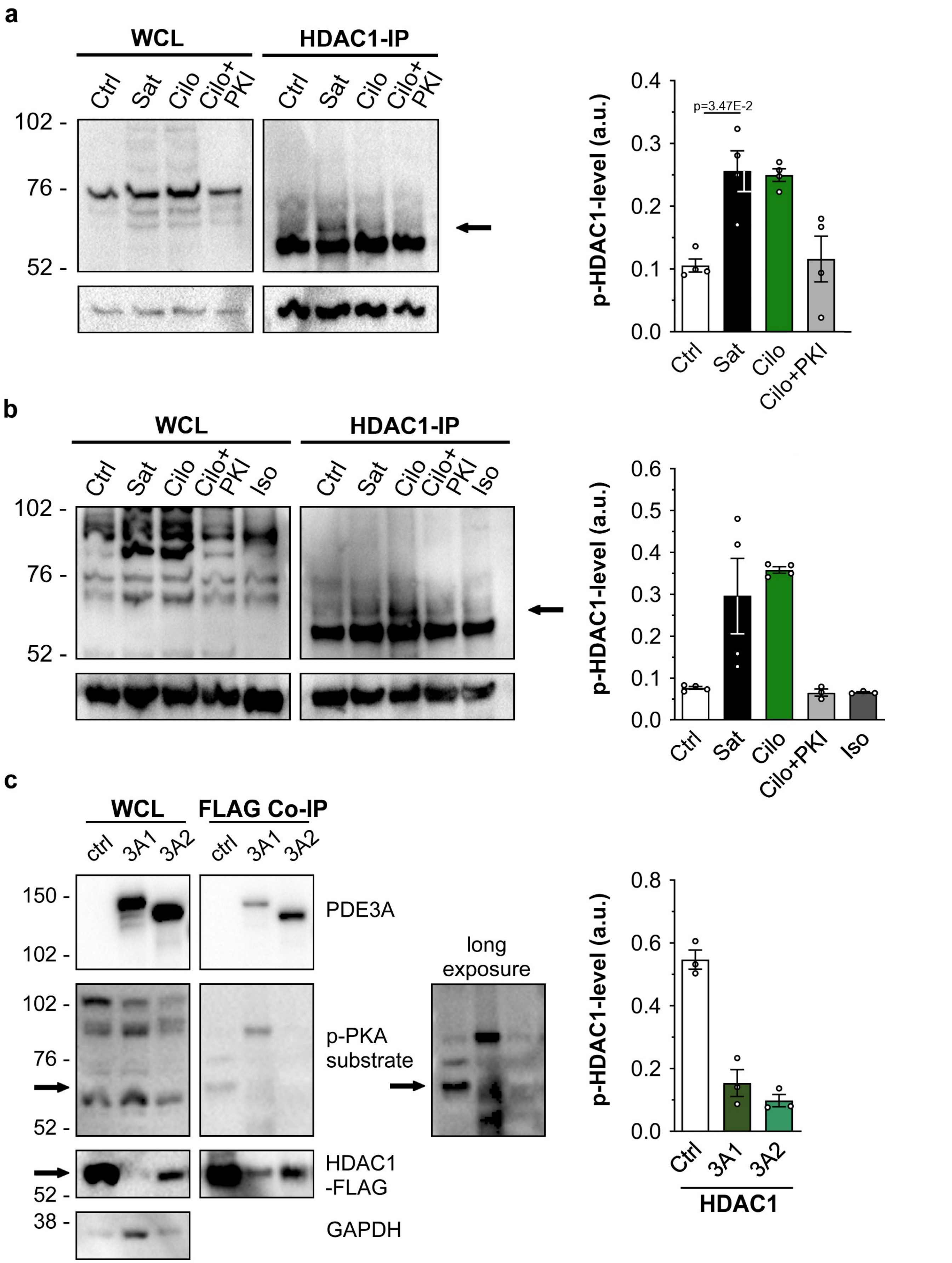

Supplementary Figure 11 | PDE3A controls PKA-dependent HDAC-1 phosphorylation. Representative **w**estern blot analysis and quantification of HDAC-Flag pulldown obtained from ARVM **(a**) and hiPSC-CM **(b)** treated as indicated and probed with PKA substrate antibody. **(c)** Representative image and (**b**) densitometric quantification showing western blot analysis of whole cell lysates (WCL) and HDAC1-Flag immunoprecipitates (Flag-CoIP) obtained from HEK293 cells overexpressing HDAC-Flag alone or in combination with either PDE3A1 or PDE3A2. PKA-specific phosphorylation was detected using a PKA-substrate specific antibody. Data are normalised to the amount of HDAC1-Flag in the immunoprecipitate and are presented as relative to untransfected control cells (Ctrl) that do not overexpress PDE3A isoforms. Values are means ± s.e.m, n=3 independent experiments. For all datasets, one-way ANOVA and Bonferroni's multiple comparison tests were used. ***p<0.001.

Supplementary Figure 12

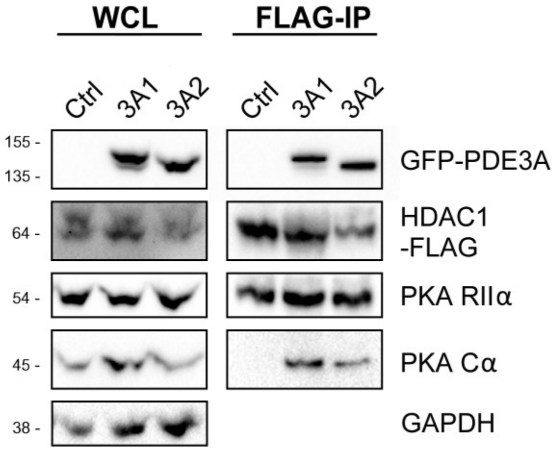

Supplementary Figure 12 | Interaction of PKA holoenzyme with the SMAD4/HDAC-1 complex requires PDE3A. Western blot analysis showing that PKA regulatory (RIIα) and catalytic (Cα) subunits are both present in the FLAG pulldown obtained from lysates of HEK 293 cells expressing HDAC-1-Flag only when either PDE3A1 or PDE3A2 are co-expressed. In the absence of PDE3A isoforms only PKA RIIα subunit can be detected. Representative of 3 independent experiments

Supplementary Figure 13

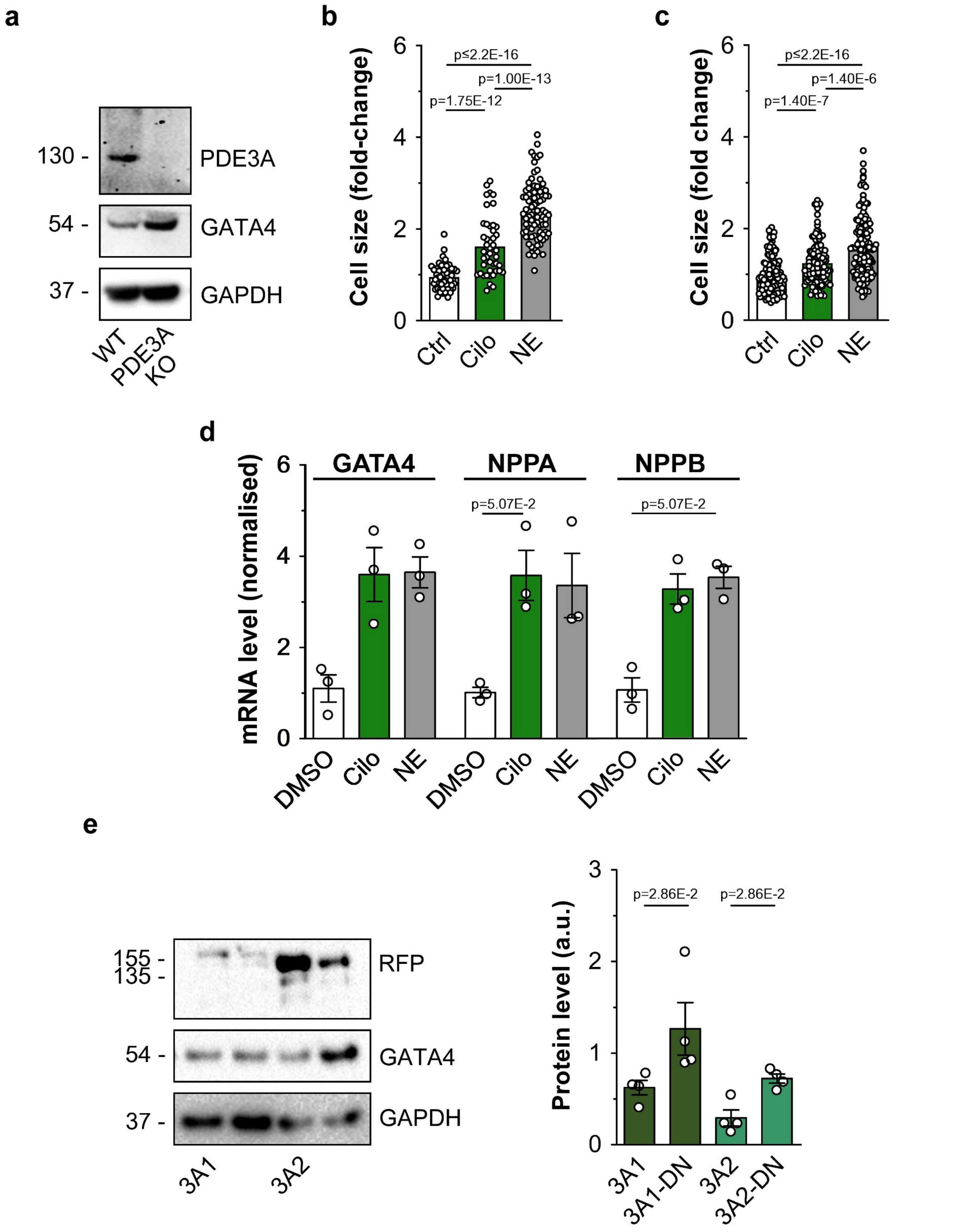

Supplementary Figure 13 | Displacement of PDE3A isoforms enhances GATA4 expression and affects cardiac myocyte hypertrophic growth. **(a)** Western blot analysis of GATA4 expression in cardiac tissue lysates obtained from wild type controls (WT) or PDE3A KO mice. Representative of 2 independent experiments. **(b)** Cell size measured in NRVM either untreated or treated with CILO (10μM) or NE (10μM) for 48 hrs. Values are normalised to DMSO control and expressed as means ± s.e.m. n = 2 independent experiments (at least 44 cells per condition). Hierarchical analysis with final Bonferroni correction of log-transformed data. **(c)** Cell size measured in hiPSC-CM either untreated or treated with CILO (10μM) or NE (10μM) for 7 days. Values are normalised to control and expressed as means ± s.e.m. n = 3 independent experiments (at least 47 cells per condition) Hierarchical analysis with final Bonferroni correction of log-transformed data. **(d)** mRNA level of hypertrophy markers determined by qPCR analysis of hiPSC-CM treated as in (c). Kruskal-Wallis test **(e)** Western blot analysis of GATA4 protein expression in NRVM cells overexpressing wild type (WT) PDE3A1-RFP or PDE3A2-RFP, or the respective dominant negative (DN) catalytically inactive mutants. GAPDH served as a loading control. Bar diagram represents densitometric quantification of GATA4 expression normalised to GAPDH and RFP. Values are presented as means ± s.e.m. n = 4 independent experiments. Mann-Whiney test.

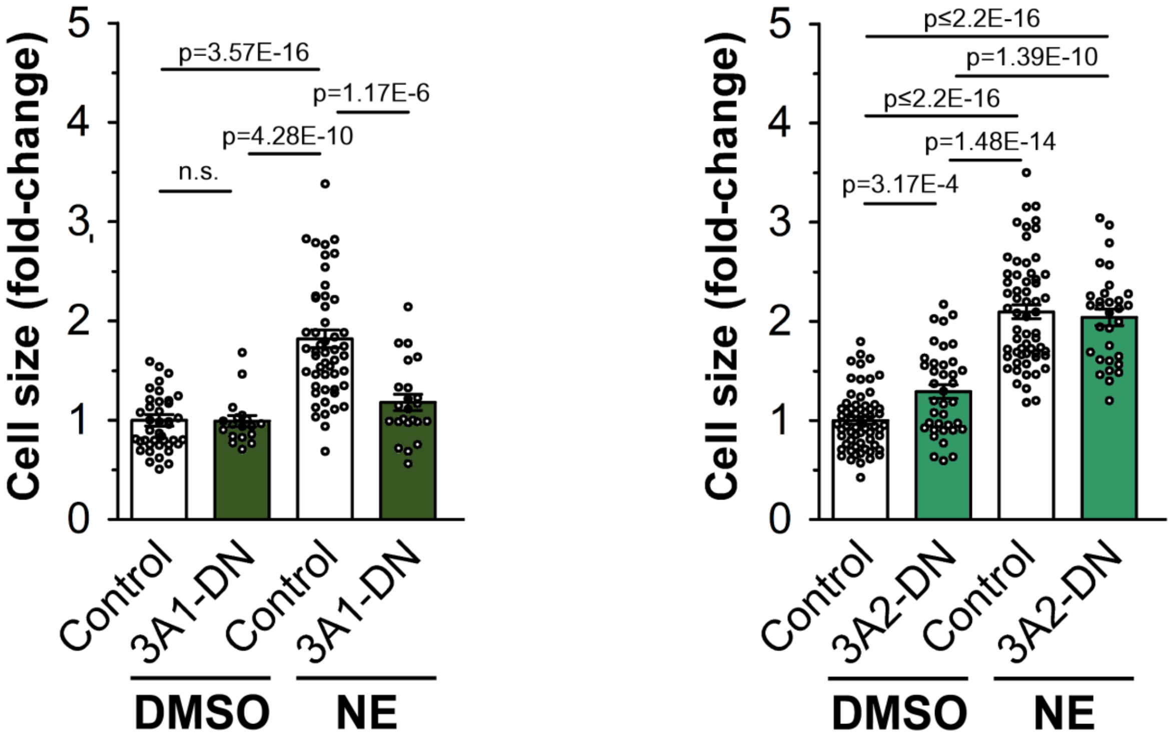

Supplementary Figure 14| Effect of overexpression of catalytically inactive PDE3A1 and PDE3A2 on cardiac myocyte hypertrophic growth. NRVM cell size measured in cardiac myocytes either untransfected or transfected with the catalytically inactive mutant PDE3A1-DN-RFP (left panel) or PDE3A2-DN-RFP (right panel) and treated as indicated for 48 hrs. n = 2 independent experiments. Significances determined using a hierarchical followed by Bonferroni's correction of log-normal data.

Supplementary Figure 15

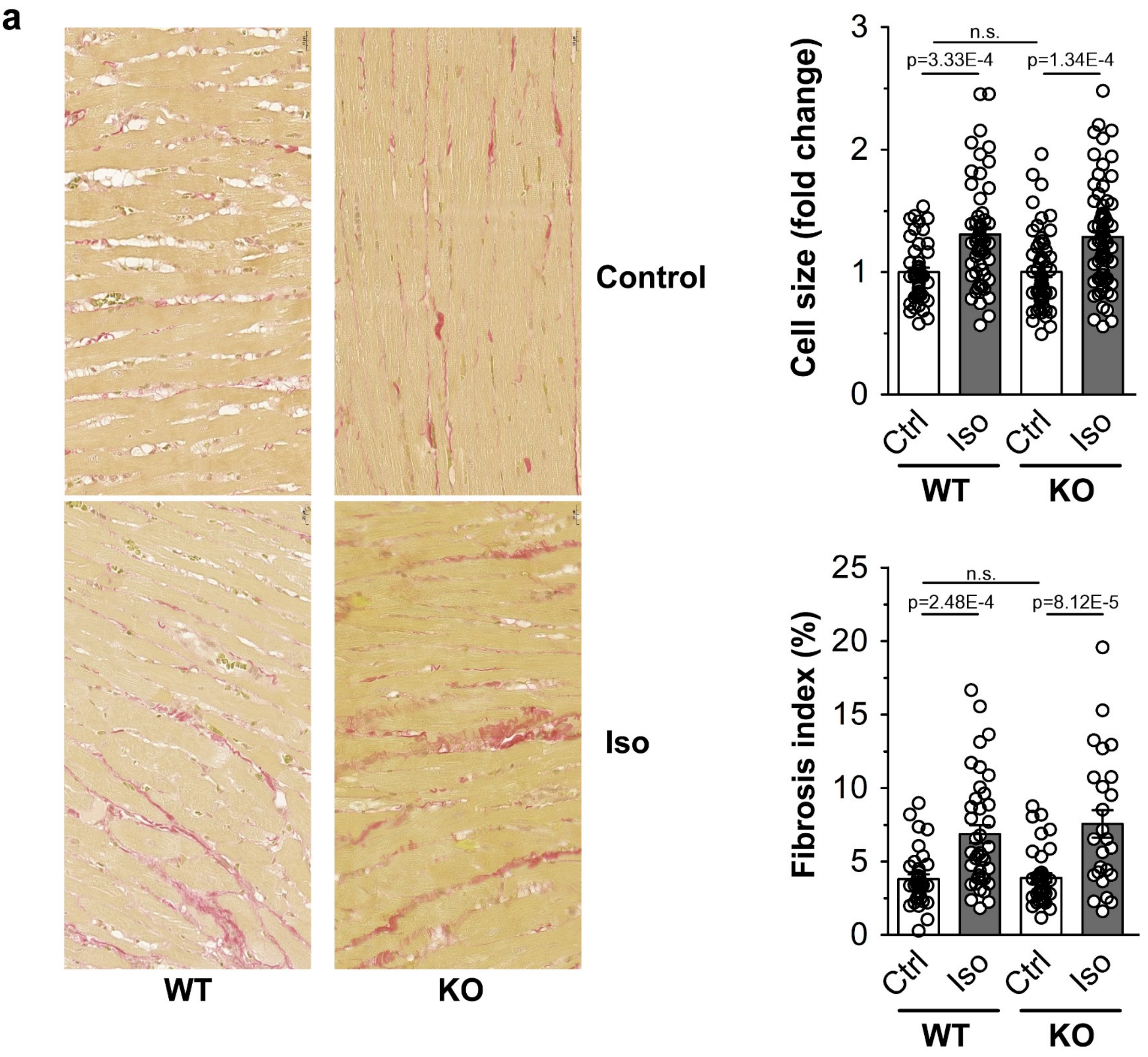

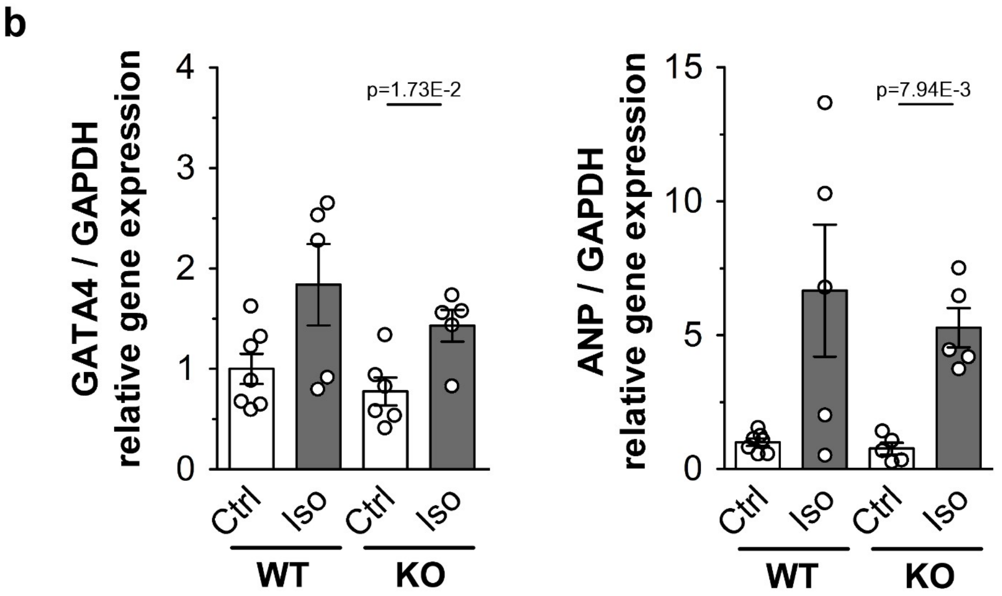

Supplementary Figure 15 | Effects of chronic β-adrenergic challenge on PDE3 KO hearts. Hearts from rats carrying a PDE3A genetic functional deletion mutation or wild-type (WT) rats were implanted with osmotic minipumps for administration of isoproterenol (Iso) or physiological saline (NaCl) for 2 weeks. **(a)** Cardiac sections were stained with Picro Sirius Red. At least 25 non-overlapping image fields from 5 different samples in each experimental group were analysed using ImageJ. Cardiac hypertrophy was evaluated by measuring the cross-sectional area of cardiomyocytes. The fibrosis index (%) was calculated as a percentage of collagen-positive areas to the total area of the image. Statistical analysis was carried out using a Kruskal-Wallis and Dunn's multiple-comparison test. **(b)**. Quantification of hypertrophy markers by qPCR analysis of samples as described in (a). n = at least 4 independent samples. Kruskal-Wallis and Dunn's multiple-comparison test.

Supplementary Table 1

Site	Gene	Protein
195	Abcf1	ATP-binding cassette sub-family F member 1
230	Acsl1	Long-chain-fatty-acid--CoA ligase 1
553	Canx	Calnexin
287	Ctnna3	Catenin alpha 3
162	Eef1d	Elongation factor 1-delta
564	Hrc	Histidine rich calcium binding protein, isoform CRA_b
263	Hsp90aa1	Heat shock protein HSP 90-alpha
60	Kpna3	Importin subunit alpha
288	Mybpc3	Myosin-binding protein C, cardiac-type
1094	Myh6	Myosin-6
1960	Myo18a	Myosin XVIIIa
226	Osbp	Oxysterol-binding protein
17	Plin5	Perilipin-5
789	Rbm20	RNA-binding protein 20
304	Rplp0	60S acidic ribosomal protein P0
102	Rplp2	60S acidic ribosomal protein P2
22	Slc25a4	ADP/ATP translocase 1
47	Smpx	Small muscular protein
141	Synpo2l	Synaptopodin 2-like
107	Szrd1	SUZ domain-containing protein 1
315	Thrap3	Thyroid hormone receptor-associated protein 3
174	Ybx1	Nuclease-sensitive element-binding protein 1

Table S1 | *Phosphorylation sites upregulated on inhibition of both PDE3 and PDE2A.*
