# Supplementary material for: Integrated Proteomics Unveils Nuclear PDE3A2 as a Regulator of Cardiac Myocyte Hypertrophy
Source: Circ Res. 2023 Mar 8;132(7):828–48. doi: 10.1161/CIRCRESAHA.122.321448 (PMC10045983; doi:10.1161/CIRCRESAHA.122.321448)
Supplement: Supplementary file 6 [file res-132-828-s006.pdf]

Major Resources Table

In order to allow validation and replication of experiments, all essential research materials listed in the Methods should be included in the Major Resources Table below. Authors are encouraged to use public repositories for protocols, data, code, and other materials and provide persistent identifiers and/or links to repositories when available. Authors may add or delete rows as needed.

Animals (in vivo studies)

Species	Vendor or Source	Background Strain	Sex	Persistent ID / URL
**Species**	**Vendor or Source**	**Background Strain**	**Sex**	**Persistent ID / URL**
Rattus norvegicus	In house breeding	WT	Male	13452
Rattus norvegicus	In house breeding	WT	Male	13453
Rattus norvegicus	In house breeding	WT	Male	13844
Rattus norvegicus	In house breeding	WT	Male	13845
Rattus norvegicus	In house breeding	WT	Male	13846
Rattus norvegicus	In house breeding	WT	Male	18246
Rattus norvegicus	In house breeding	WT	Male	18249
Rattus norvegicus	In house breeding	WT	Male	13454
Rattus norvegicus	In house breeding	WT	Male	13774
Rattus norvegicus	In house breeding	WT	Male	18240
Rattus norvegicus	In house breeding	WT	Male	18244
Rattus norvegicus	In house breeding	WT	Male	14315
Rattus norvegicus	In house breeding	KO	Male	14024
Rattus norvegicus	In house breeding	KO	Male	14242
Rattus norvegicus	In house breeding	KO	Male	18212
Rattus norvegicus	In house breeding	KO	Male	18238
Rattus norvegicus	In house breeding	KO	Male	16991
Rattus norvegicus	In house breeding	KO	Male	16999
Rattus norvegicus	In house breeding	KO	Male	14245
Rattus norvegicus	In house breeding	KO	Male	18204
Rattus norvegicus	In house breeding	KO	Male	18233
Rattus norvegicus	In house breeding	KO	Male	14244
Rattus norvegicus	In house breeding	KO	Male	14245
Rattus norvegicus	In house breeding	WT	Male	15392
Rattus norvegicus	In house breeding	WT	Male	18274
Rattus norvegicus	In house breeding	WT	Male	15403
Rattus norvegicus	In house breeding	WT	Male	22958
Rattus norvegicus	In house breeding	WT	Male	22654
Rattus norvegicus	In house breeding	WT	Male	22962
Rattus norvegicus	In house breeding	WT	Male	23017
Rattus norvegicus	In house breeding	KO	Male	19677
Rattus norvegicus	In house breeding	KO	Male	19672
Rattus norvegicus	In house breeding	KO	Male	19916
Rattus norvegicus	In house breeding	KO	Male	00350
Rattus norvegicus	In house breeding	KO	Male	00351
Rattus norvegicus	In house breeding	KO	Male	00352
Rattus norvegicus	In house breeding	KO	Male	00353

We are aware that physiological and pathophysiological processes in the bodies of humans and animals may be sex-dependent. Such sex-dependent differences in severity and symptomatology have also been observed in cardiovascular patients and in animal models. However, for this experiment we have chosen to initially select only male animals because

DOI [to be added]

male animals usually react more sensitively with blood pressure increases than female animals, which can be protected from blood pressure increases by their sex hormones.

In future experiments, we will also use female animals to confirm that our findings are sex-independent.

Another reason for limiting the experiments to the use of males was German animal legislation, which is particularly restrictive in Berlin, and authorities enforce that scientists carry out animal experiments with as few animals as possible.

Antibodies

Target antigen	Vendor or Source	Catalog #	Working concentration	Persistent ID / URL
PDE3A	Bethyl lab	A302-740A	1:1000	https://www.fortislife.com/search?query=pde3a&pageSize=15
PDE3A	Santacruz	SC 293446	1:20	https://www.scbt.com/p/pde3a-antibody-2d7
PDE3A	Novusbio	H00005139-M03	1:50	https://www.novusbio.com/products/pde3a-antibody-2d7_h00005139-m03
Flag	Sigma	F7425	1:2000	https://www.sigmaaldrich.com/GB/en/product/sigma/f7425
GAPDH	ProteinTech	60004-1-1g	1:5000	https://www.ptglab.com/products/GAPDH-Antibody-60004-1-Ig.htm
phosphoPKA substrate antibody	Cell signaling	9621S	1:1000	https://www.cellsignal.com/products/primary-antibodies/phospho-ser-thr-pka-substrate-antibody/9621
phosphoPKA substrate antibody	Cell Signalling	9624	1:1000	https://www.cellsignal.com/products/primary-antibodies/phospho-pka-substrate-rrxs-t-100g7e-rabbit-mab/9624
SMAD4	Cell Signaling	46535	1:1000	https://www.cellsignal.com/products/primary-antibodies/smad4-d3r4n-xp-rabbit-mab/46535
SMAD4	Genetex	GTX01674	1:100	https://www.genetex.com/Product/Detail/SMAD4-antibody-B-8/GTX01674
GATA4	Cell Signaling	36966	1:1000	https://www.cellsignal.com/products/primary-antibodies/gata-4-d3a3m-rabbit-mab/36966
HDAC1	Cell Signaling	34589	1:1000	https://www.cellsignal.com/products/primary-antibodies/hdac1-d5c6u-xp-rabbit-mab/34589
Lamin B	Cell Signaling	12586	1:1000	https://www.cellsignal.com/products/primary-antibodies/lamin-b1-d4q4z-rabbit-mab/12586
Lamin A/C	Cell Signaling	4777s	1:1000	https://www.cellsignal.com/products/primary-antibodies/lamin-a-c-4c11-mouse-mab/4777?site-search-type=Products&N=4294956287&Ntt=4777s&fromPage=plp&_requestid=599758
Actin1 alpha	Proteintech	17521-1-AP	1:1000	https://www.ptglab.com/products/ACTA1-skeletal-Muscle-Actin-Specific-Antibody-17521-1-AP.htm
RFP	Abcam	Ab62341	1:2000	https://www.abcam.com/rfp-antibody-ab62341.html
PKAα cat	Santacruz	Sc-28315	1:1000	https://www.scbt.com/p/pkaalpha-cat-antibody-a-2

DOI [to be added]

PKARIIα	Santacruz	Sc-908	1:1000	https://www.scbt.com/p/pka-iialpha-reg-antibody-c-20
GFP	abcam	Ab6556	1:1000	https://www.abcam.com/gfp-antibody-ab6556.html
Anti mouse HRP	SCBT	Sc2005	1:5000	https://www.scbt.com/p/goat-anti-mouse-igg-hrp
Anti mouse HRP	Jackson immuno research lab	115-035-003	1:5000	https://www.jacksonimmuno.com/catalog/products/115-035-003
Anti Rabbit HRP	SCBT	Sc2030	1:5000	https://www.scbt.com/p/goat-anti-rabbit-igg-hrp-cruz-marker-compatible
Anti rabbit HRP	Jackson immuno research lab	11-035-003	1:5000	https://www.google.com/search?q=11-035-003&rlz=1C1CHBF_enGB833GB833&oq=11-035-003&aqs=chrome..69i57.678j0j4&sourceid=chrome&ie=UTF-8
Alexaflour 647	Thermo Fischer Scientific	A-21235	1:350	https://www.thermofisher.com/antibody/product/Goat-anti-Mouse-IgG-H-L-Cross-Adsorbed-Secondary-Antibody-Polyclonal/A-21235
Alexaflour 488	Thermo Fischer Scientific	A-11008	1:350	https://www.thermofisher.com/antibody/product/Goat-anti-Rabbit-IgG-H-L-Cross-Adsorbed-Secondary-Antibody-Polyclonal/A-11008

DNA/cDNA Clones

Clone Name	Sequence	Source / Repository	Persistent ID / URL
Flag SMAD4	Clone Id Ora42717	Genescript	https://www.genscript.com/gene/rattus-norvegicus/50554/smad4.html
Flag HDAC1	Clone Id Ora13308	Genescript	https://www.genscript.com/gene/rattus-norvegicus/297893/hdac1.html
mCherrry	Cat # 1767	Vector Biolabs	https://www.vectorbiolabs.com/product/1767-mcherry-adenovirus/
Ad-HDAC1 virus	Cat # 1498	Vector Biolabs	https://www.vectorbiolabs.com/product/1498-histone-deacetylase-1-adenovirus/?azsid=5f965e30-8caa-4c14-99f2-30f63f1ddd14&i=1
Ad-RFP PDE3A1		Vector Biolabs	Customised
Ad-RFP PDE3A2		Vector Biolabs	Customised
Ad-RFP DNPDE3A1		Vector Biolabs	Customised
Ad-RFP DNPDE3A2		Vector Biolabs	Customised

Name	Vendor or Source	Sex (F, M, or unknown)	Persistent ID / URL
hiPSC line LUMCi027-A-1	Leiden University Medical Center, LUMC	Female	https://hpscreg.eu/cell-line/LUMCi027-A-1
hiPSC line LUMC0099iCTRL04	Leiden University Medical Center, LUMC	Female	https://hpscreg. eu/cell-line/LUMCi004-A
AX5858	Censo biotechnologies Ltd, Edinburgh, UK	Female	https://axolbio.com/

DOI [to be added]

hiIPSC line M00398C08	A gift from Christian Pinset, Institute for Stem cell Therapy and Exploration of Monogenic diseases (I-Stem, France)	unknown	https://orcid.org/0000-0002-7840-2179
hiIPSC line M00180	A gift from Christian Pinset, Institute for Stem cell Therapy and Exploration of Monogenic diseases (I-Stem, France)	Female	https://orcid.org/0000-0002-7840-2179
HEK 293T "293T (ATCC CRL-3216)"	American Type Culture Collection	female	https://www.atcc.org/products/crl-3216

Data & Code Availability

Description	Source / Repository	Persistent ID / URL
Project files generated from mass spectrometry analysis of PDE interactome and phosphoproteome experiments deposited under the project name “Integrated proteomics unveils regulation of cardiac myocytes hypertrophic growth by a nuclear cAMP nanodomain under the control of PDE3A”	PRIDE (PRoteomics IDEntification Database)	www.ebi.ac.uk/pride<br>Project accession: PXD033773

Other

Description	Source / Repository	Persistent ID / URL
Fluorimetric histone deacetylase kit CS1010	Sigma- Aldrich	https://www.sigmaaldrich.com/
Raeasy kit (Cat no 74104)	Qiagen	http://www.qiagen.com/
QuantiTect® Reverse Transcription Kit (Cat no 205311)	Qiagen	http://www.qiagen.com/
The NE-PER Nuclear and Cytoplasmic Extraction Reagent Kit (Cat No 78333)	Thermo Fischer	https://www.thermofisher.com/uk/en/home.html
iScript™ cDNA Synthesis Kit	Biorad	https://www.bio-rad.com/
Norepinephrine	Sigma- Aldrich	https://www.sigmaaldrich.com/
Cilostamide, isoproterenol	Sigma- Aldrich	https://www.sigmaaldrich.com/
https://www.sigmaaldrich.com/	Sigma- Aldrich	https://www.sigmaaldrich.com/
3-isobutyl-1-methylxanthine (IBMX)	Sigma- Aldrich	https://www.sigmaaldrich.com/
Norepinephrine	Sigma- Aldrich	https://www.sigmaaldrich.com/
BAY 60-7550	Sigma- Aldrich	https://www.sigmaaldrich.com/

DOI [to be added]
