# Supplementary material for: Integrated Proteomics Unveils Nuclear PDE3A2 as a Regulator of Cardiac Myocyte Hypertrophy
Source: Circ Res. 2023 Mar 8;132(7):828–48. doi: 10.1161/CIRCRESAHA.122.321448 (PMC10045983; doi:10.1161/CIRCRESAHA.122.321448)

Full unedited gel for Figure 4a

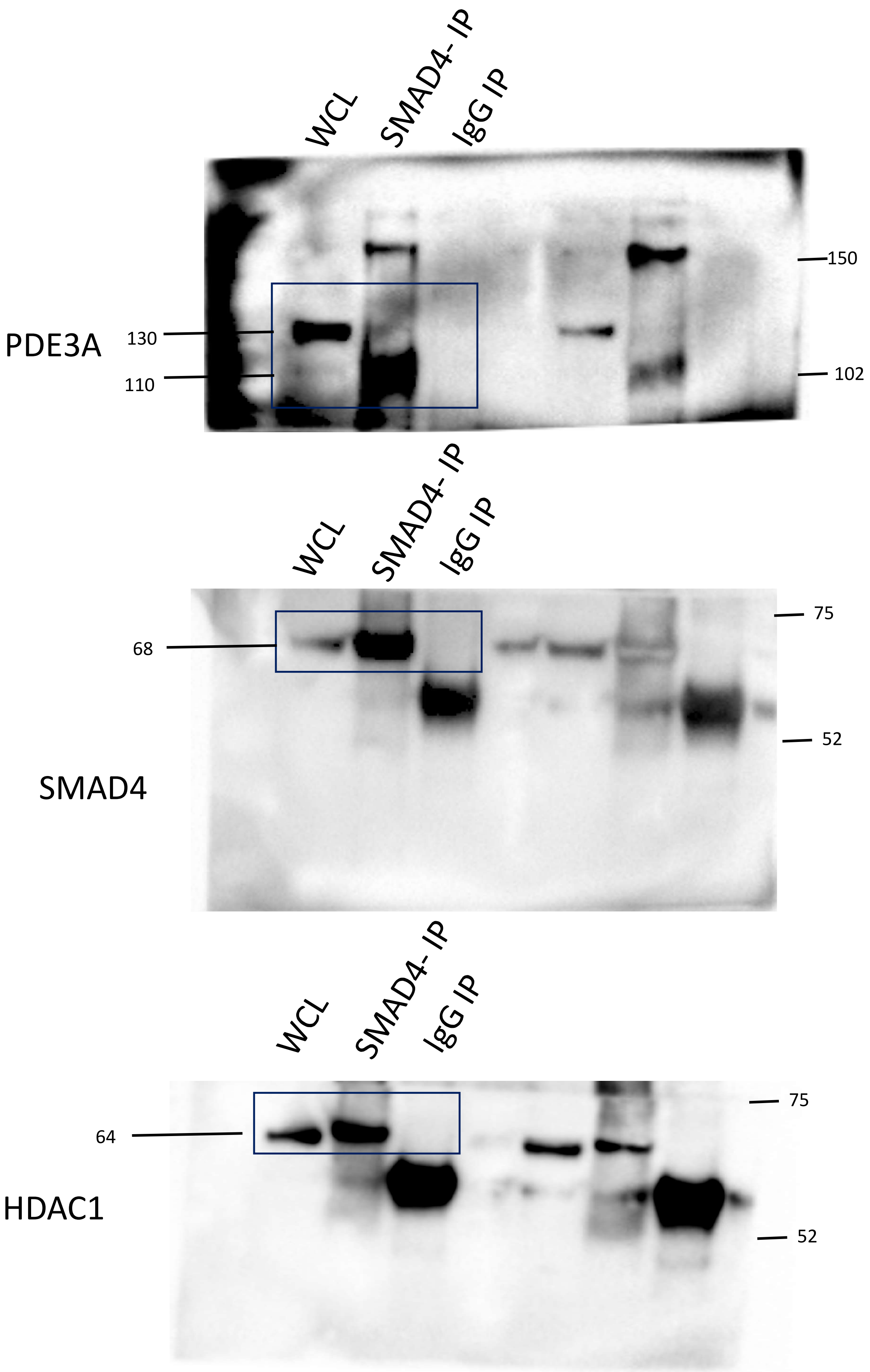

Full unedited gel for Figure 4b

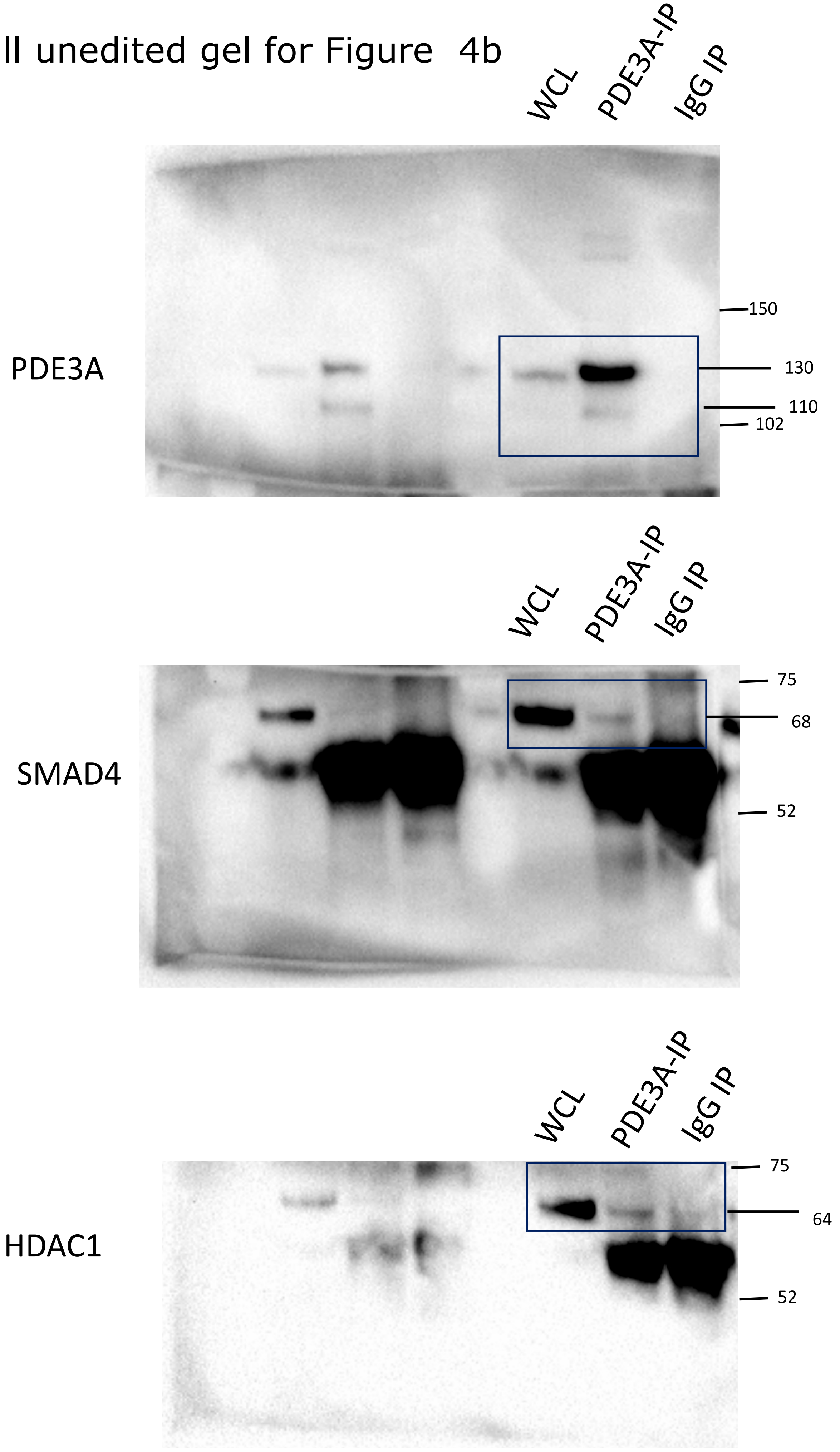

Full unedited gel for Figure 4d

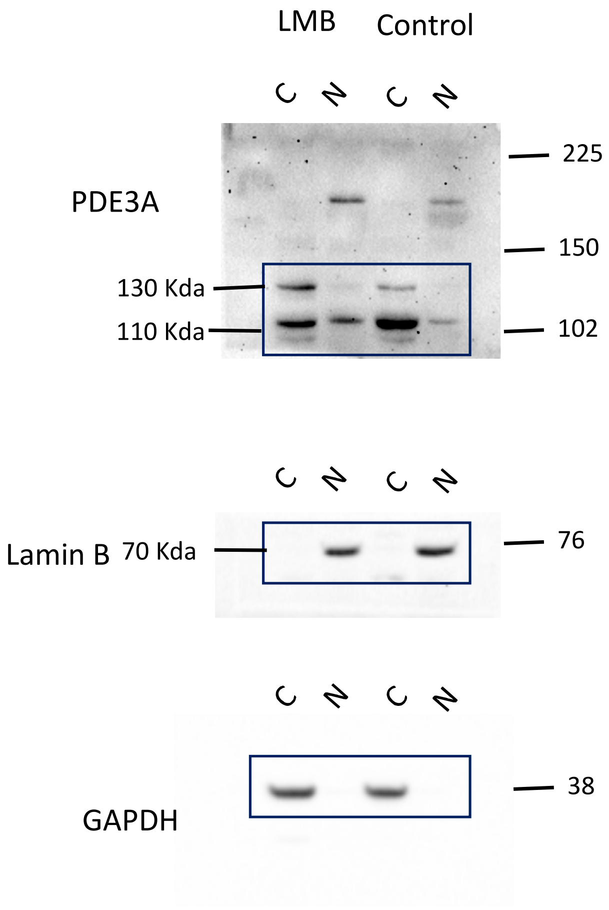

Full unedited gel for Figure 5b

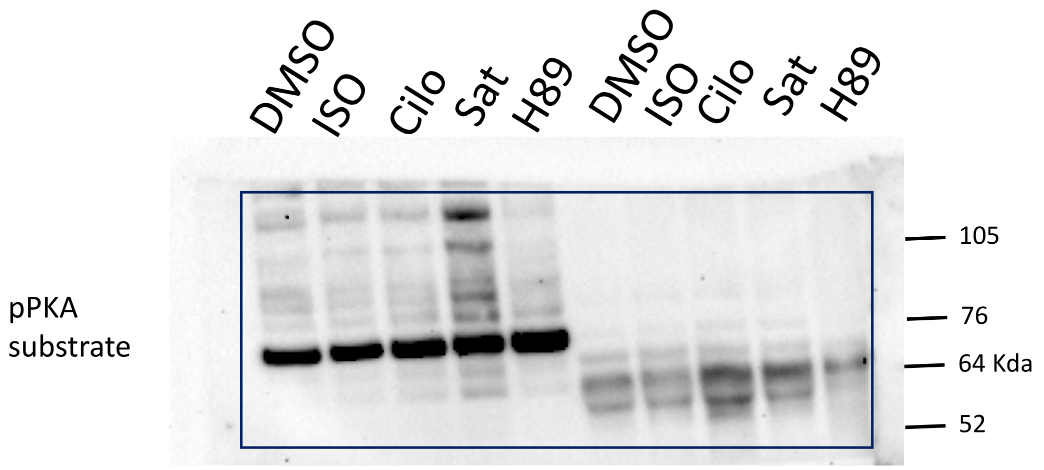

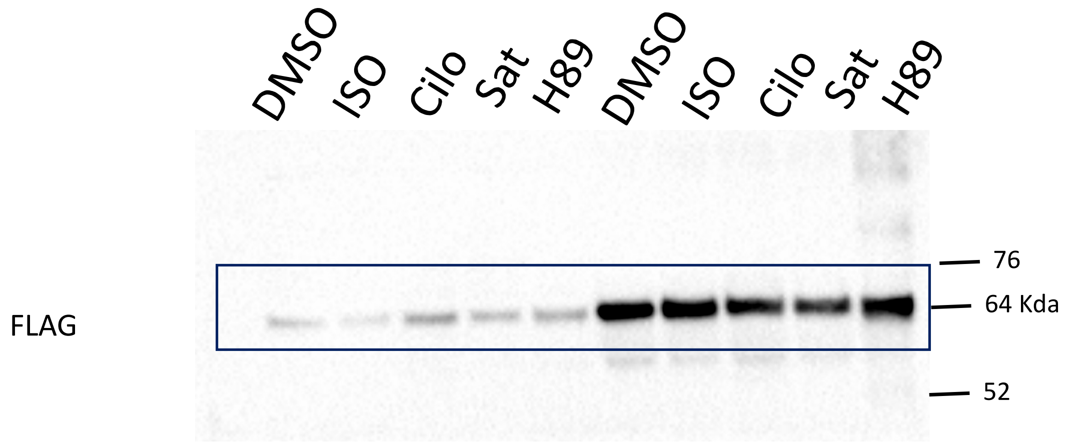

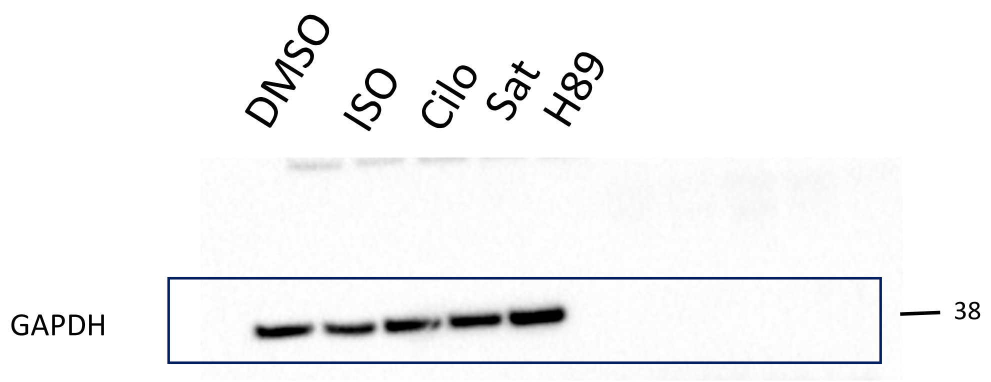

Full unedited gel for Figure 5c

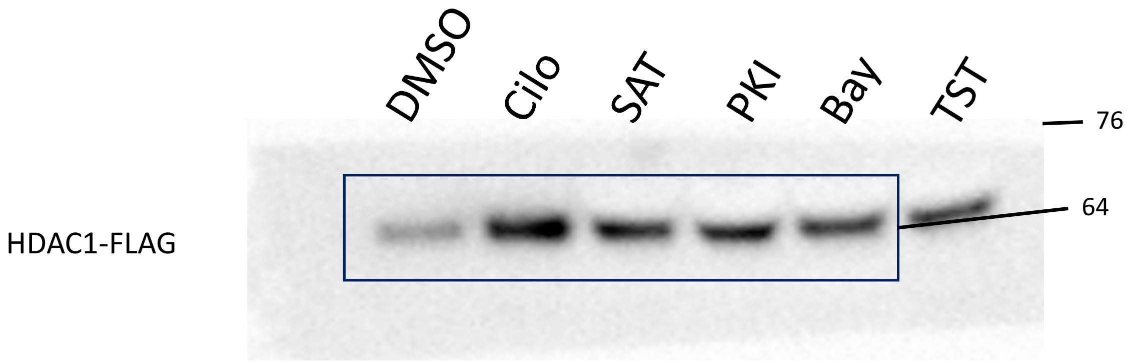

Full unedited gel for Figure 5d

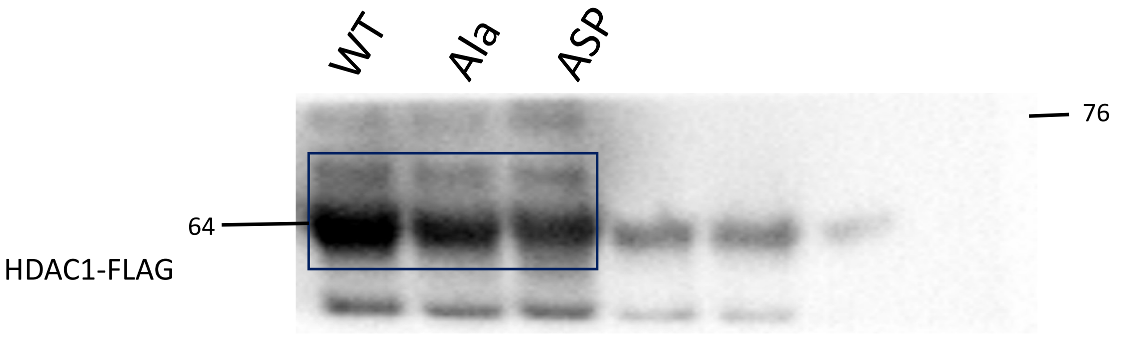

Full unedited gel for Figure 5e

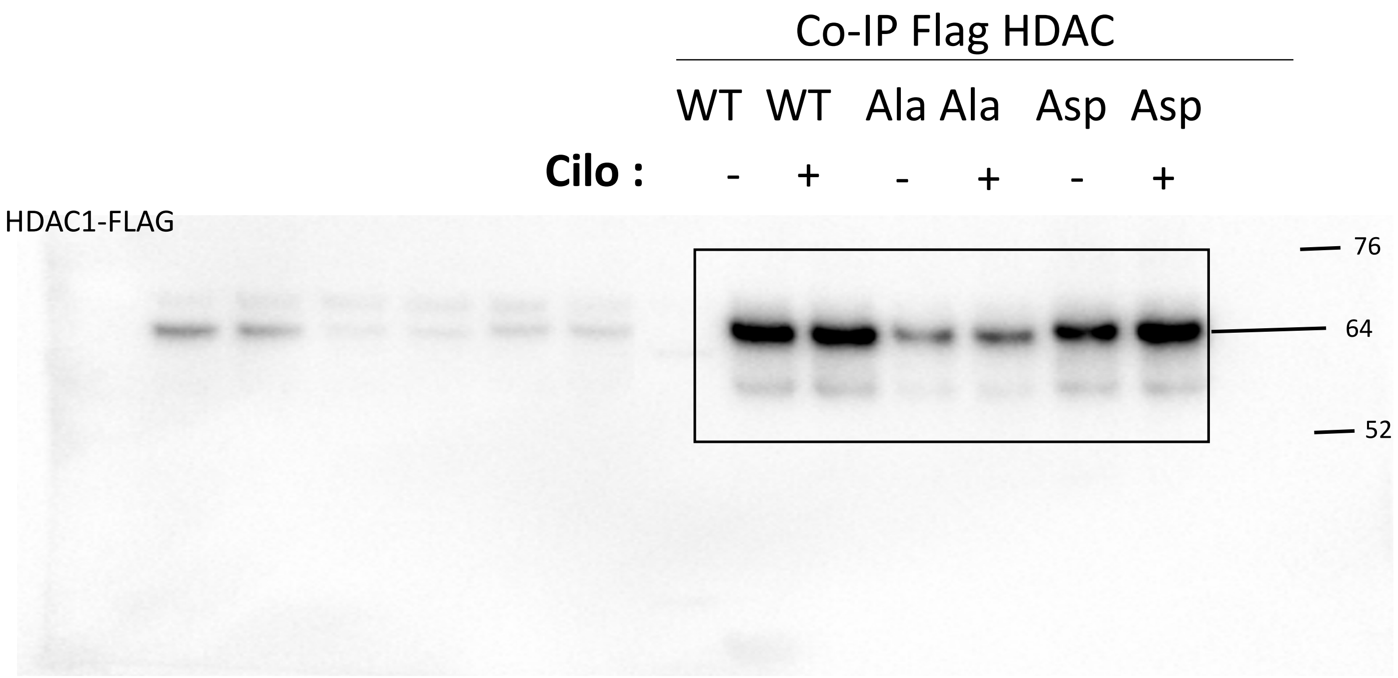

Full unedited gel for Figure 5f

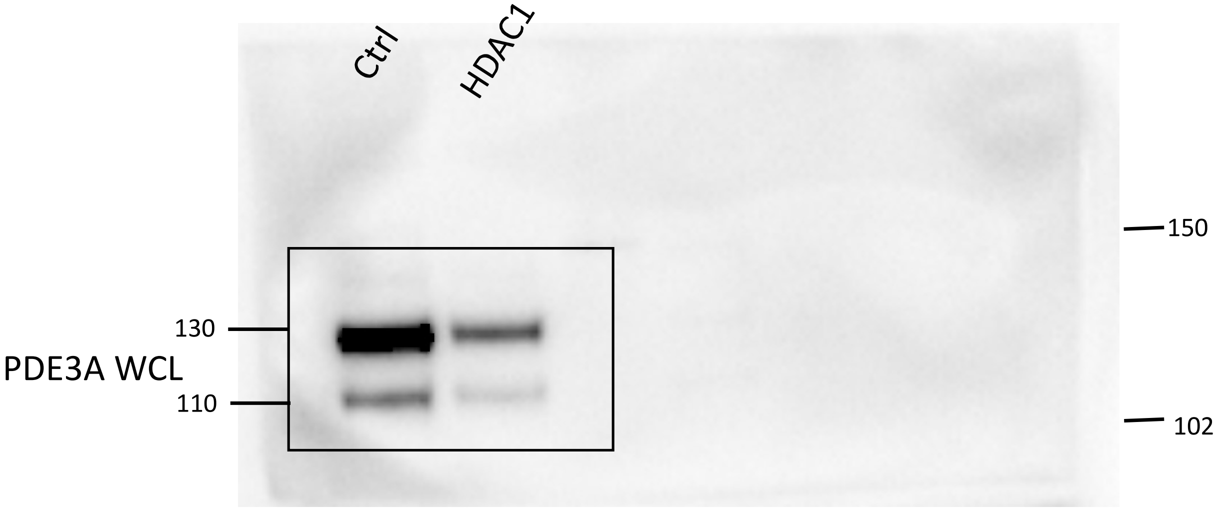

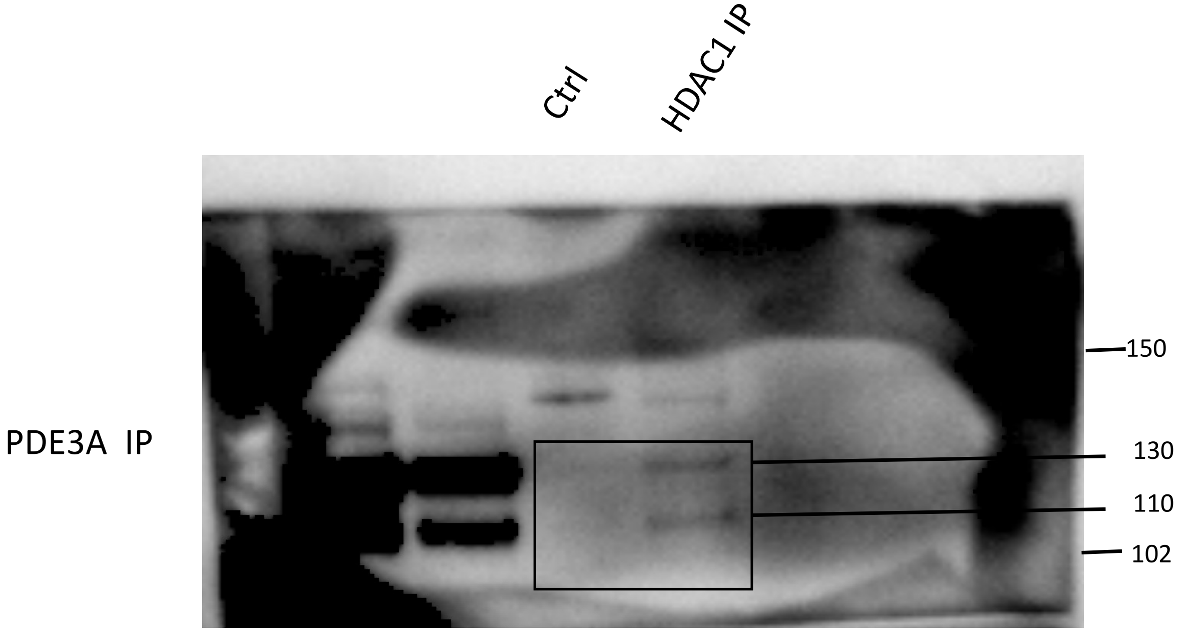

Full unedited gel for Figure 5f

Flag WCL

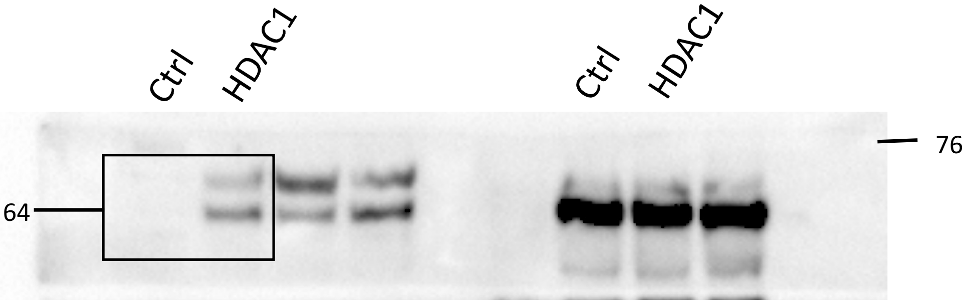

Flag IP

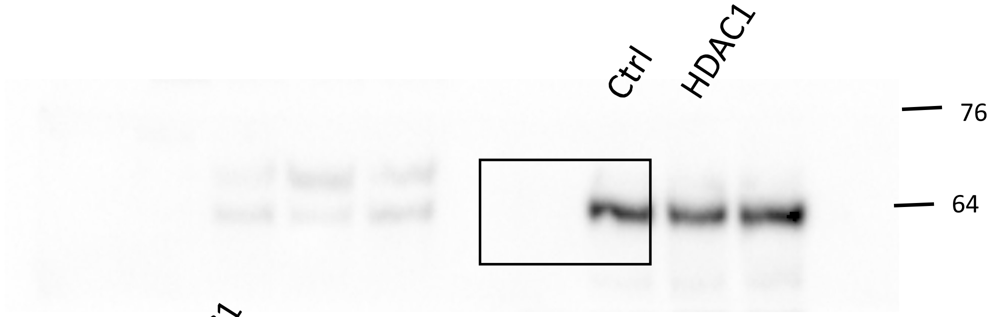

PKA RIIα WCL

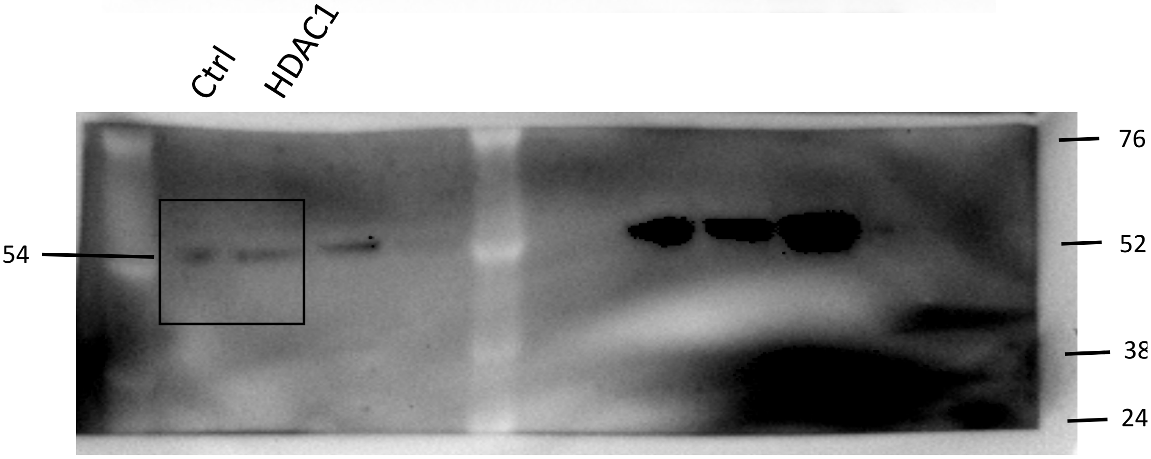

PKA RIIα IP

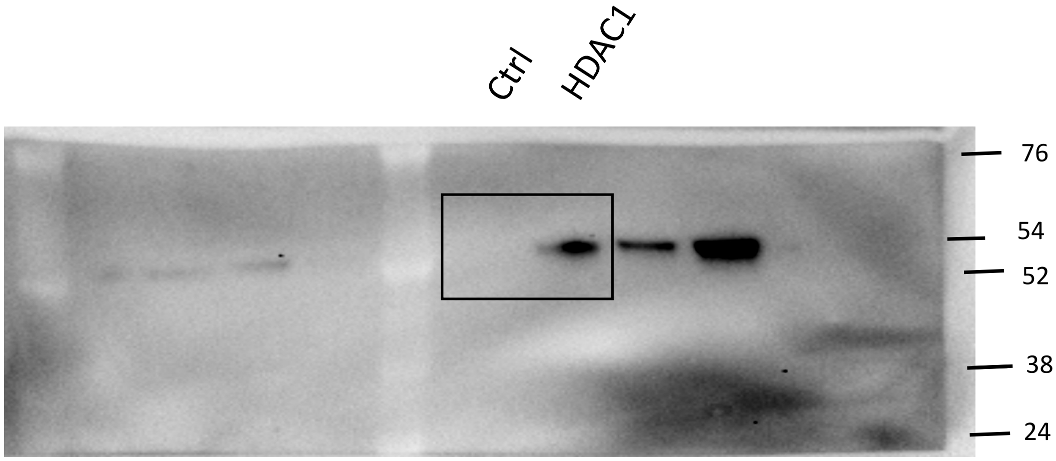

Full unedited gel for Figure 5f

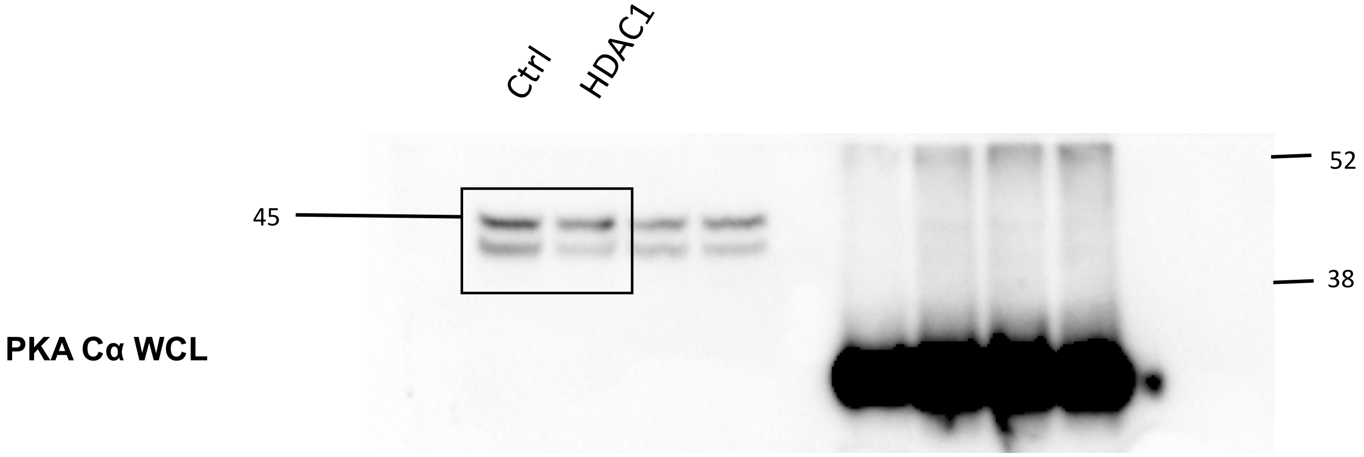

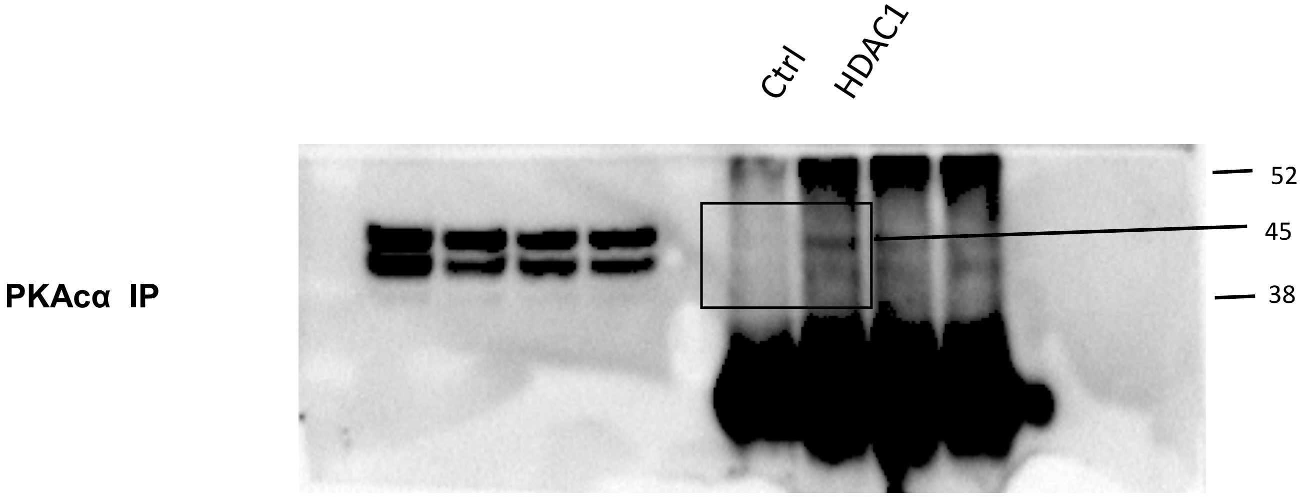

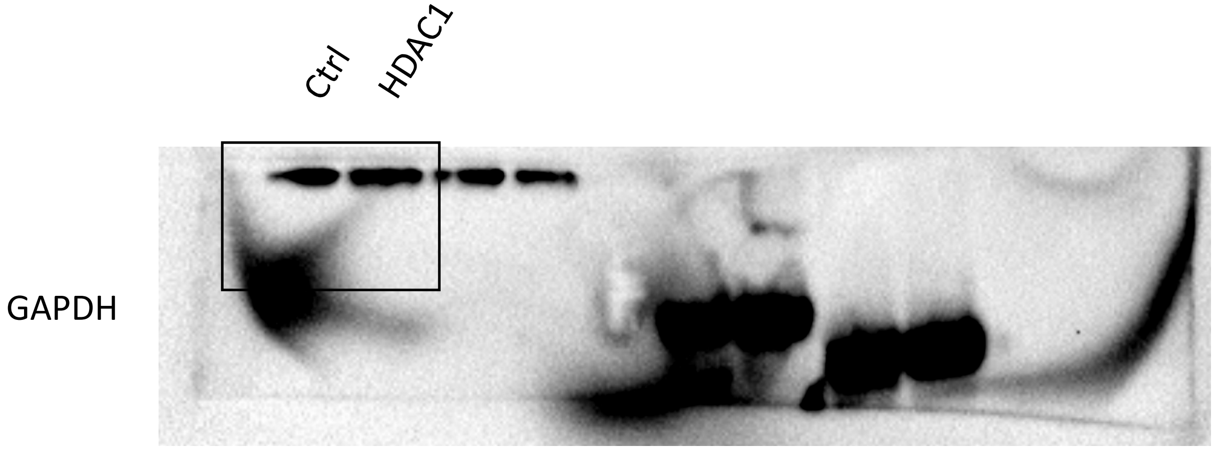

Full unedited gel for Figure 6b

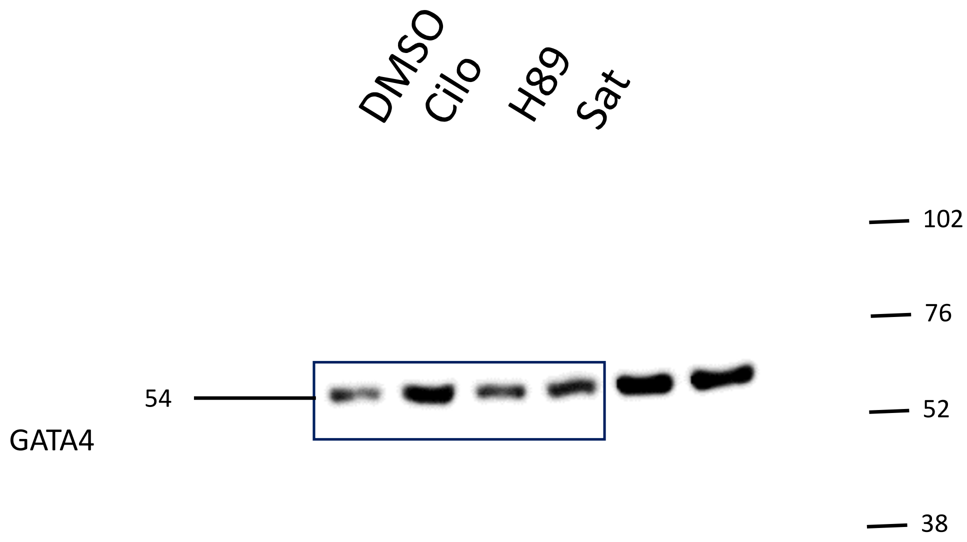

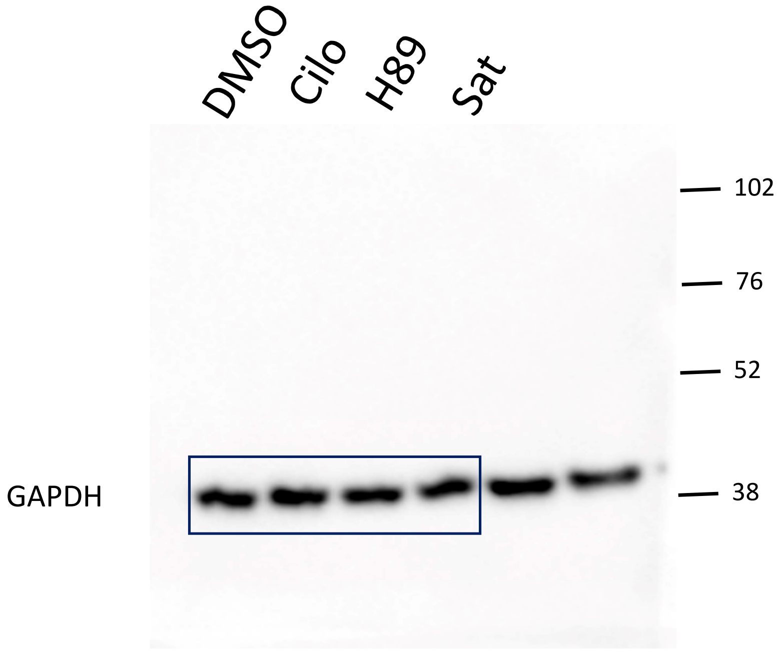

Full unedited gel for Figure 6c

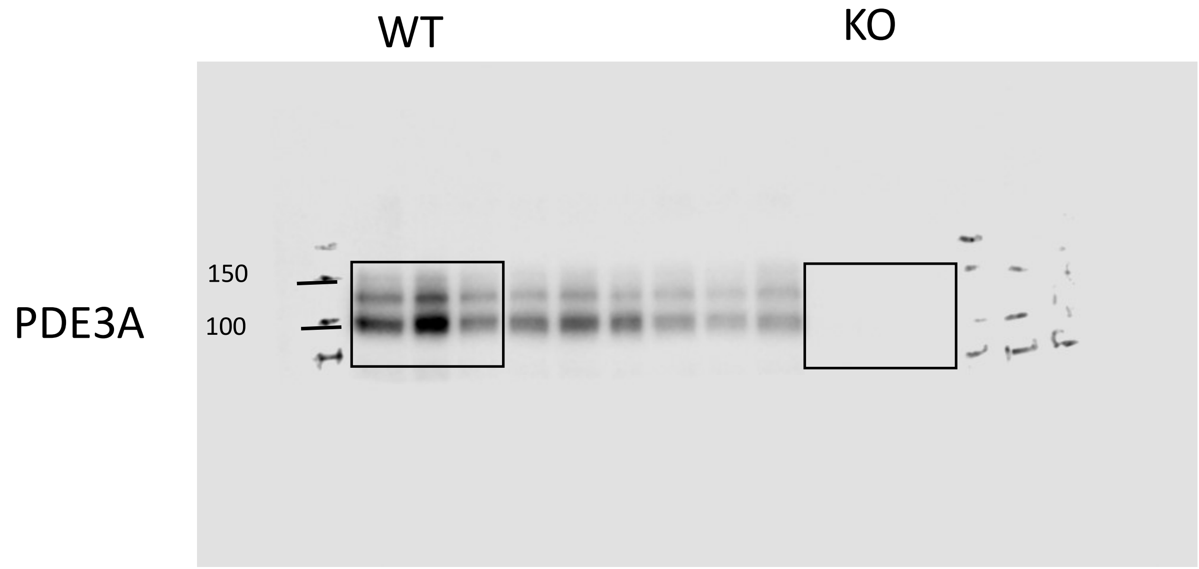

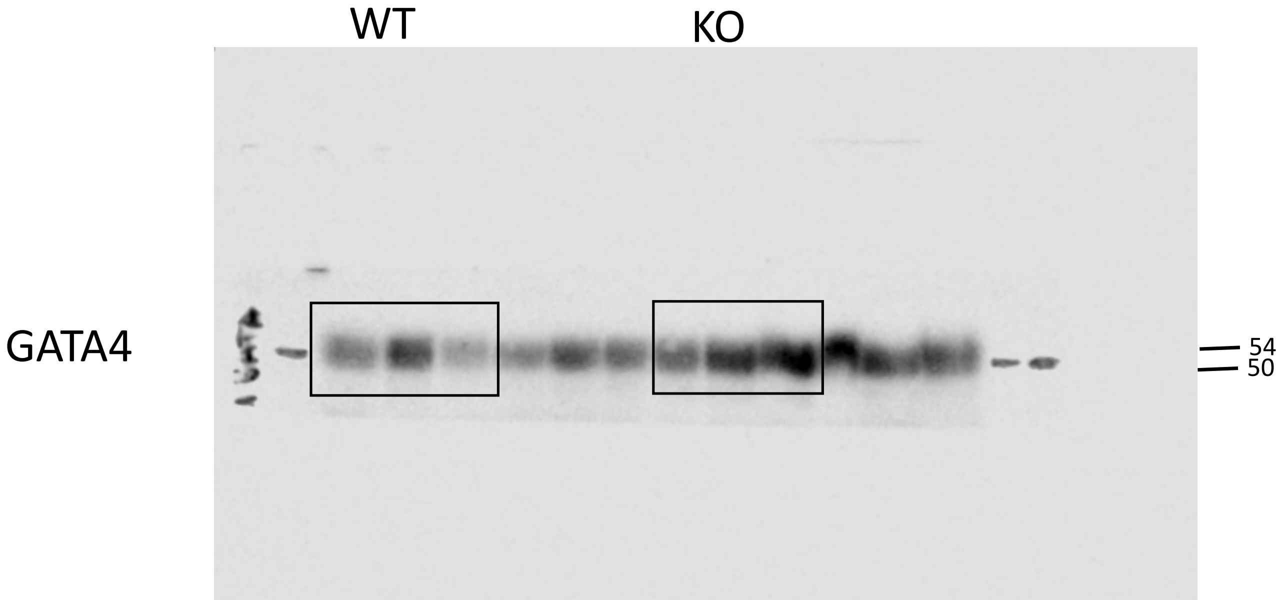

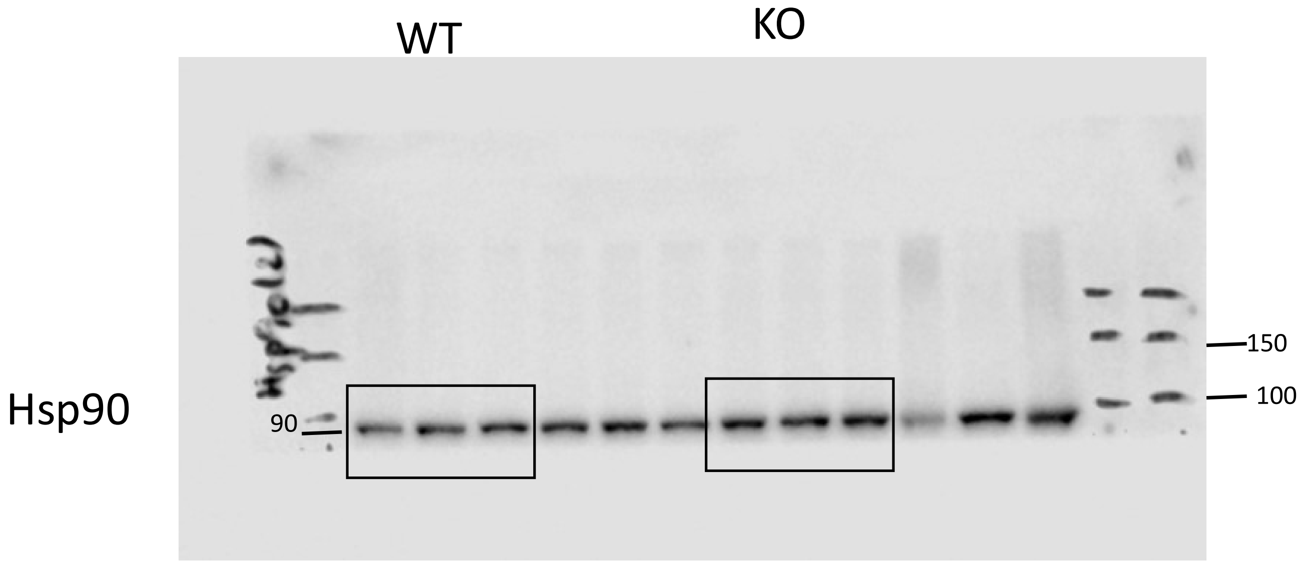

Full unedited gel for Figure 7a

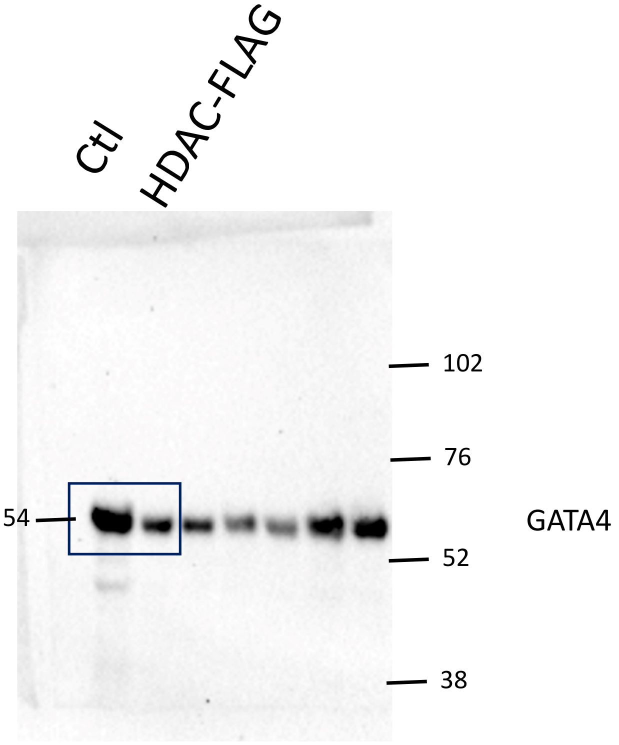

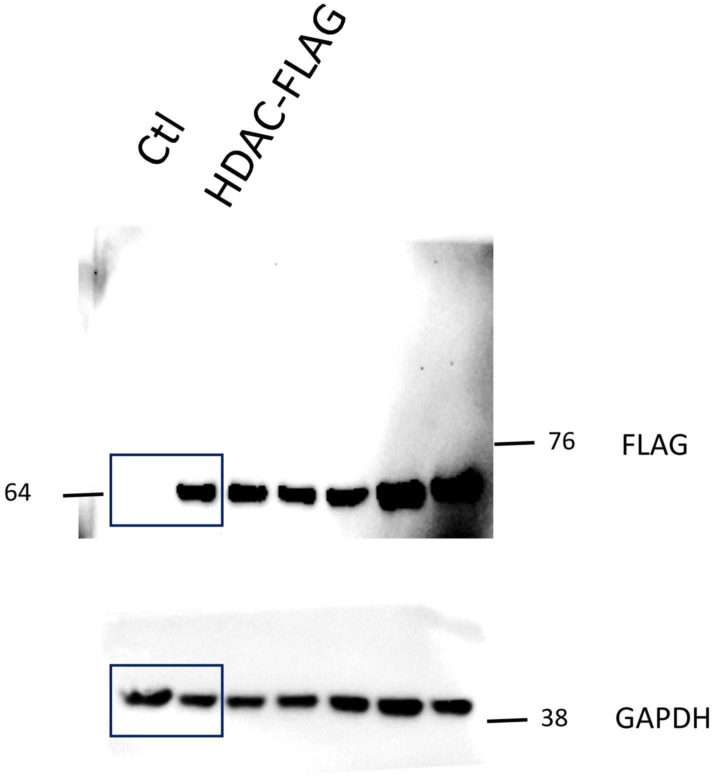

Full unedited gel for Figure 7b

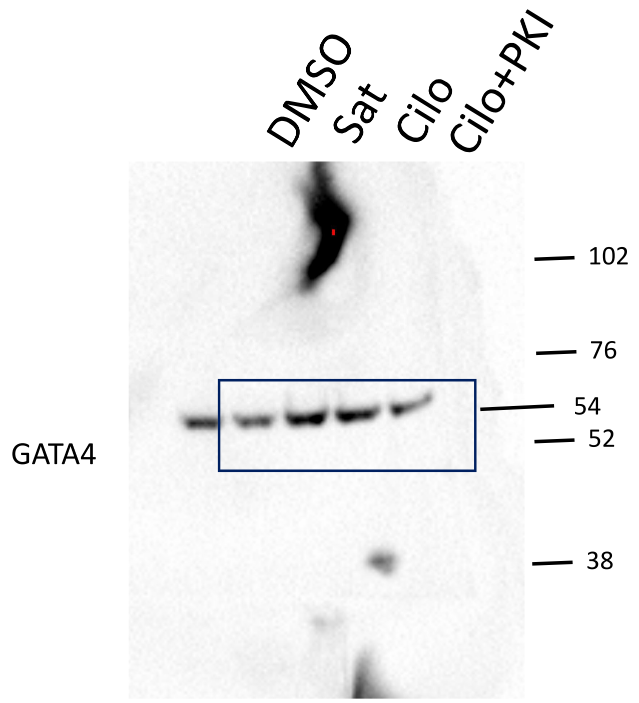

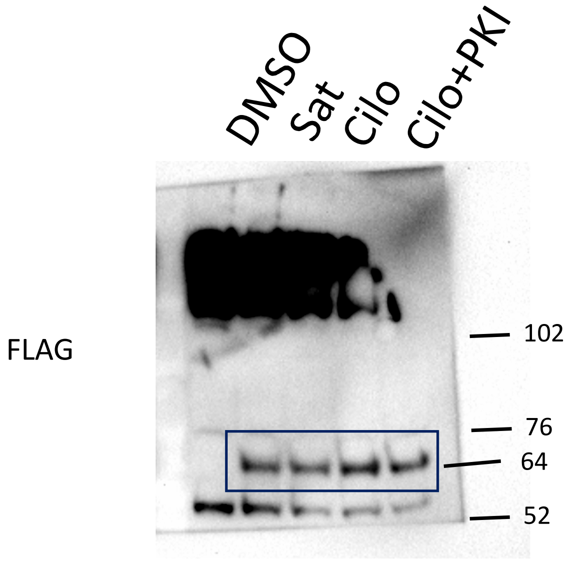

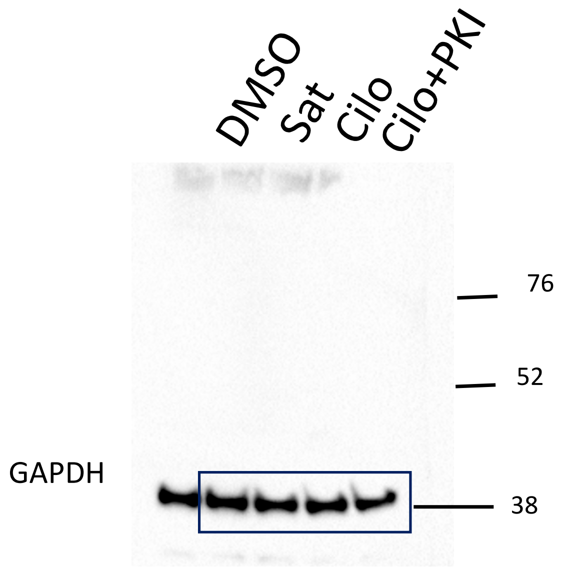

Full unedited gel for Figure 7c

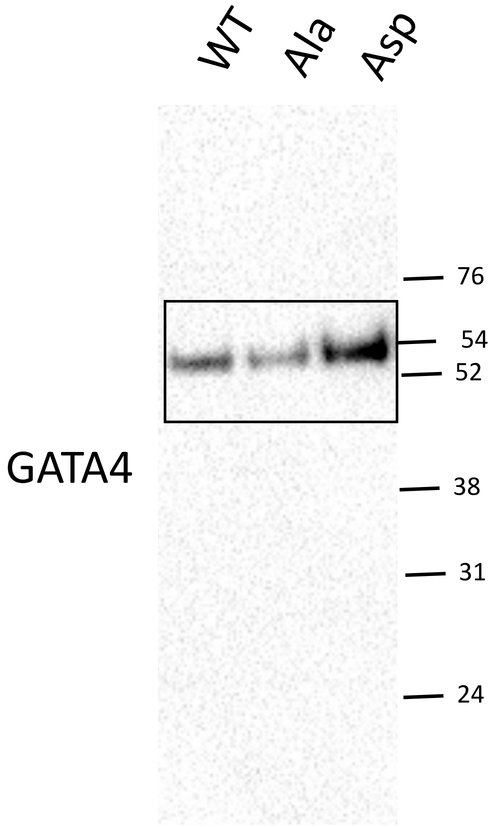

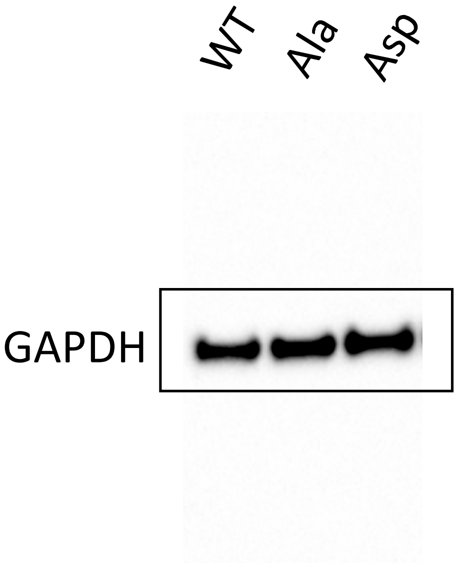

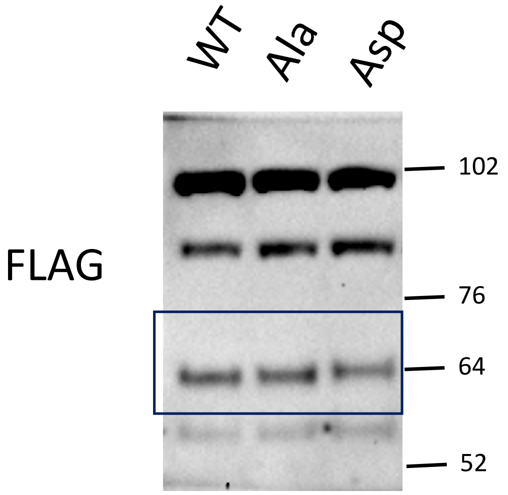

Full unedited gel for Supplementary Figure 1b

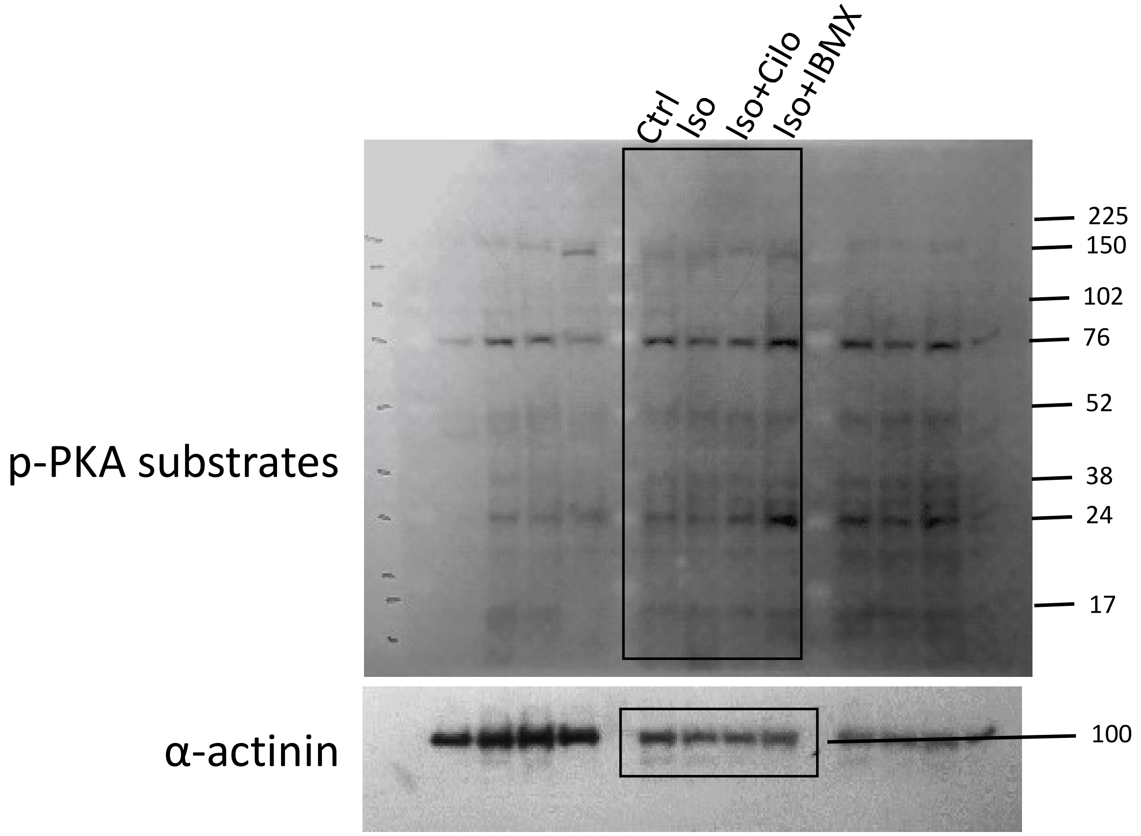

Full unedited gel for Supplementary Figure 1d

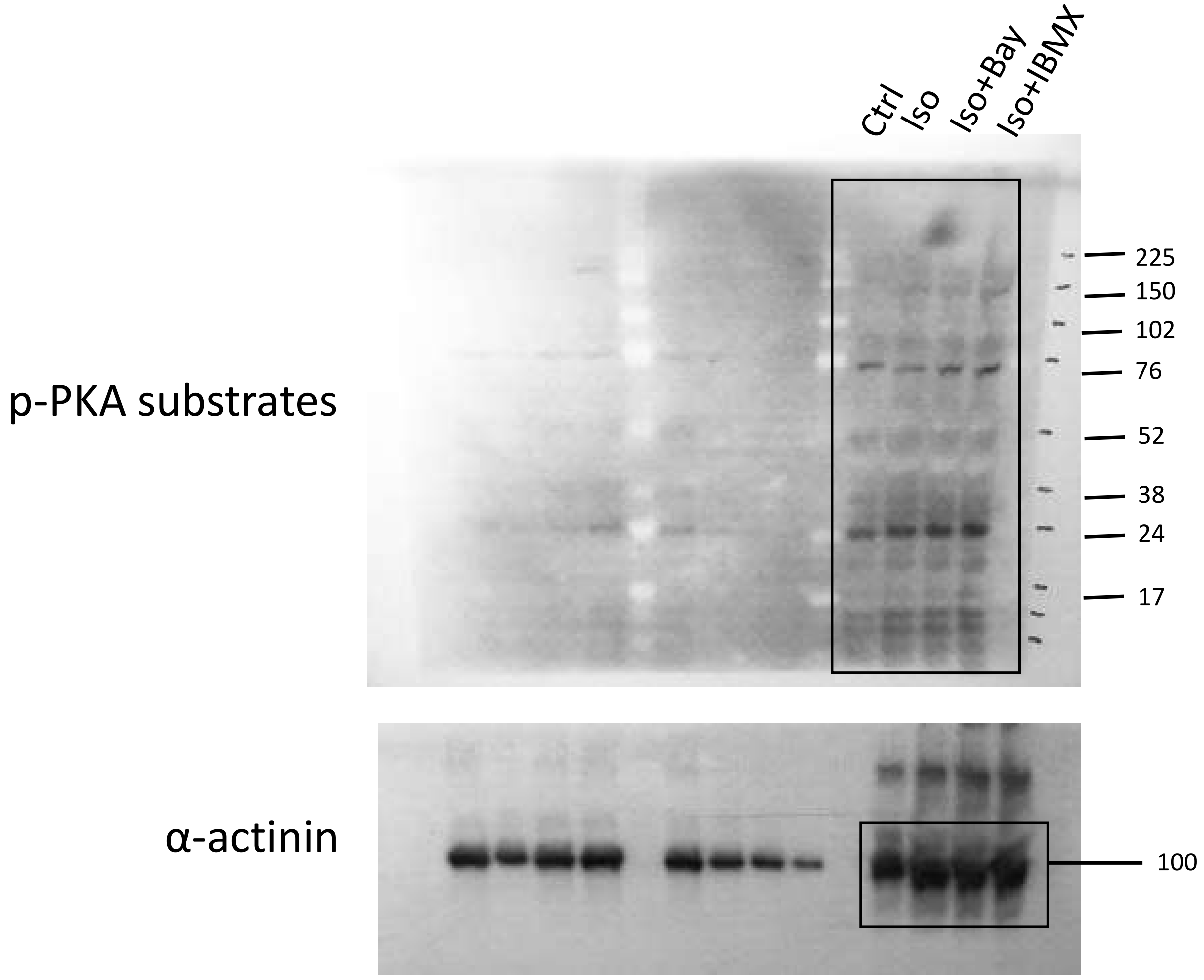

Full unedited gel for supplementary Figure 8a

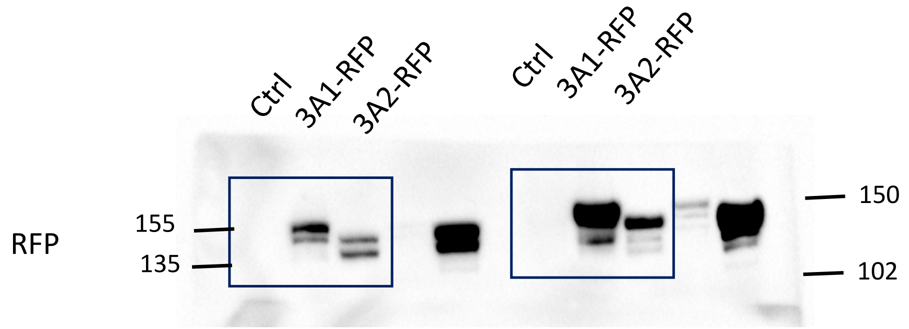

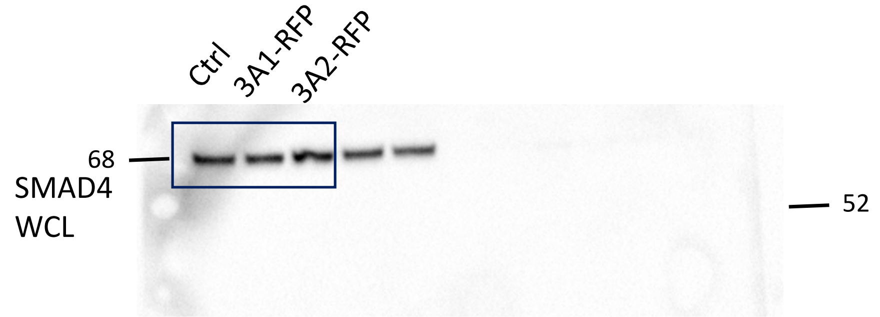

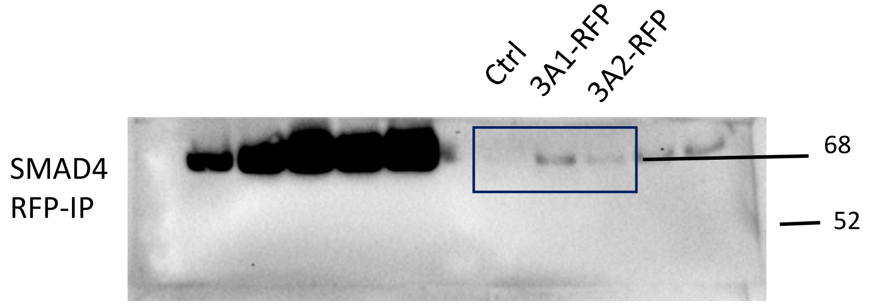

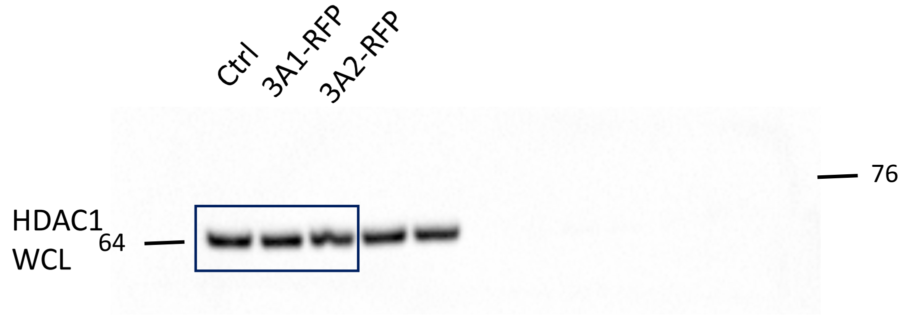

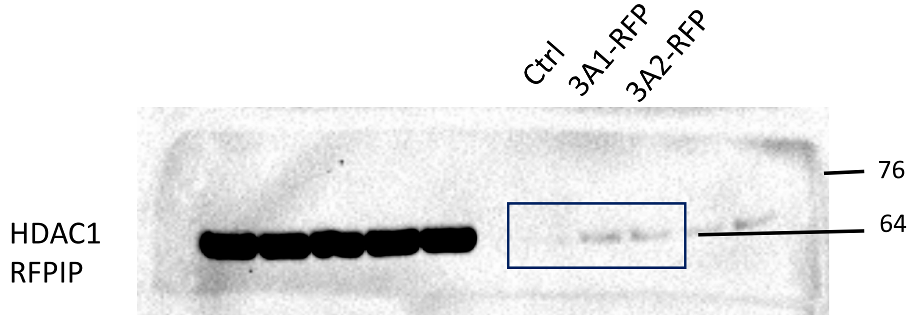

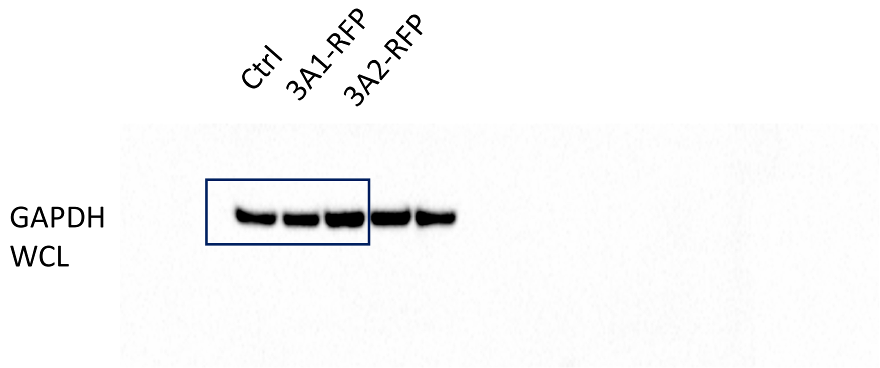

Full unedited gel for supplementary Figure 8b

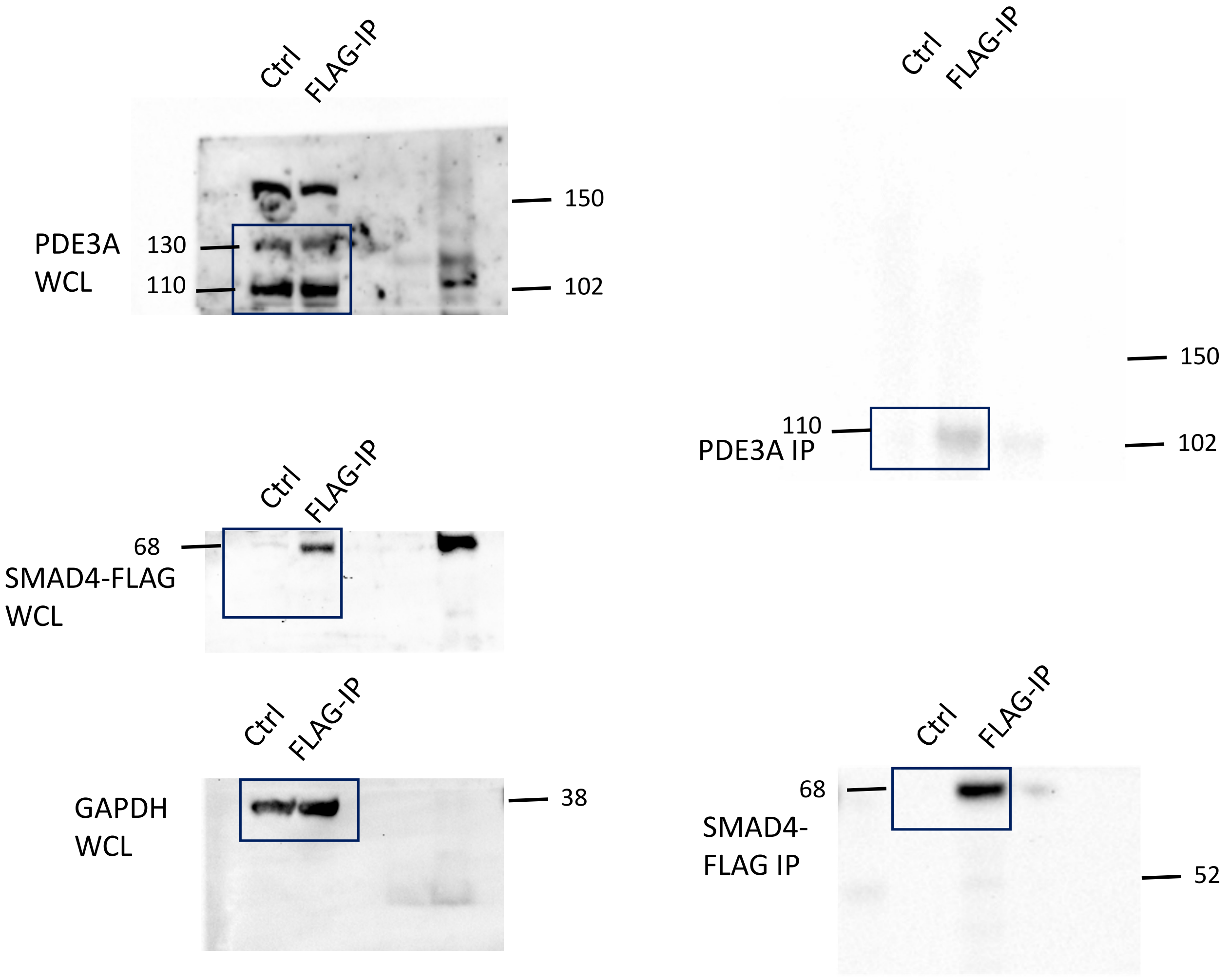

Full unedited gel for Supplementary Figure 8C

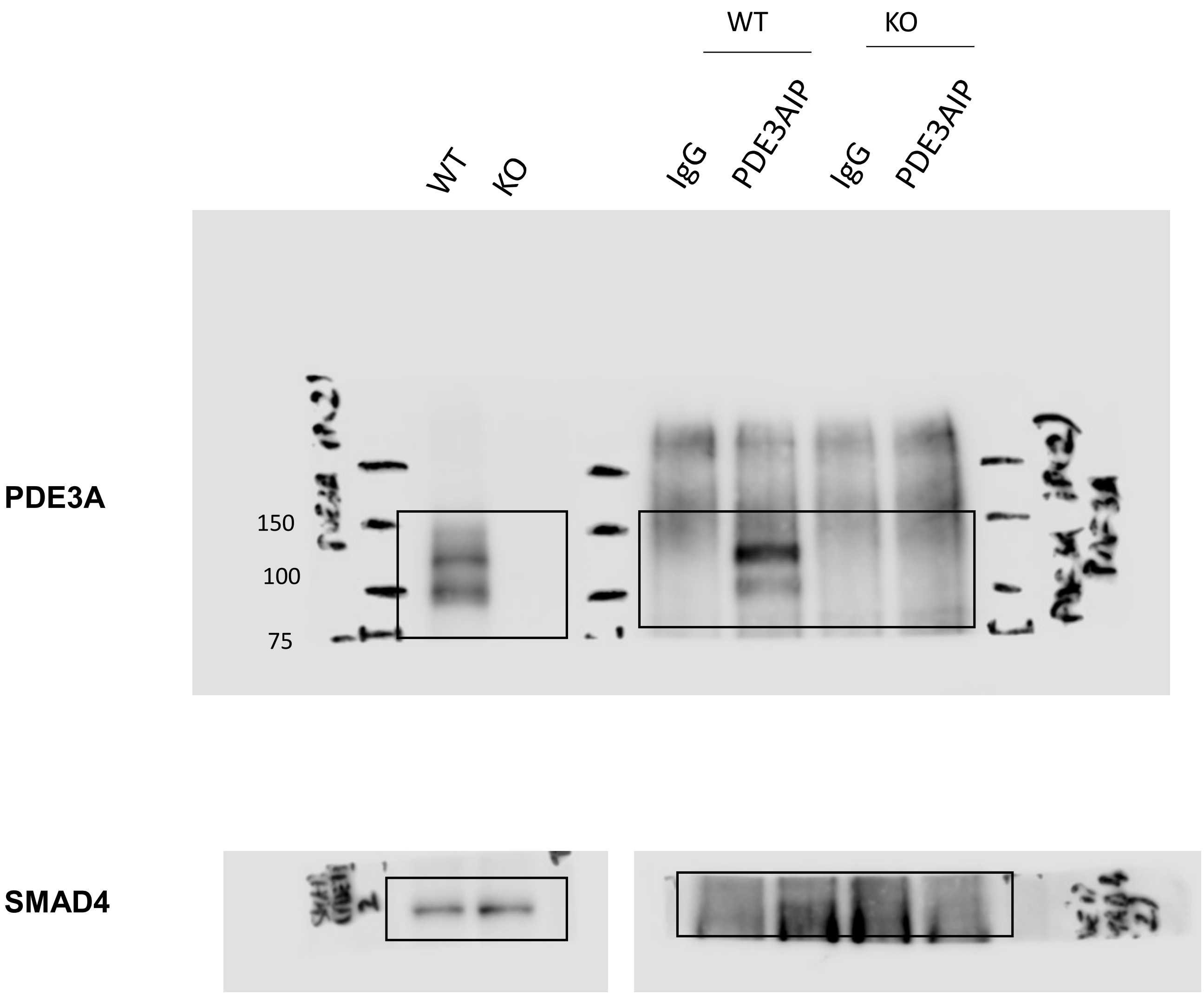

Full unedited gel for Supplementary Figure 8d

RFP 3A2 (WCL and IP)

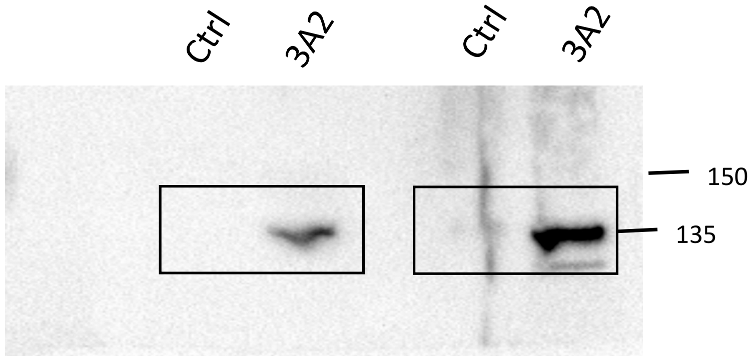

SMAD4 (WCL and IP)

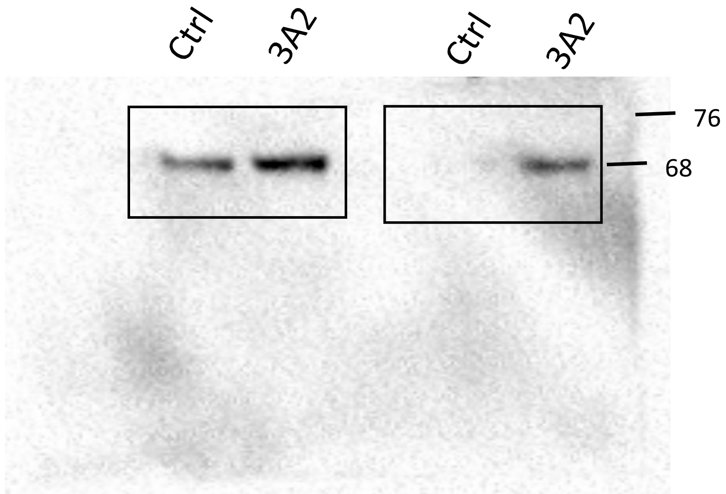

HDAC1 WCL and IP

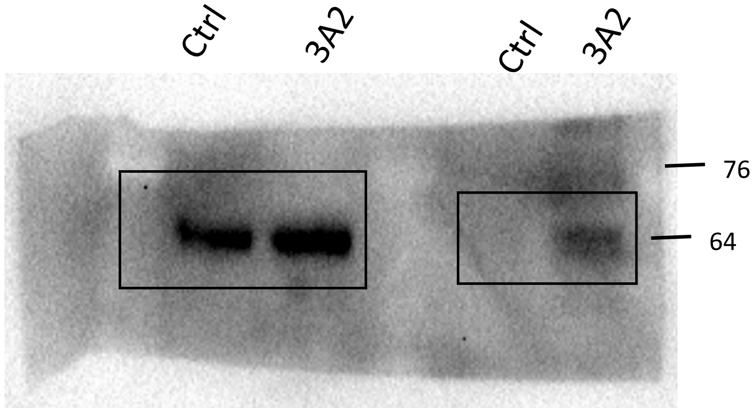

Full unedited gel for Supplementary Figure 8e

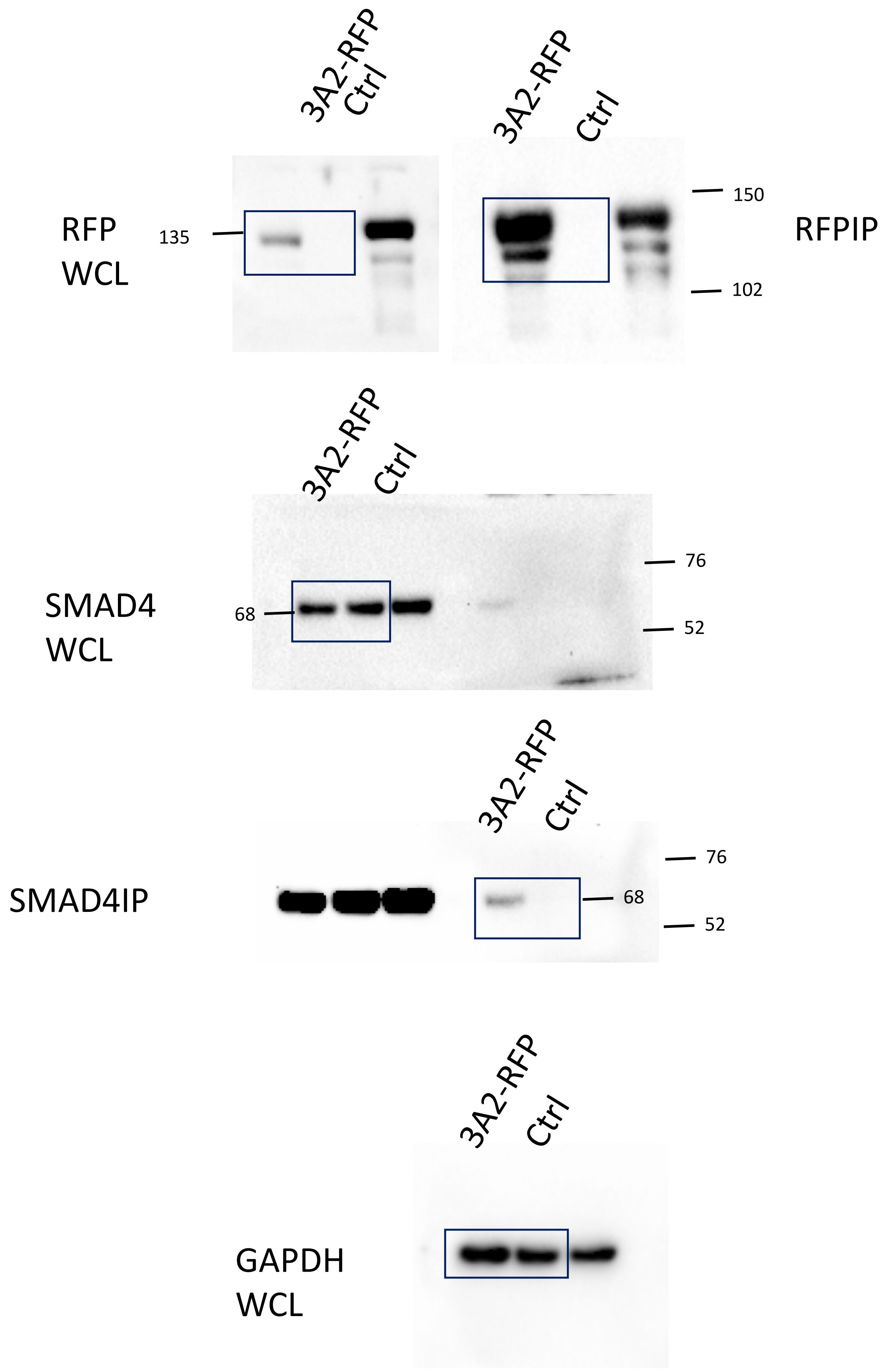

Full unedited gel for Supplementary Figure 9

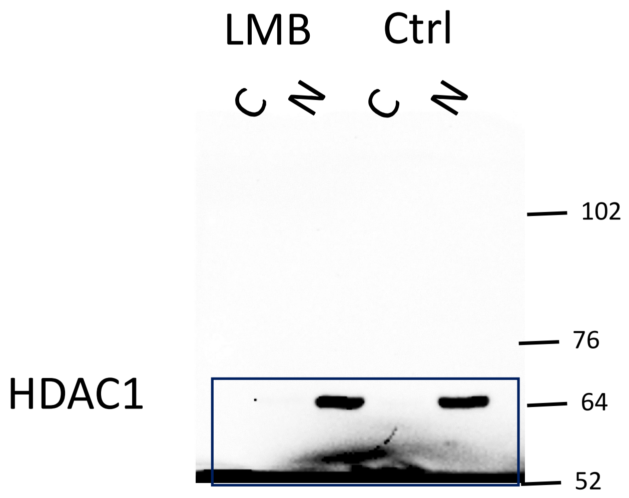

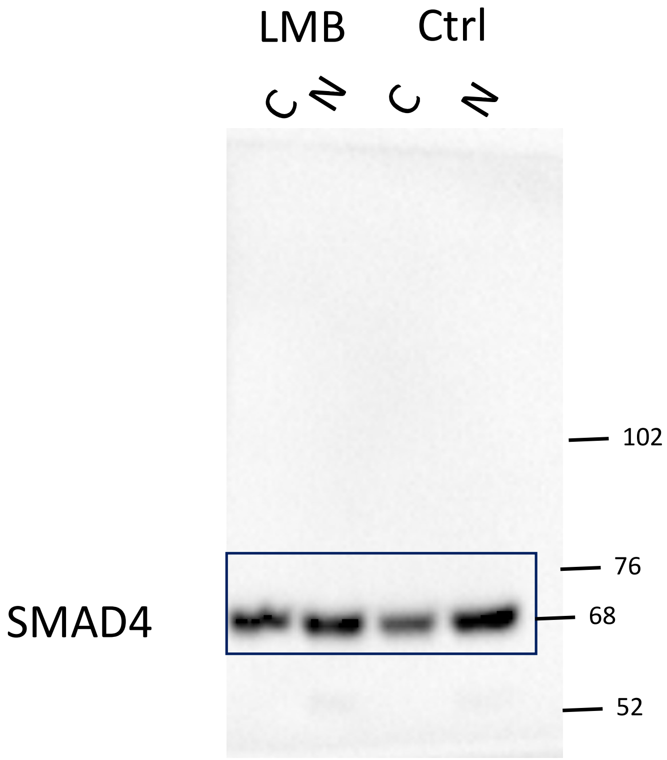

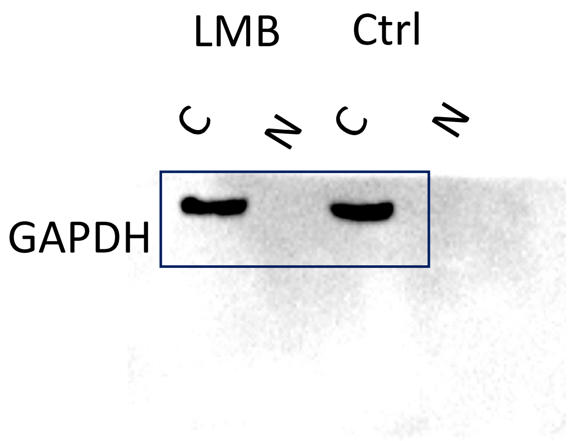

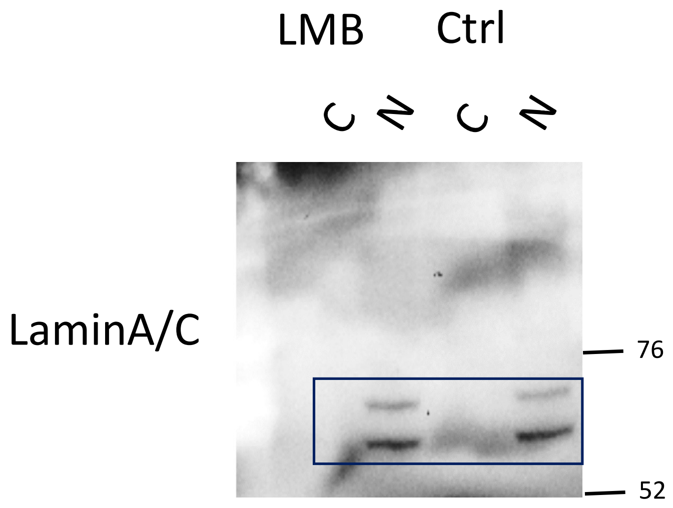

Full unedited gel for Supplementary Figure 11a

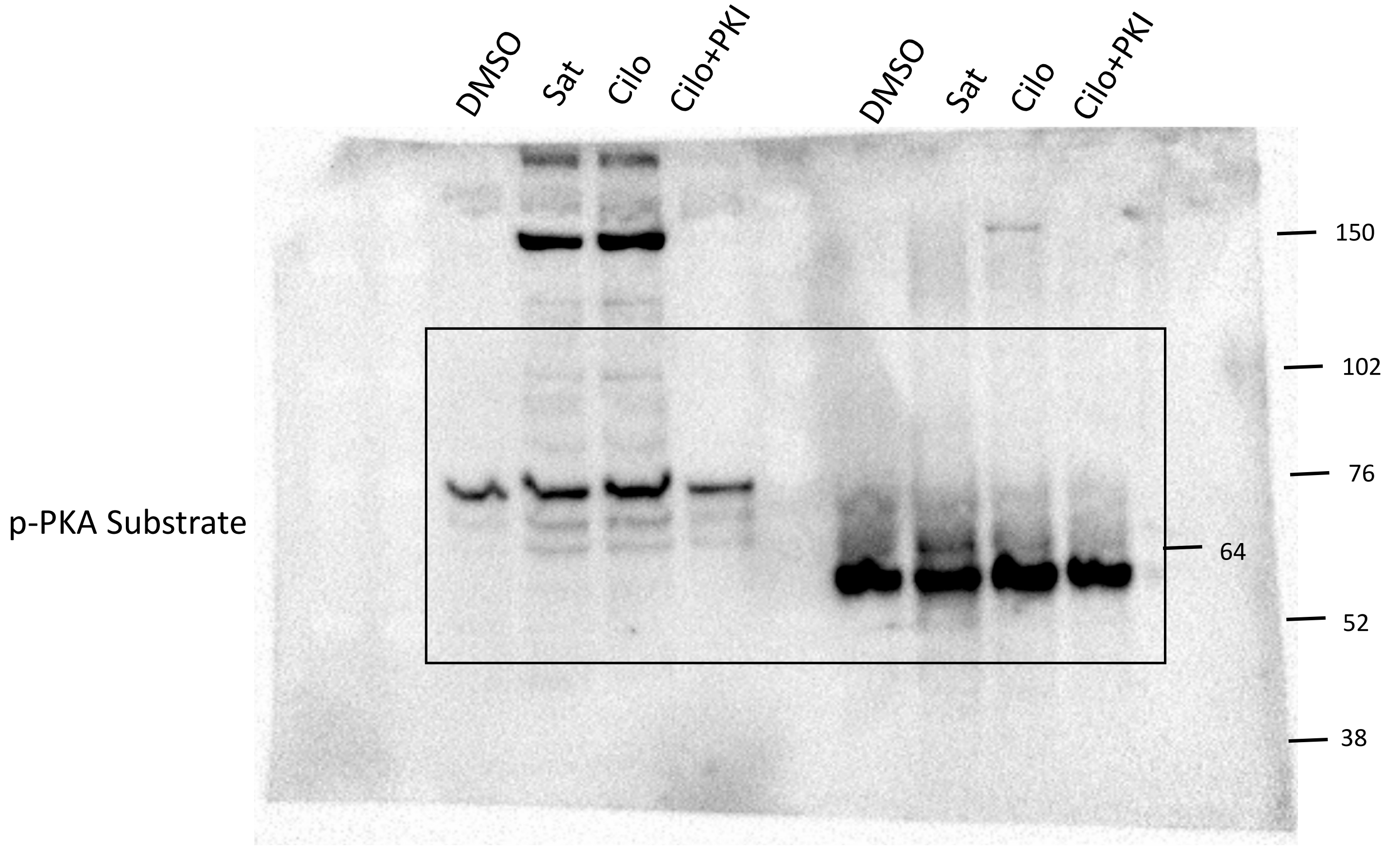

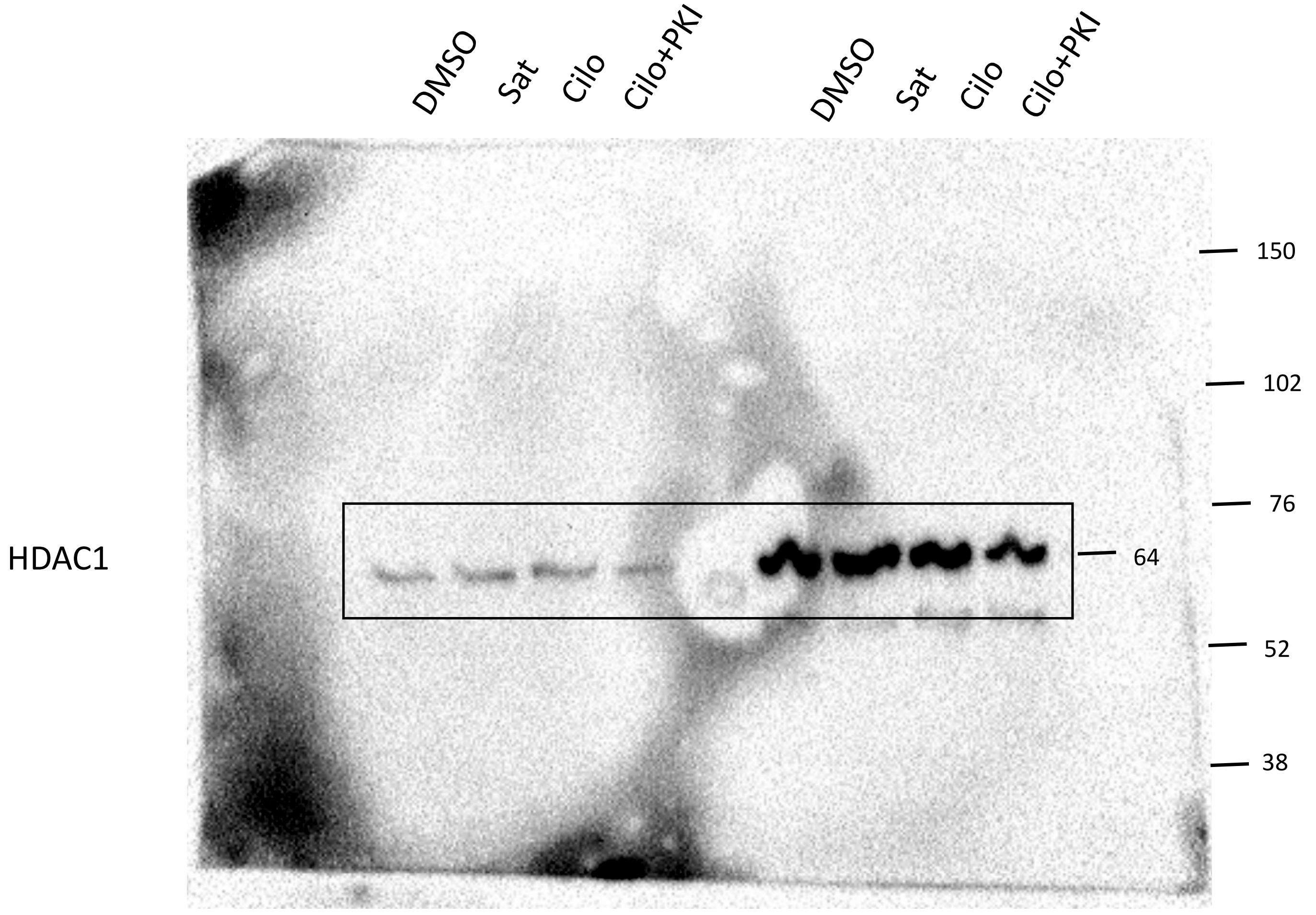

Full unedited gel for Supplementary Figure 11b

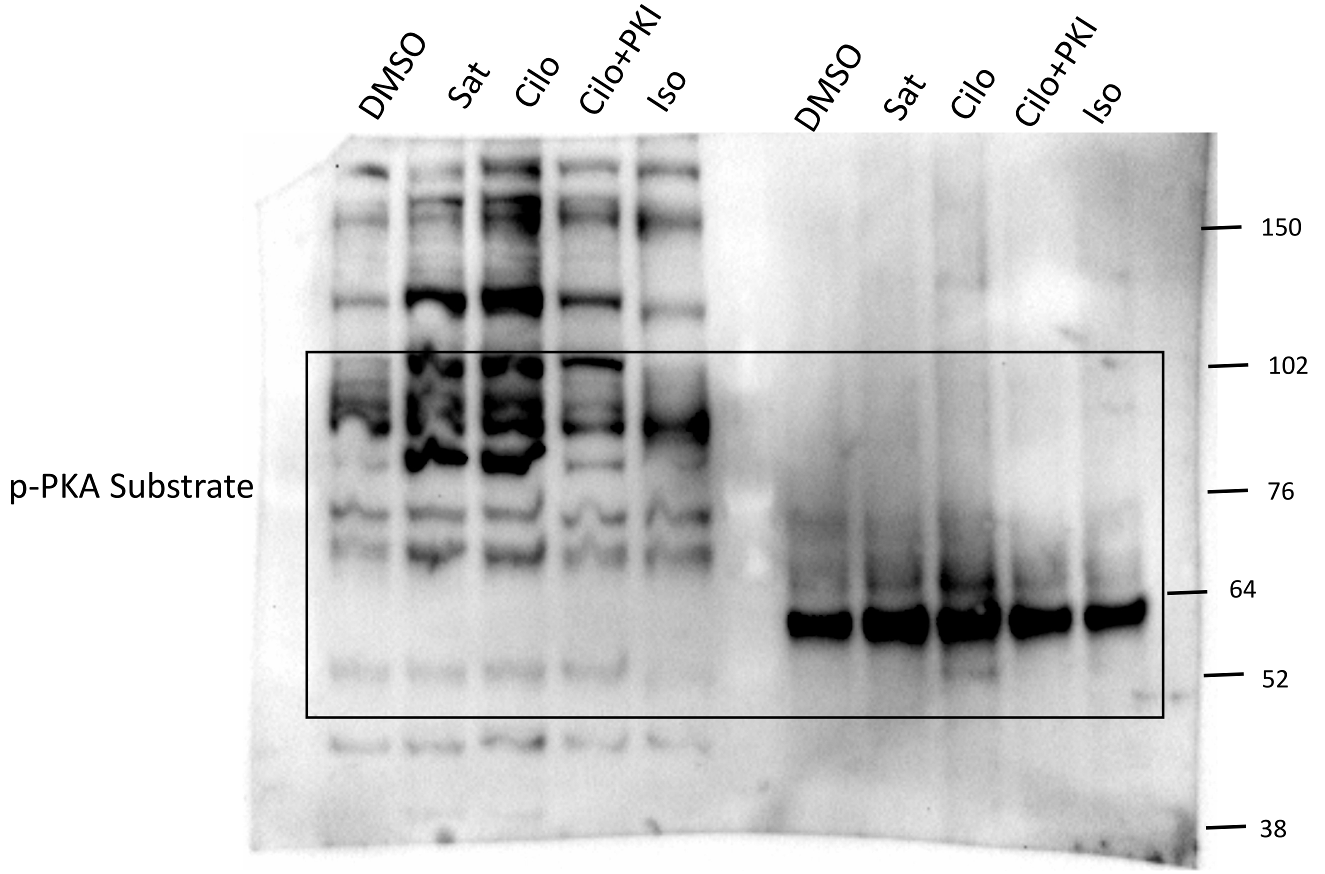

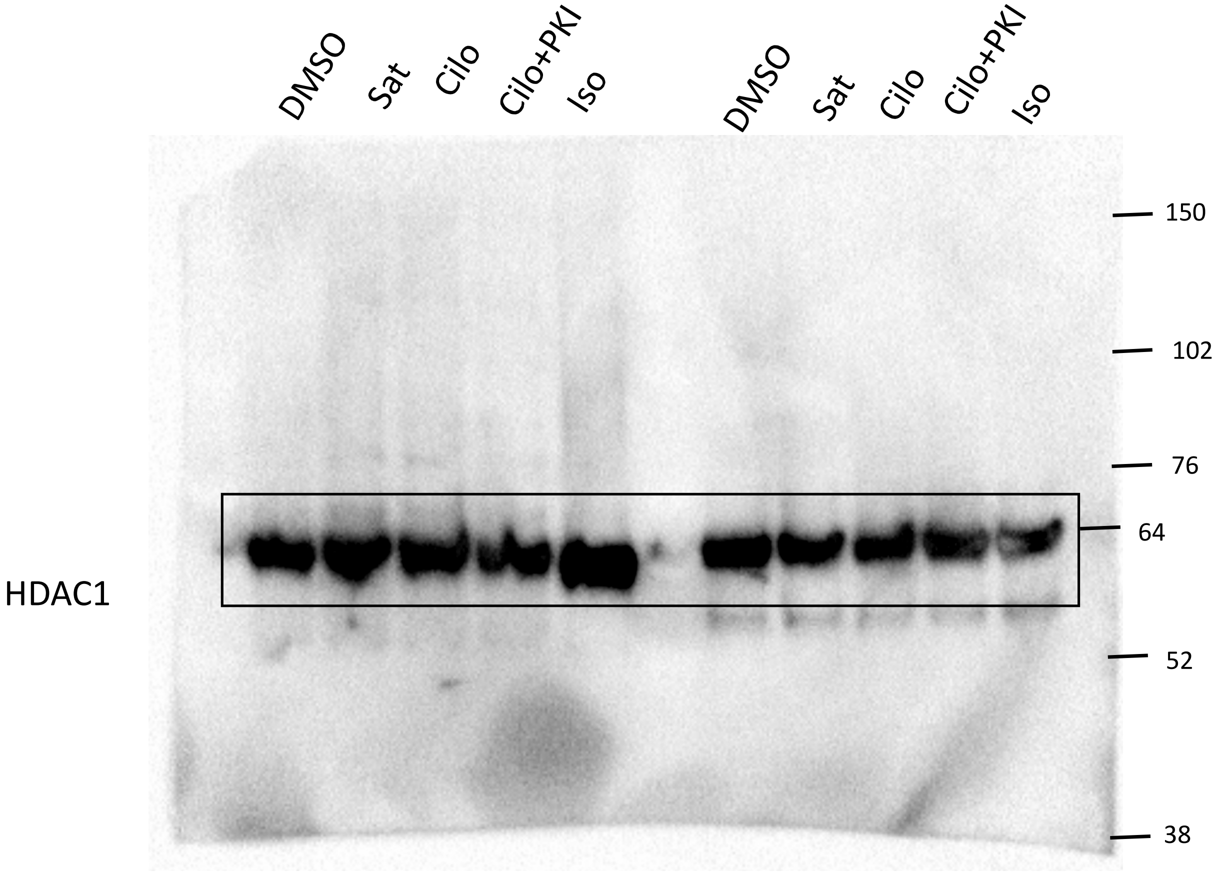

Full unedited gel for Supplementary Figure 11c

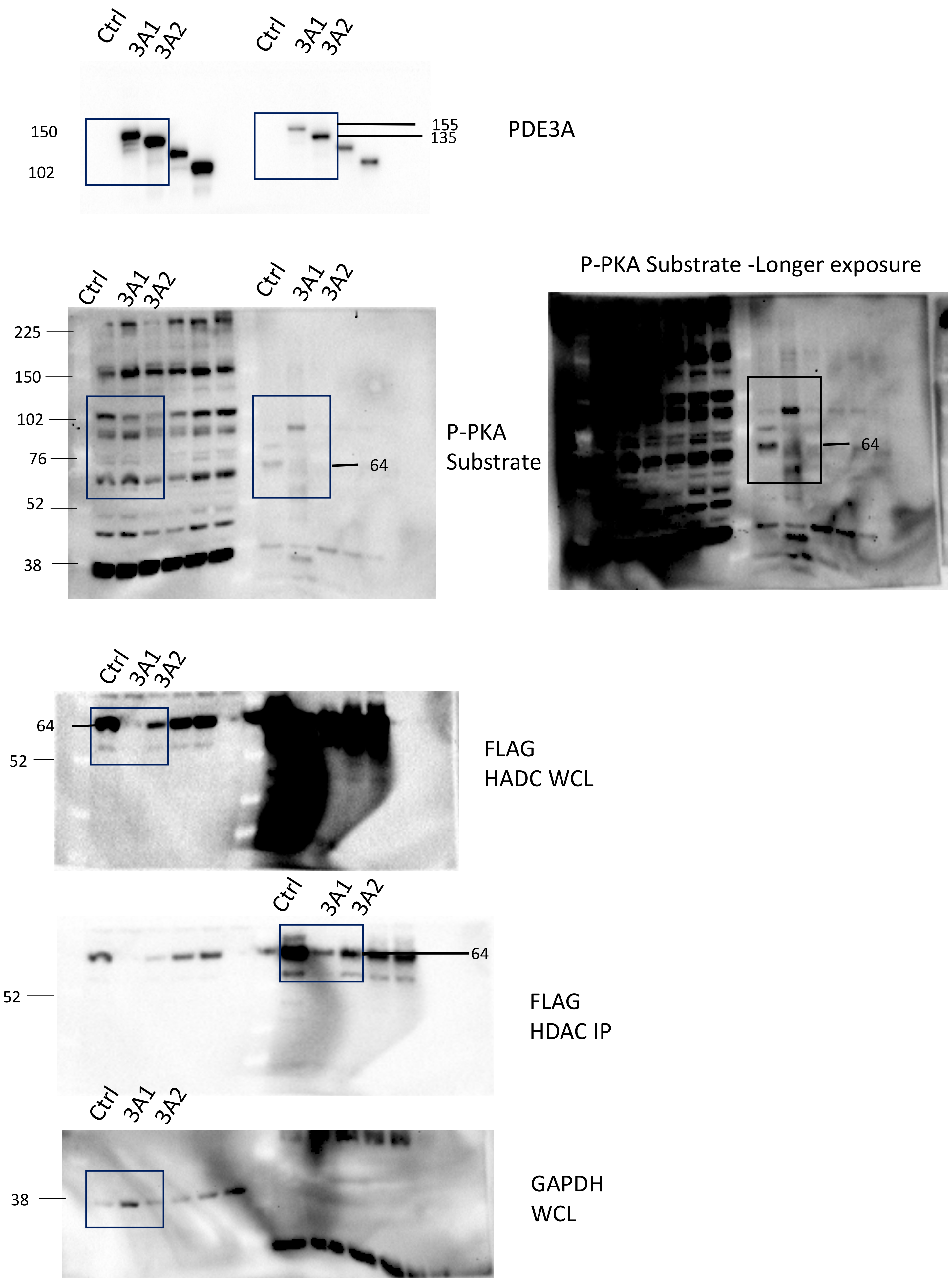

Full unedited gel for Supplementary Figure 12

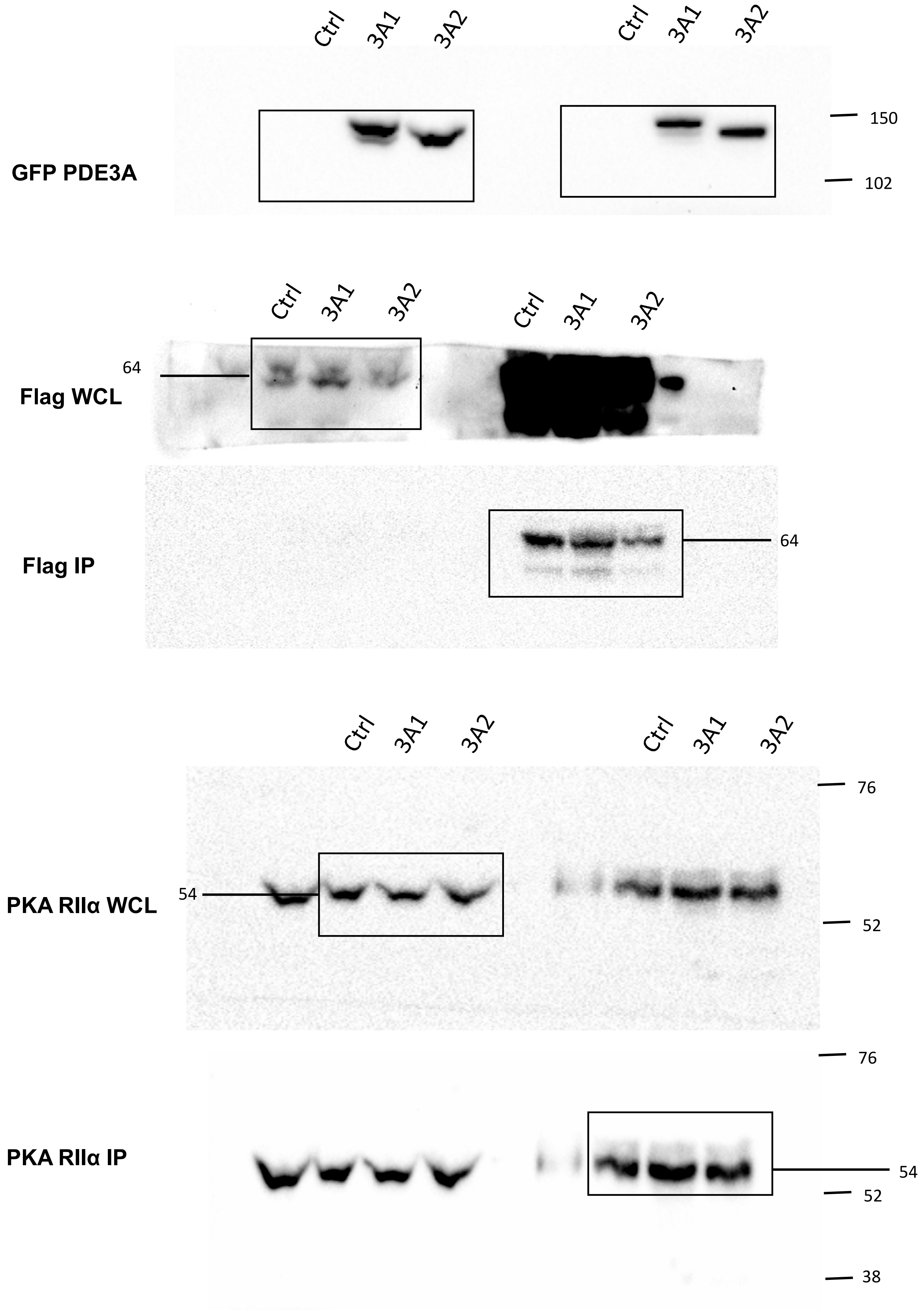

Full unedited gel for Supplementary Figure 12

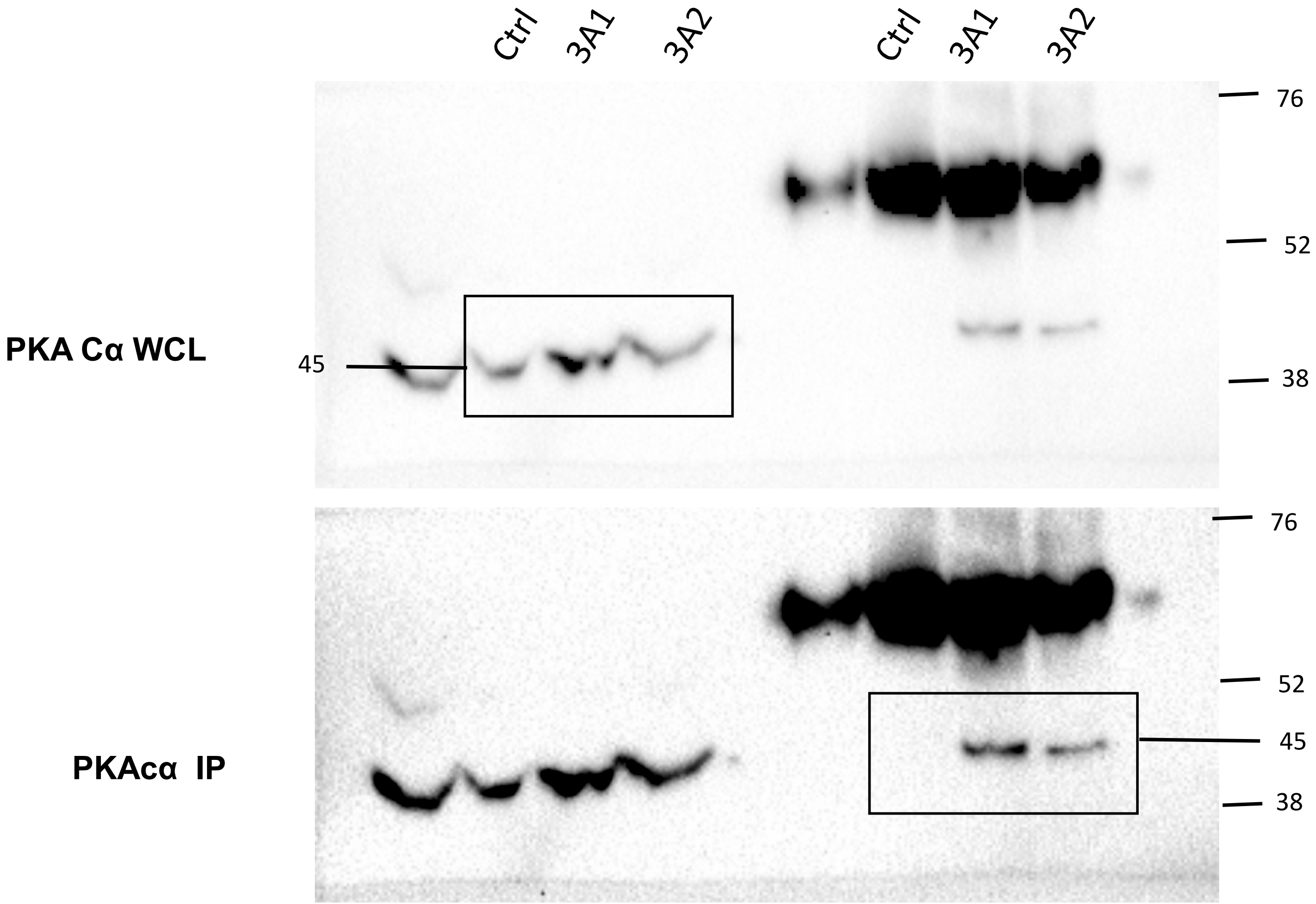

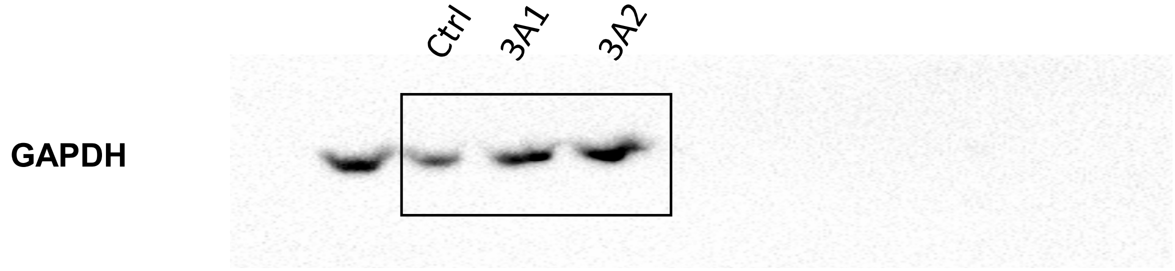

Full unedited gel for Supplementary Figure 13a

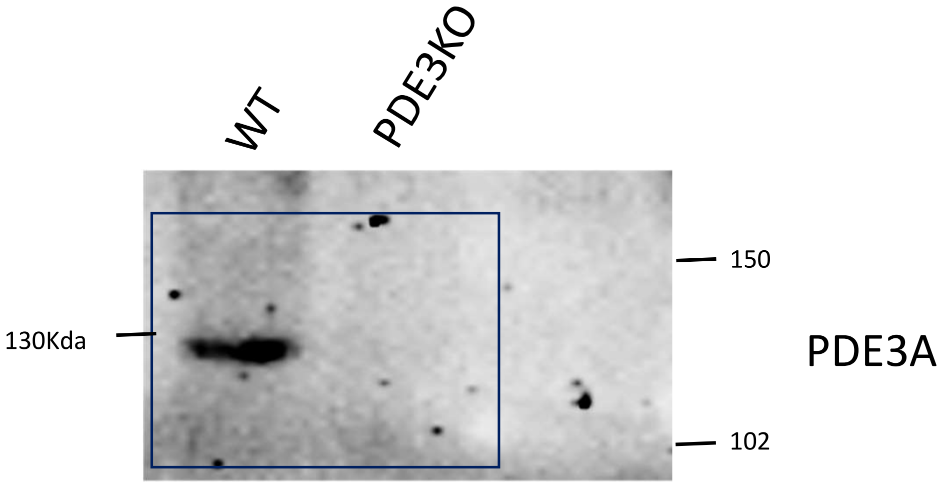

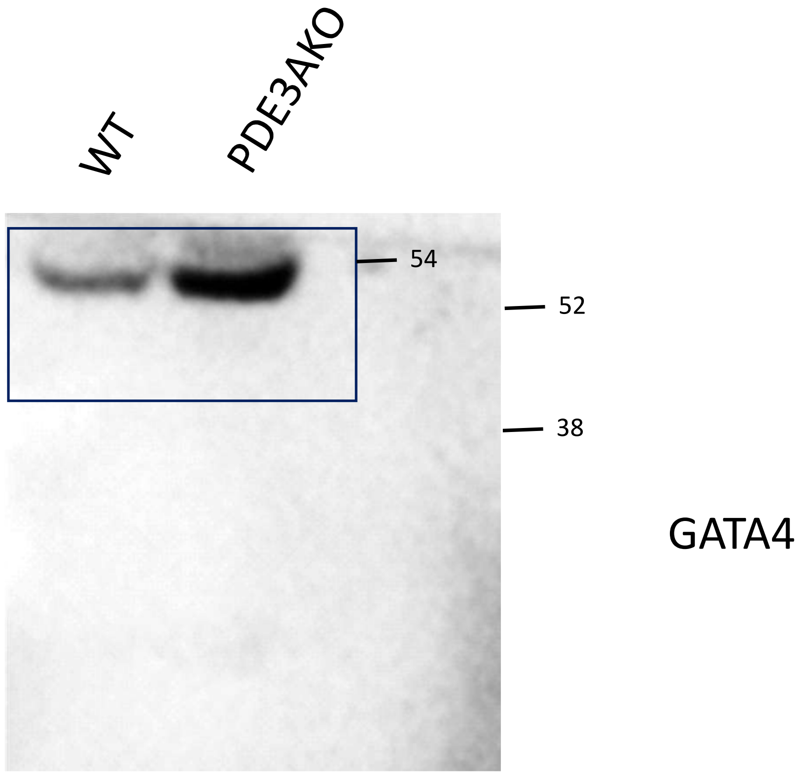

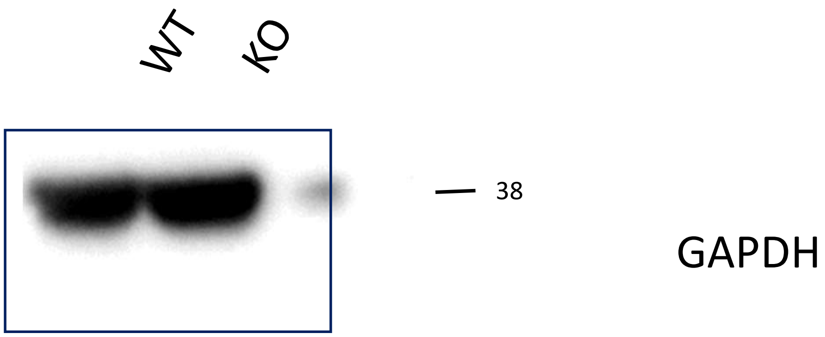

Full unedited gel for Supplementary Figure 13e

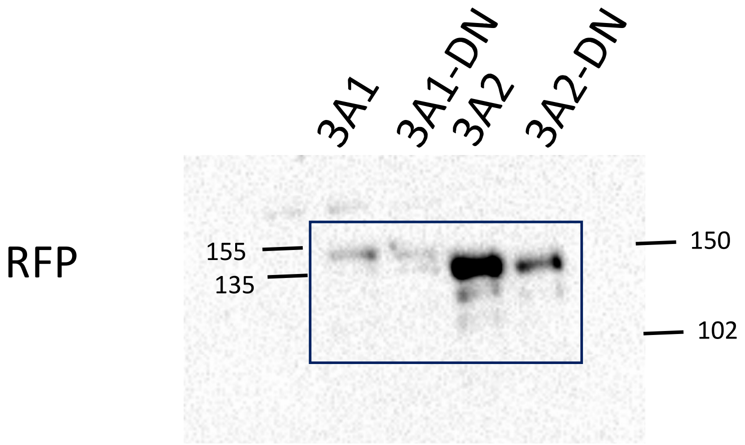

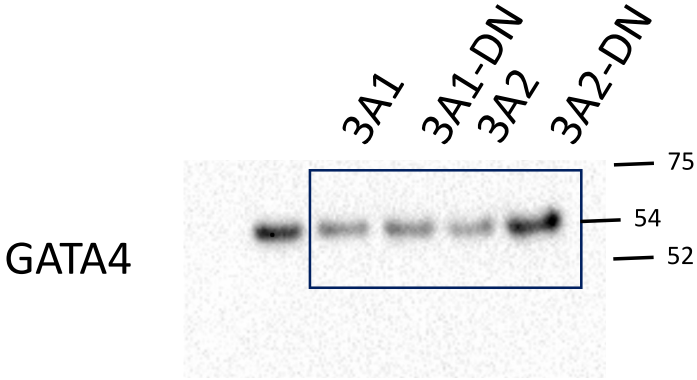

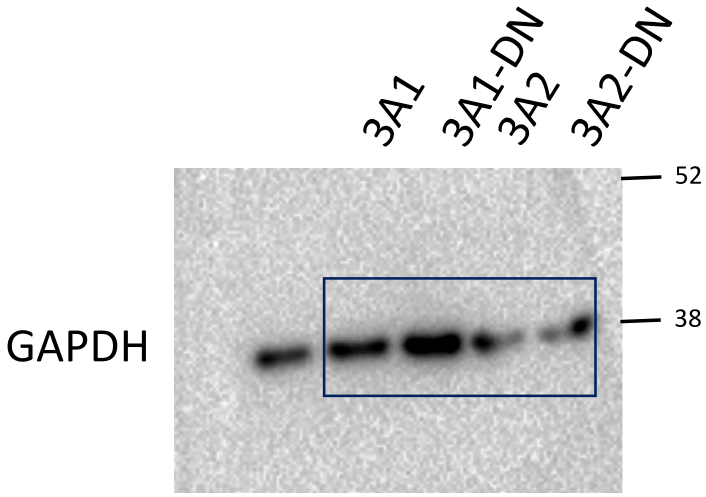

Supplement: Supplementary file 7 [file res-132-828-s007.pdf]
